# Global Search for Stable C_4_H_5_NO Compounds—Guinness Molecules and Stability Islands

**DOI:** 10.3390/molecules28020728

**Published:** 2023-01-11

**Authors:** Oleg A. Mikhaylov, Ilya D. Gridnev

**Affiliations:** N. D. Zelinsky Institute of Organic Chemistry, Leninsky Prosp. 47, 119991 Moscow, Russia

**Keywords:** Guinness molecules, relative stabilities, N-O heterocycles, cyanoketones, singlet carbenes, isonitriles

## Abstract

Global reaction route mapping (GRRM) analysis for compounds with the formula C_4_H_5_NO allowed for the detection of the corresponding “Guinness molecules” **000** and **001**, as well as around 150 other stable minima of the same composition. The results suggest that compounds of similar functionality form a kind of “Stability Island” with their free energies of formation falling within s relatively limited range.

## 1. Introduction

The term “Guinness molecules” was introduced in 2014 by Suhm to describe the most stable molecule for a certain chemical formula [1]. It was argued that the results of a systematic search for such molecules (which inevitably must involve the ranking by energy of all other molecules with the same composition) could be useful for the astronomical search for interstellar molecules, prediction of X-ray structures, etc. However, eight years later, we are aware of only one report exploiting the idea of a “Guinness molecule” devoted to the study of low molecular weight carbohydrates [2]. Interestingly, it was found in this study that the most stable structures of molecules of the formula CnH_2_nOn, up until n = 5, are small molecules aggregates rather than conventional molecules.

As we are interested in the concept of “Guinness molecules” and in the chemistry of relatively small molecules characterized by a high reactivity, we have chosen the sum formula of C_4_H_5_NO for a systematic search for corresponding stable molecules and the Guinness molecule.

A useful tool for such kind of computations is the GRRM (global reaction routes mapping) software developed by Ohno et al. [3,4,5]. GRRM is a computer program for automated exploration of chemical reaction pathways. It can be used for reaction route mapping for the potential surface of a certain chemical formula. Starting from an equilibrium structure, an automated search of dissociation and isomerization reactions can be performed [6,7,8,9,10].

## 2. Results and Discussion

### 2.1. Computational Details

**Figure 1:**
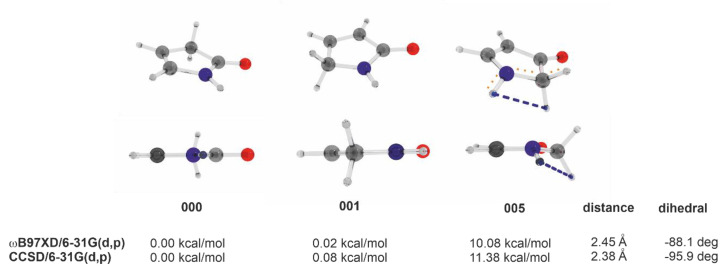
Relative Gibbs free energies and selected structural parameters for two “Guinness molecules” of C_4_H_5_NO and their unstable positional isomers. Hydrogen: light grey, Carbon: black, Nitrogen: blue, Oxygen: red.

We used the GRRM-12 version of the software for a global search of configurational minima of the formula C_4_H_5_NO. The Gaussian 09 software package [11] was used for optimizations using the BLYP3/6-31G(d) level of theory. A HPC workstation equipped with 64 processors and 256 Gb of memory carried out the task for 163 days, and complete conversion was not reached. Hence, we cannot report all possible equilibrium structures on the potential surface. Nevertheless, we can confidently claim the detection of two “Guinness molecules” with a negligible difference in their Gibbs free energies (see Table 1, compounds **000** and **001**, and Figure 1) and over 140 other compounds of C_4_H_5_NO in 14 different classes (see Tables S1–S14). 

In total, 489 minima were located during the GRRM-12 implementation. This number included numerous conformers. In most cases, only the most stable conformer was further reoptimized for inclusion in the tables. Only a very limited number of conformers were reoptimized and included in Table S1.

All conformational minima were reoptimized using the ωB97XD functional [12] with the 6-31G(d,p) basis set. Mostly only singlet multiplicity was considered. In the cases of isonitriles and carbenes, the stability of the wavefunction was checked prior to optimization and when necessary, the stable=opt option was used, leading to singlet multiplicities in all cases. ZPVE energies were unscaled. Note that the relative values of E_ZPVE_, H and G were very close for each structure (Tables S1–S14).

Despite the lack of conversion of the GRRM computation, the data was good enough to build a free energy map for C_4_H_5_NO molecules. All computed structures are listed in Tables S1–S14, whereas Table 1 shows the most and the least stable isomers for each group of compounds.

### 2.2. Guinness Molecules and 5-Membered Heterocycles of C_4_H_5_NO

We have found that there are two Guinness molecules in the multitude of potential C_4_H_5_NO compounds, viz., γ-lactams **000** and **001** (Figure 1, Table 1). Interestingly, their isomers, with the NH unit positioned near the C=C bond (**005**), are about 10 kcal/mol less stable due to the lack of conjugation of the nitrogen lone pair with the C=C-C=O unit (the nitrogen atom is pyramidal in **005** and flat in **000** and **001**). To check the accuracy of this observation, we recomputed the structures **000**, **001** and **005** using coupled-cluster calculations [13] with both single and double substitutions [14], see Figure 1 for the results.

As can be seen from Figure 1, the results of the higher level computations are in accord with the initial observations. The free energy difference between the structures **000** and **001** remains within 0.1 kcal/mol, whereas **005** is more than 10 kcal/mol destabilized compared to the other two. Moreover, the distortions in planarity of the molecule of **005** computed by CCSD are even stronger (Figure 1). Since the coupled-cluster calculations are known to provide an accurate estimation of non-bonding interactions [15], we concluded that: (a) conjugation of the nitrogen lone pair with the adjacent C=C bond is relatively weak and (b) to partially compensate for this effect, the planarity of the molecule **005** is avoided to achieve a closer contact between the NH proton and one of the protons of the nearby CH_2_ group. 

**Figure 2:**
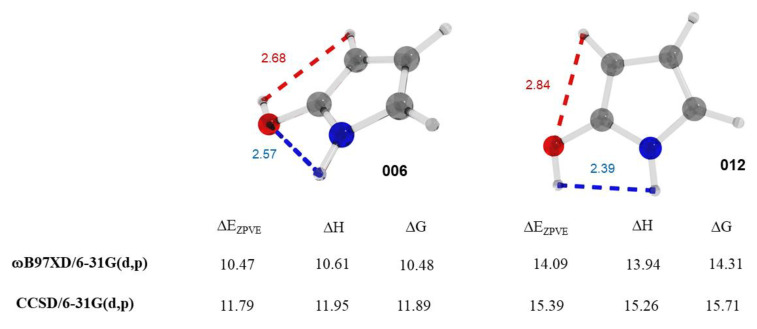
Optimized structures of the enols **006** and **012** (CCSD/6-31G(d,p)), important interatomic distances (Å) and their relative thermodynamic parameters (kcal/mol). Hydrogen: light grey, Carbon: black, Nitrogen: blue, Oxygen: red.

Of interest is the unusual stability of the enol **006**, especially compared to its rotamer **012** (Figure 2). Both molecules are flat. We can conclude that stabilizing effect of the combination of O-H···H-C and C-O···H-N non-bonding interactions in **006** is roughly 4 kcal/mol more effective than that of the combination of O-H···H-N and C-O···H-C interactions in **012**.

**Figure 3:**
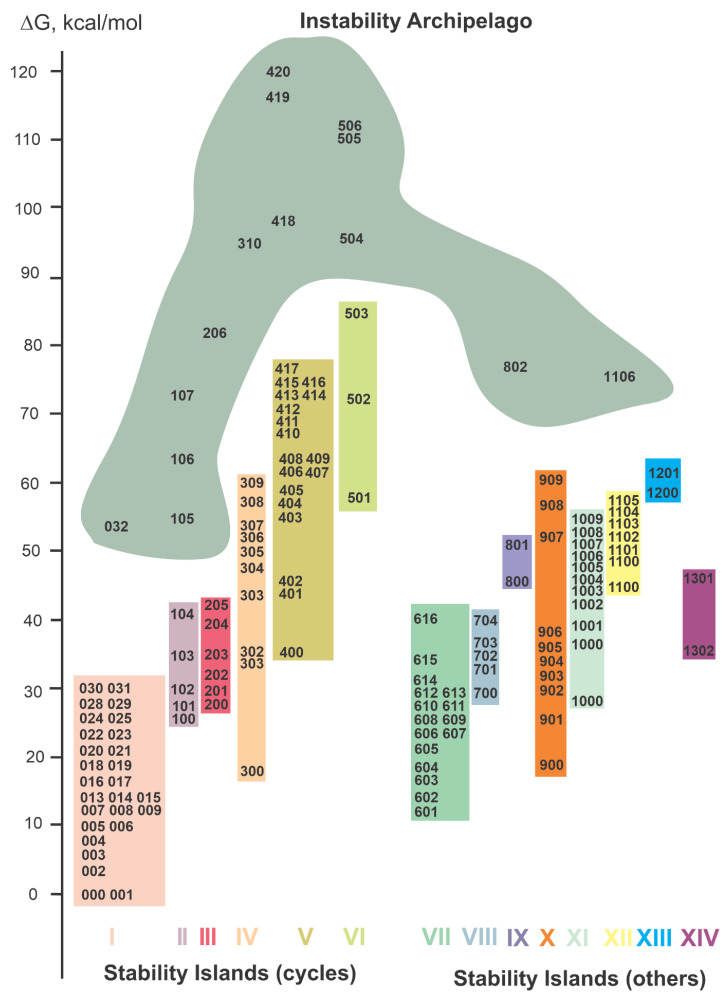
Map of the Gibbs free energies for all located minima for C_4_H_5_NO. I: 5-membered heterocycles; II: 6-membered heterocycles; III: 4-membered heterocycles; IV: saturated 3-membered heterocycles; V: unsaturated 3-membered heterocycles; VI: bicycles; VII: acyclic nitriles; VIII: conjugated trienes; IX: acetylenes; X: allenes; XI: acyclic isonitriles; XII: carbenes; XIII: bipolar compounds; XIV: molecular associates.

In total, the relative Gibbs free energies of about 30 five-membered heterocycles with the formula C_4_H_5_NO (including some rotamers) fall within the region of 30 kcal/mol. We named this region the “Stability Island” (see Figure 3). 

Oxazoles are important heterocycles found in numerous natural compounds and they are biologically active themselves [16]. Several quantum-chemical studies of compounds containing an oxazole ring have been published [17,18,19], but we are unaware of any computations of oxazoles with the formula C_4_H_5_NO.

**Scheme 1:**
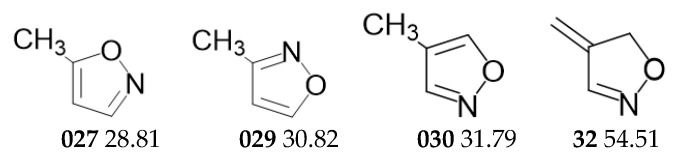
Relatively stable oxazoles containing an N–O bond and their relative Gibbs free energies (kcal/mol).

Due to the lack of aromaticity, the oxazole **032** is approximately 25 kcal/mol less stable than three other oxazoles: **027**, **029** and **030** (Scheme 1). Following the above terminology, we classified **032** as belonging to the “Instability Archipelago” (Figure 3). Furthermore, we have applied this classification to compounds from other structural group.

### 2.3. 6-Membered Heterocycles of C_4_H_5_NO

**Scheme 2:**
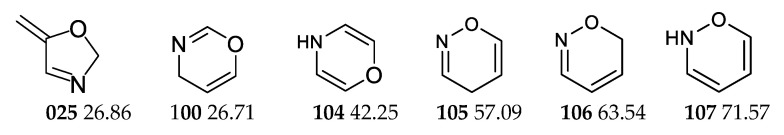
Compounds **025** and **100**, of almost equal stabilities, and compound **104**, which is 15–30 kcal/mol more stable than compounds **105–107** containing an N–O bond and their relative Gibbs free energies (kcal/mol).

Only eight 6-membered heterocycles were found (Table S1). They are significantly less stable than the 5-membered heterocycles. Thus, the most stable 6-membered heterocycle **100** is characterized by almost the same value of Gibbs free energy as the 26th most stable 5-membered heterocycle, **025** (Scheme 2). As in the previous case, the least stable compounds, **105–107**, containing N–O bonds were placed in the Instability Archipelago.

Formation of an Intermediate Containing an 1,2-Oxazine Ring **105** Has Been Proposed Based on the Structures of Its Decomposition Products [19].

### 2.4. 4-Membered Heterocycles of C_4_H_5_NO_2_

**Scheme 3:**
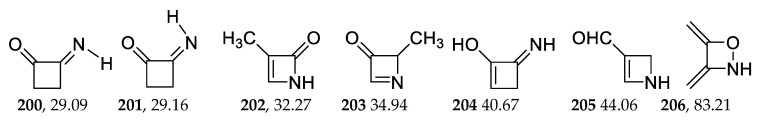
Four-membered cycle Stability Island (compounds **200–205**) and extremely unstable compound **206** and their relative Gibbs free energies (kcal/mol).

A compact Stability Island is observed, constituting six compounds, where several evident positional isomers and rotamers were neglected (Scheme 3 see also Table S3). The compound **206** is located about 40 kcal/mol away in the Instability Archipelago (Figure 3).

### 2.5. Saturated 3-Membered Heterocycles of C_4_H_5_NO

**Scheme 4:**
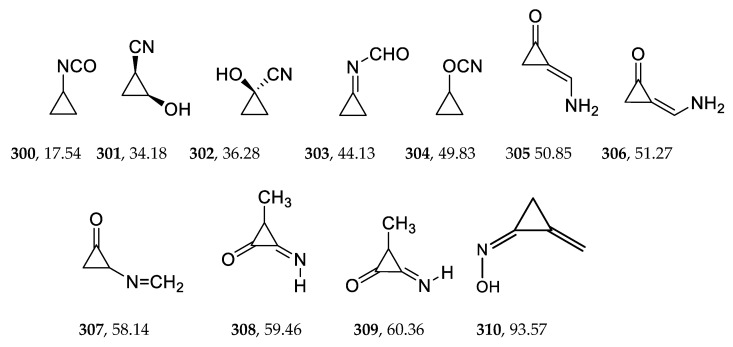
Members of the saturated three-membered cycles in the Stability Island and extremely unstable compound **310** in the Instability Archipelago and their relative Gibbs free energies (kcal/mol).

A relatively populated Stability Island was observed between the astonishingly stable isocyanate **300** and extremely unstable oxime **310** (NB—N-O bond, Scheme 4).

### 2.6. Unsaturated 3-Membered Heterocycles of C_4_H_5_NO

**Figure 4:**
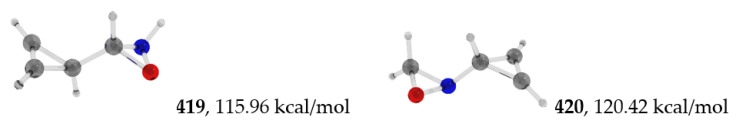
The least stable compounds found in the whole study and their relative Gibbs free energies (kcal/mol). Hydrogen: light grey, Carbon: black, Nitrogen: blue, Oxygen: red.

This Stability Island is not less populated than the 5-membered heterocycles Stability Island, and the potential for building further structures seems to be higher. Of note are the anti-records for the relative instability (compounds **419** and **420**, Figure 4) and for the difference between the most and least stable compounds in the series (85 kcal/mol).

### 2.7. Bicyclic Compounds of C_4_H_5_NO

**Scheme 5:**
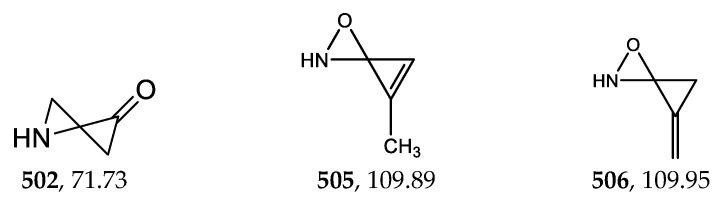
Members of the bicyclic Stability Island and their relative Gibbs free energies (kcal/mol).

These compounds have a somewhat expected limited population, thinly spread from 60 to 110 kcal/mol with significant potential for increasing towards the high energy side (Scheme 5).

### 2.8. Acyclic Nitriles

**Scheme 6:**
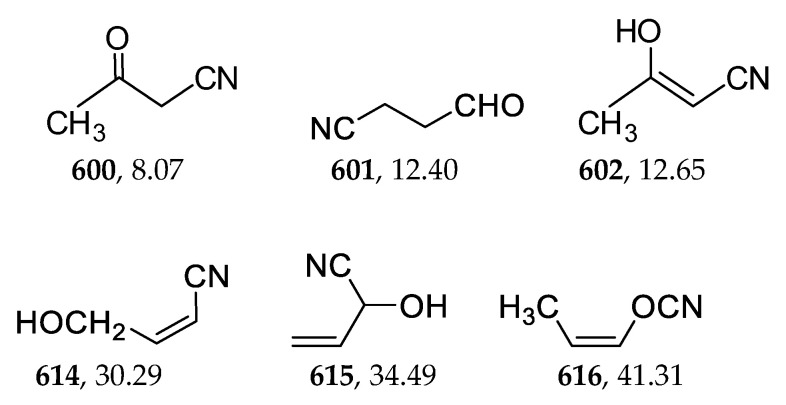
Members of the nitrile Stability Island and their relative Gibbs free energies (kcal/mol).

This group consists of numerous compounds inhabiting a compact Stability Island with an energy interval of 10–40 kcal/mol. Of interest is a very small gap between the most and the least stable compounds (Scheme 6), especially in view of compound **616** being well-known as a high energy material, which has also been confirmed computationally [20].

### 2.9. Trienes

**Scheme 7:**
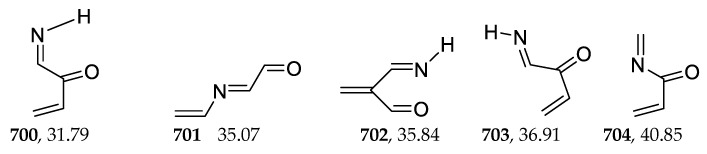
Members of the triene Stability Island and their relative Gibbs free energies (kcal/mol).

Only five compounds that can be formally considered as hetero-trienes were found in this study (Scheme 7). Comparing their relative stabilities, one can conclude that the N=CH_2_ moiety brings a considerable amount of instability into the molecule.

### 2.10. Acetylenes 

**Scheme 8:**
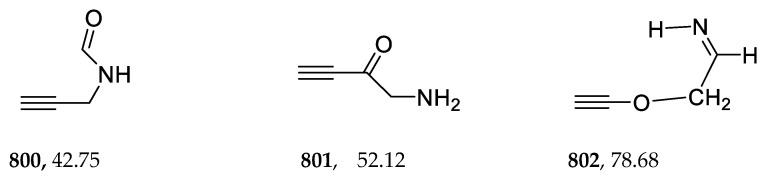
Members of the acetylene Stability Island and their relative Gibbs free energies (kcal/mol).

The least stable compound among the three containing a triple bond is an acetylenic ester **704** (Scheme 8). This is in accordance with the well-known high reactivity of acetylenic esters that makes them useful synthons for organic synthesis [21].

### 2.11. Allenes and Ketenes

**Scheme 9:**
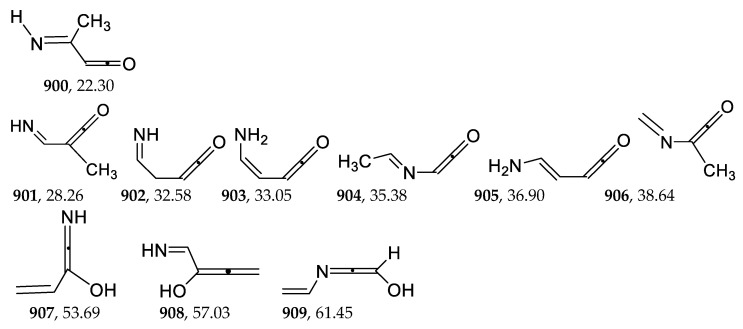
Members of the allene Stability Island and their relative Gibbs free energies (kcal/mol).

This class of located compounds is clearly divided into three groups: unexpectedly stable compound **900**, group of ketenes **901–906** with similar stabilities and allenic enoles **907–909,** which are 20–25 kcal/mol less stable (Scheme 9).

### 2.12. Isonitriles 

**Scheme 10:**
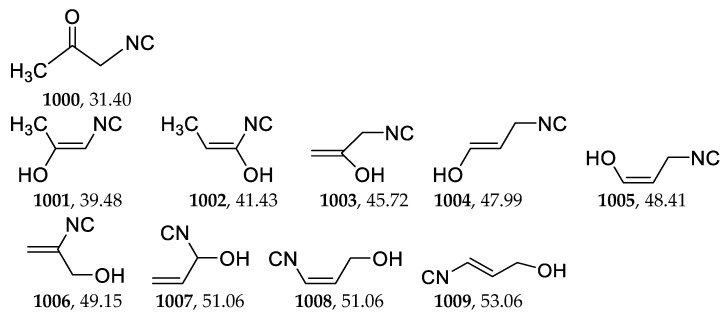
Members of the isonitrile Stability Island and their relative Gibbs free energies (kcal/mol).

The unusual electronic structure of isonitriles underlies their rich chemistry and numerous applications [22]. Most structures found in this study are hydroxy-substituted compounds, with the exception of the most stable ketone **1000** (Scheme 10). This explains the very close relative free energies of the compounds **1001–1009**.

### 2.13. Carbenes

**Scheme 11:**
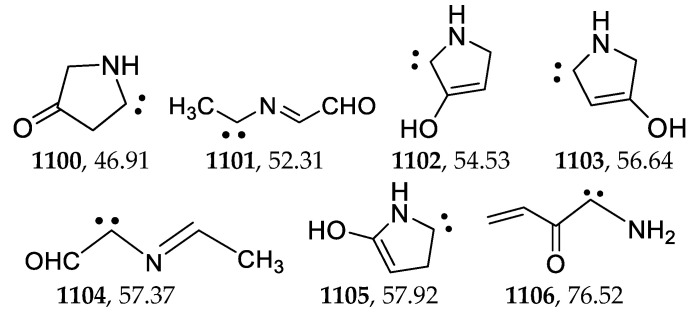
Carbene Stability Island (compounds **1100–1105**) and a member of the Instability Archipelago, compound **1106**, and their relative Gibbs free energies (kcal/mol).

Historically, carbenes were considered as extremely unstable species. This belief changed when the stabilizing effect of the adjacent nitrogen atom was recognized [23]. Accordingly, all located carbenes shown in Scheme 11 have a nitrogen atom in the α-position. Please note, however, that a carbene with an adjacent NH_2_ group is destabilized. All carbenes reported here are singlets.

### 2.14. Bipolar Compounds

**Figure 5:**
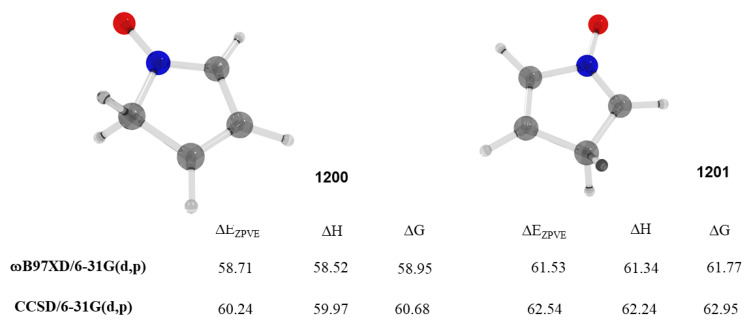
Optimized structures of the bipolar compounds **1200** and **1201** (CCSD/6-31G(d,p)), and their relative thermodynamic parameters (kcal/mol) in both levels of theory.

Two located bipolar compouynds are shown in Figure 5. They have very similar stabilitis, approximately 60 kcval/mol away from structurally similar Guiness molecules **000** and **001**. 

### 2.15. Molecular Associates

**Scheme 12:**
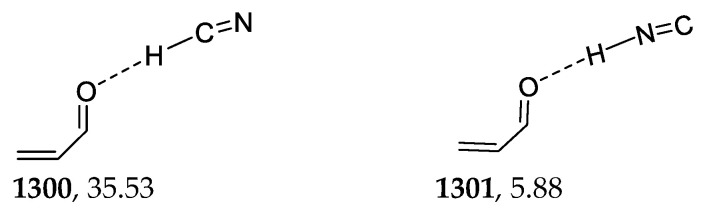
Molecular associates located in this study and their relative Gibbs free energies (kcal/mol).

Contrary to the study of carbohydrates [2], molecular associates of the formula C_4_H_5_NO are not very numerous. Only two examples, **1300** and **1301**, were found in this study (Scheme 12). This is probably due to the structural limitations stipulated by the chemical formula.

## 3. Conclusions

The main conclusion of our study is that we have gained valuable information, although this information has comes at a cost. Although the progress in computer performance and software development is fast, so far, only the accurate analysis of systems with a maximum of five heavy atoms (six in the case of HC_6_^+^) has been reported [11]. There must be a cheaper way to locate Guinness molecules, as at the moment, a complete energy mapping without gaps is hardly conceivable even for such relatively small molecules. Nevertheless, it seems that obtaining even approximate estimations on the energy gaps for certain molecule groups, such as those collected in Table 1, could be useful. Indeed, any interested chemist can easily construct a C_4_H_5_NO molecule not listed in this report. Optimization and frequency calculations for such a molecule take less than 5 minutes on a regular desktop computer (ωB97XD/6-31G(d,p)). Then, the data provided in our paper could help to estimate the relative energetics of this molecule compared to other similar compounds, and probably will give a rough idea of its reactivity.

We are considering further activities in these directions.

## Figures and Tables

**Table 1:** Thermodynamic parameters of C_4_H_5_NO molecules with the lowest and highest energy within the structural group.

Structure		E (Zpve), a.u.	H, a.u.	G (298), a.u.	ΔG (kcal/mol)
1. 5-membered heterocycles					
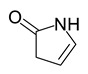	**000**	−285.229335	−285.22337	−285.257581	0.00
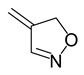	**032**	−285.142275	−285.136263	−285.170717	54.51
2. 6-membered heterocycles					
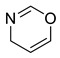	**100**	−285.186593	−285.180805	−285.215020	26.71
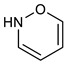	**107**	−285.115525	−285.109668	−285.143535	71.57
3. 4-membered heterocycles					
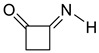	**200**	−285.182089	−285.175665	−285.211225	29.09
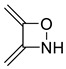	**206**	−285.090918	−285.089974	−285.124975	83.21
4. Saturated 3-membered cycles					
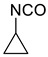	**300**	−285.199137	−285.192356	−285.229628	17.54
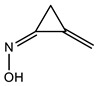	**310**	−285.079089	−285.072104	−285.108475	93.57
5. Unsaturated 3-membered cycles					
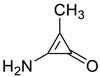	**400**	−285.167990	−285.159793	−285.202046	34.83
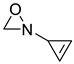	**420**	−285.036752	−285.030302	−285.065684	120.42
6. Bicyclic compounds					
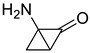	**500**	−285.149514	−285.142922	−285.178297	49.73
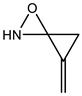	**506**	−285.053587	−285.047092	−285.082369	109.95
7. Acyclic nitriles					
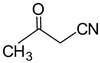	**600**	−285.215399	−285.208703	−285.244724	8.05
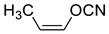	**617**	−285.161402	−285.154494	−285.191744	41.29
8. Conjugated trienes					
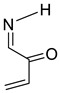	**700**	−285.177734	−285.171310	−285.206924	31.77
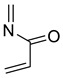	**704**	−285.162554	−285.155721	−285.192477	40.83
9. Acetylenes					
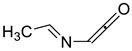	**800**	−285.170278	−285.162663	−285.201201	35.36
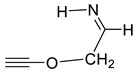	**802**	−285.101478	−285.093917	−285.132200	78.66
10. Allenes					
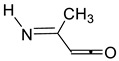	**900**	−285.191324	−285.183876	−285.222050	22.28
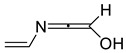	**906**	−285.128969	−285.121207	−285.159660	61.43
11. Acyclic isonitriles					
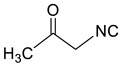	**1000**	−285.177682	−285.170656	−285.207548	31.38
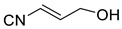	**1009**	−285.143396	−285.136367	−285.173026	53.04
12. Carbenes					
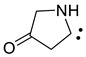	**1100**	−285.153903	−285.147722	−285.182830	46.89
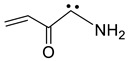	**1106**	−285.105616	−285.098696	−285.135632	76.50
13. Bipolar compounds					
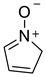	**1200**	−285.135776	−285.130117	−285.163639	58.52
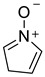	**1201**	−285.131281	−285.125617	−285.159143	61.75
14. Intermolecular associates					
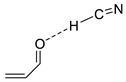	**1300**	−285.167303	−285.158443	−285.200968	35.53
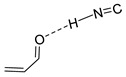	**1301**	−285.164966	−285.155864	−285.200403	46.78

## Data Availability

Not applicable.

## Supplementary Materials

The following supporting information can be downloaded at: https://www.mdpi.com/article/10.3390/molecules28020728/s1, Tables S1–S14 containing thermodynamic parameters and Cartesian coordinates for all located molecules of C_4_H_5_NO.
