# Supplementary material for: Global Search for Stable C4H5NO Compounds—Guinness Molecules and Stability Islands
Source: Molecules. 2023 Jan 11;28(2):728. doi: 10.3390/molecules28020728 (PMC9860754; doi:10.3390/molecules28020728)
Supplement: Supplementary file 1 [file molecules-28-00728-s001.zip › molecules-2081762-supplementary.pdf]

**Table S1. Molecules $C_4H_5NO$ containing 5-membered heterocycles and their thermodynamic parameters**

| Structure | E(zpve), a.u. | ΔE(zpve), kcal/mol | H, a.u. | ΔH(kcal/mol) | G(298), a.u. | ΔG(kcal/mol) |
|---|---|---|---|---|---|---|
| 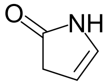 **000** | -285.229335 | 0 | -285.223367 | 0 | -285.257581 | 0 |
| 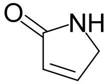 **001** | -285.229061 | 0.17 | -285.223032 | 0.21 | -285.257548 | 0.02 |
| 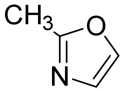 **002** | -285.220611 | 5.47 | -285.214513 | 5.56 | -285.249214 | 5.25 |
| 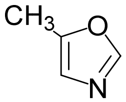 **003** | -285.218815 | 6.60 | -285.212765 | 6.65 | -285.247266 | 6.47 |
| 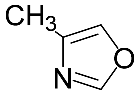 **004** | -285.217305 | 7.55 | -285.211248 | 7.60 | -285.245791 | 7.40 |
| 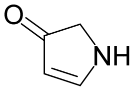 **005** | -285.213312 | 10.05 | -285.208309 | 9.45 | -285.241513 | 10.08 |
| 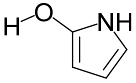 **006** | -285.212644 | 10.47 | -285.206465 | 10.61 | -285.240877 | 10.48 |
| 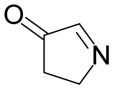 **007** | -285.209030 | 12.74 | -285.203052 | 12.75 | -285.237651 | 12.51 |
| 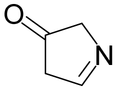 **008** | -285.208787 | 12.89 | -285.202817 | 12.90 | -285.237320 | 12.71 |
| 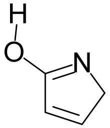 **009** | -285.209240 | 12.61 | -285.203516 | 12.46 | -285.237152 | 12.82 |

| | | | | | | |
|---|---|---|---|---|---|---|
| 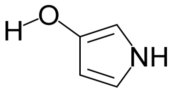 **010** | -285.208165 | 13.28 | -285.201927 | 13.45 | -285.236503 | 13.23 |
| 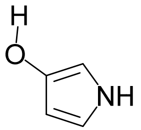 **011** | -285.207693 | 13.58 | -285.201520 | 13.71 | -285.235890 | 13.61 |
| 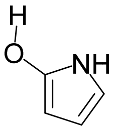 **012** | -285.206885 | 14.09 | -285.201149 | 13.94 | -285.234783 | 14.31 |
| 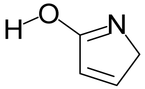 **013** | -285.200612 | 18.02 | -285.194710 | 17.98 | -285.228657 | 18.15 |
| 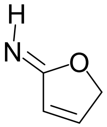 **014** | -285.200473 | 18.11 | -285.194736 | 17.97 | -285.228525 | 18.23 |
| 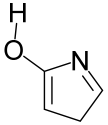 **015** | -285.198230 | 19.52 | -285.192314 | 19.49 | -285.226266 | 19.65 |
| 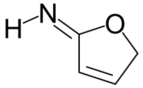 **016** | -285.195834 | 21.02 | -285.190090 | 20.88 | -285.223902 | 21.13 |
| 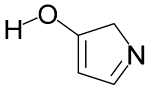 **017** | -285.195373 | 21.31 | -285.189527 | 21.23 | -285.223389 | 21.46 |
| 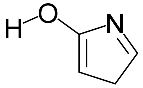 **018** | -285.193806 | 22.29 | -285.187914 | 22.25 | -285.221826 | 22.44 |
| 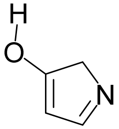 **019** | -285.193250 | 22.64 | -285.187290 | 22.64 | -285.221368 | 22.72 |

| | | | | | | |
|---|---|---|---|---|---|---|
| 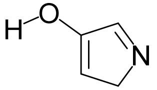 **020** | -285.192141 | 23.34 | -285.186252 | 23.29 | -285.220187 | 23.47 |
| 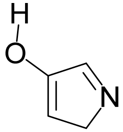 **021** | -285.190268 | 24.51 | -285.184214 | 24.57 | -285.218487 | 24.53 |
| 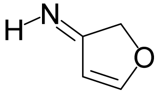 **022** | -285.188657 | 25.53 | -285.182835 | 25.43 | -285.216878 | 25.54 |
| 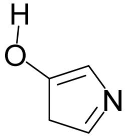 **023** | -285.188653 | 25.53 | -285.182706 | 25.52 | -285.216720 | 25.64 |
| 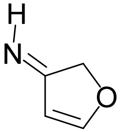 **024** | -285.186932 | 26.61 | -285.181113 | 26.51 | -285.215140 | 26.63 |
| 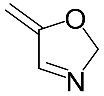 **025** | -285.181691 | 29.90 | -285.180747 | 26.74 | -285.214770 | 26.86 |
| 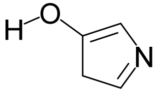 **026** | -285.185720 | 27.37 | -285.179546 | 27.50 | -285.214053 | 27.31 |
| 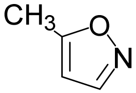 **027** | -285.183883 | 28.52 | -285.178466 | 28.18 | -285.211669 | 28.81 |
| 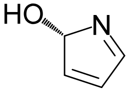 **028** | -285.182308 | 29.51 | -285.176323 | 29.52 | -285.210557 | 29.51 |
| 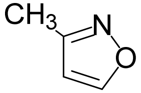 **029** | -285.180719 | 30.51 | -285.175338 | 30.14 | -285.208469 | 30.82 |

| | | | | | | |
|---|---|---|---|---|---|---|
| 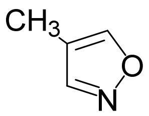 **030** | -285.178201 | 32.09 | -285.172060 | 32.20 | -285.206918 | 31.79 |
| 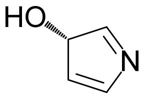 **031** | -285.173373 | 35.12 | -285.167368 | 35.14 | -285.201652 | 35.10 |
| 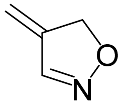 **032** | -285.142275 | 54.63 | -285.136263 | 54.66 | -285.170717 | 54.51 |

**Table S2. Molecules $C_4H_5NO$ containing 6-membered heterocycles and their thermodynamic parameters**

| Structure | E(zpve). a.u. | ΔE(zpve), kcal/mol | H, a.u. | ΔH(kcal/mol) | G(298). a.u. | ΔG(kcal/mol) |
|---|---|---|---|---|---|---|
| 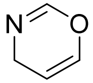 **100** | -285.186593 | 26.82 | -285.180805 | 26.71 | -285.215020 | 26.71 |
| 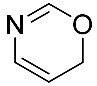 **101** | -285.185214 | 27.69 | -285.179395 | 27.59 | -285.213585 | 27.61 |
| 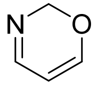 **102** | -285.181749 | 29.86 | -285.176103 | 29.66 | -285.209692 | 30.05 |
| 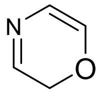 **103** | -285.173018 | 35.34 | -285.167415 | 35.11 | -285.200858 | 35.59 |
| 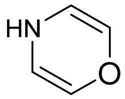 **104** | -285.161914 | 42.31 | -285.156031 | 42.25 | -285.190256 | 42.25 |
| 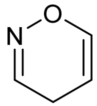 **105** | -285.137756 | 57.47 | -285.131843 | 57.43 | -285.166604 | 57.09 |

| | | | | | | |
|---|---|---|---|---|---|---|
| 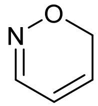 **106** | -285.128733 | 63.13 | -285.123531 | 62.65 | -285.156322 | 63.54 |
| 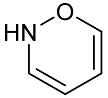 **107** | -285.115525 | 71.42 | -285.109668 | 71.35 | -285.143535 | 71.57 |

**Table S3. Molecules $C_4H_5NO$ containing 4-membered heterocycles and their thermodynamic parameters**

| Structure | E(zpve). a.u. | ΔE(zpve), kcal/mol | H, a.u. | ΔH(kcal/mol) | G(298). a.u. | ΔG(kcal/mol) |
|---|---|---|---|---|---|---|
| 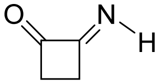 **200** | -285.182089 | 29.65 | -285.175665 | 29.93 | -285.211225 | 29.09 |
| 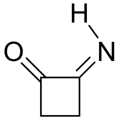 **201** | -285.181981 | 29.72 | -285.175566 | 30.00 | -285.211083 | 29.18 |
| 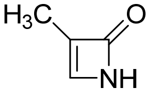 **202** [a] | -285.177060 | 32.80 | -285.170390 | 33.24 | -285.206128 | 32.29 |
| 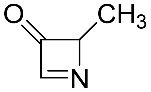 **203** [a] | -285.172826 | 35.46 | -285.166249 | 35.84 | -285.201867 | 34.96 |
| 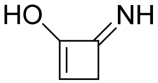 **204** [a.b] | -285.164083 | 40.95 | -285.157723 | 41.19 | -285.192740 | 40.69 |
| 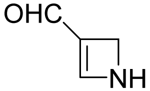 **205** [a] | -285.158320 | 44.56 | -285.151832 | 44.89 | -285.187334 | 44.08 |
| 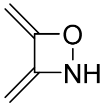 **206** [a] | -285.090918 | 86.86 | -285.089974 | 83.71 | -285.124975 | 83.21 |

[a]Positional isomer neglected; [b]rotamer neglected.

**Table S4. Molecules $C_4H_5NO$ containing saturated 3-membered cycles and their thermodynamic parameters.**

| Structure | E(zpve). a.u. | ΔE(zpve), kcal/mol | H, a.u. | ΔH(kcal/mol) | G(298). a.u. | ΔG(kcal/mol) |
|---|---|---|---|---|---|---|
| 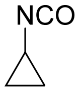 **300** | -285.199137 | 18.95 | -285.192356 | 19.46 | -285.229628 | 17.54 |
| 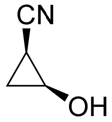 **301**[a] | -285.173826 | 34.83 | -285.167011 | 35.36 | -285.203084 | 34.20 |
| 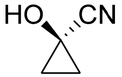 **302** | -285.170962 | 36.63 | -285.164610 | 36.87 | -285.199725 | 36.31 |
| 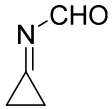 **303**[b] | -285.157401 | 45.14 | -285.150456 | 45.75 | -285.187228 | 44.15 |
| 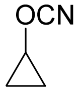 **304** | -285.149779 | 49.92 | -285.143849 | 49.90 | -285.178174 | 49.83 |
| 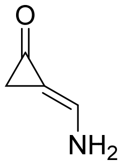 **305** | -285.146515 | 51.97 | -285.139109 | 52.87 | -285.176552 | 50.85 |
| 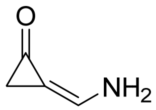 **306** | -285.145812 | 52.41 | -285.138411 | 53.31 | -285.175883 | 51.27 |

| | | | | | | |
|---|---|---|---|---|---|---|
| 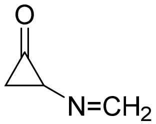 **307**[b] | -285.135249 | 59.04 | -285.128359 | 59.62 | -285.164931 | 58.14 |
| 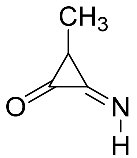 **308** | -285.132579 | 60.72 | -285.125136 | 61.64 | -285.162826 | 59.46 |
| 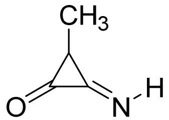 **309** | -285.131205 | 61.58 | -285.123797 | 62.48 | -285.161392 | 60.36 |
| 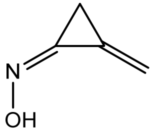 **310** | -285.079089 | 94.28 | -285.072104 | 94.92 | -285.108475 | 93.57 |

[a]Positional isomer neglected; [b]rotamer neglected.

**Table S5. Molecules $C_4H_5NO$ containing unsaturated 3-membered cycles and their thermodynamic parameters**

| Structure | E(zpve). a.u. | ΔE(zpve), kcal/mol | H, a.u. | ΔH(kcal/mol) | G(298). a.u. | ΔG(kcal/mol) |
|---|---|---|---|---|---|---|
| 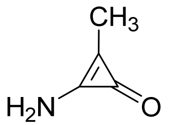 **400** | -285.167990 | 38.49 | -285.159793 | 39.89 | -285.202046 | 34.85 |
| 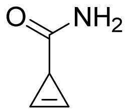 **401** | -285.157527 | 45.06 | -285.150189 | 45.92 | -285.187639 | 43.89 |

| | | | | | | |
|---|---|---|---|---|---|---|
| 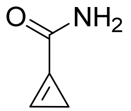 **402** [b] | -285.154099 | 47.21 | -285.146418 | 48.29 | -285.185658 | 45.13 |
| 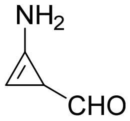 **403** [a] | -285.136616 | 58.18 | -285.129322 | 59.01 | -285.166376 | 57.23 |
| 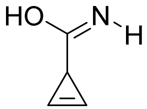 **404** | -285.135031 | 59.18 | -285.128290 | 59.66 | -285.164466 | 58.43 |
| 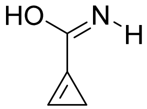 **405** | -285.134187 | 59.71 | -285.127284 | 60.29 | -285.163876 | 58.80 |
| 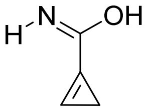 **406** | -285.132766 | 60.60 | -285.125837 | 61.20 | -285.162779 | 59.49 |
| 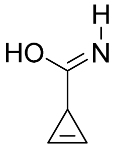 **407** | -285.129321 | 62.76 | -285.122511 | 63.29 | -285.158725 | 62.03 |
| 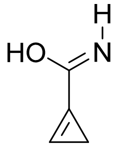 **408** | -285.127080 | 64.17 | -285.120095 | 64.80 | -285.156831 | 63.22 |
| 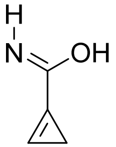 **409** | -285.126148 | 64.75 | -285.119158 | 65.39 | -285.156083 | 63.69 |
| 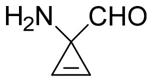 **410** [a] | -285.122136 | 67.27 | -285.114737 | 68.17 | -285.152455 | 65.97 |

| | | | | | | |
|---|---|---|---|---|---|---|
| 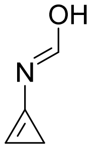 **411** | -285.118767 | 69.38 | -285.111768 | 70.03 | -285.148270 | 68.59 |
| 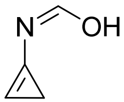 **412** | -285.116870 | 70.57 | -285.109797 | 71.27 | -285.147091 | 69.33 |
| 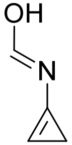 **413** | -285.115440 | 71.47 | -285.108310 | 72.20 | -285.145278 | 70.47 |
| 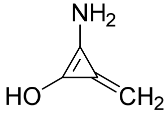 **414** | -285.112647 | 73.22 | -285.105165 | 74.17 | -285.142344 | 72.31 |
| 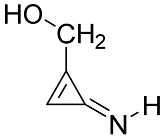 **415** | -285.109901 | 74.95 | -285.102549 | 75.81 | -285.140685 | 73.35 |
| 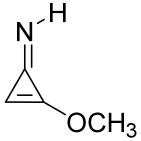 **416** [b] | -285.108441 | 75.86 | -285.101229 | 76.64 | -285.138455 | 74.75 |
| 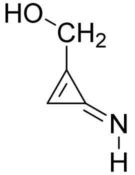 **417** | -285.108110 | 76.07 | -285.101596 | 76.41 | -285.137277 | 75.49 |
| 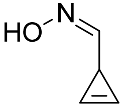 **418** | -285.070976 | 99.37 | -285.064005 | 100.00 | -285.100643 | 98.48 |

| | | | | | | |
|---|---|---|---|---|---|---|
| 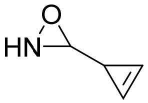 **419** | -285.043661 | 116.51 | -285.037177 | 116.84 | -285.072792 | 115.96 |
| 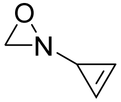 **420** | -285.036752 | 120.85 | -285.030302 | 121.15 | -285.065684 | 120.42 |

[a]Positional isomer neglected; [b]rotamer neglected.

**Table S6. Bicyclic compounds $C_4H_5NO$ and their thermodynamic parameters**

| Structure | E(zpve). a.u. | ΔE(zpve), kcal/mol | H, a.u. | ΔH(kcal/mol) | G(298). a.u. | ΔG(kcal/mol) |
|---|---|---|---|---|---|---|
| 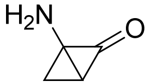 **500** [a] | -285.149514 | 50.09 | -285.142922 | 50.48 | -285.178297 | 49.75 |
| 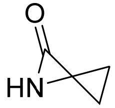 **501** | -285.138734 | 56.85 | -285.132420 | 57.07 | -285.167353 | 56.62 |
| 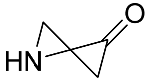 **502** [a] | -285.114446 | 72.09 | -285.108009 | 72.39 | -285.143278 | 71.73 |
| 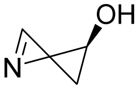 **503** [a] | -285.096723 | 83.22 | -285.090108 | 83.62 | -285.125507 | 82.88 |
| 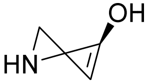 **504** [a] | -285.078719 | 94.51 | -285.072219 | 94.85 | -285.107410 | 94.23 |
| 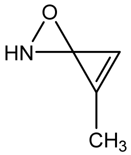 **505** | -285.053056 | 110.62 | -285.046161 | 111.20 | -285.082466 | 109.89 |

| | | | | | | |
|---|---|---|---|---|---|---|
| 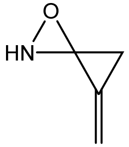 **506** | -285.053587 | 110.28 | -285.047092 | 110.61 | -285.082369 | 109.95 |

[a]Positional isomer neglected.

**Table S7. Acyclic nitriles $C_4H_5NO$ and their thermodynamic parameters**

| Structure | E(zpve). a.u. | ΔE(zpve), kcal/mol | H, a.u. | ΔH(kcal/mol) | G(298). a.u. | ΔG(kcal/mol) |
|---|---|---|---|---|---|---|
| 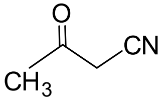 **600** | -285.215399 | 8.74 | -285.208703 | 9.20 | -285.244724 | 8.07 |
| 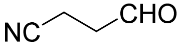 **601** | -285.207256 | 13.85 | -285.199916 | 14.72 | -285.237819 | 12.40 |
| 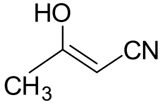 **602** | -285.202790 | 16.66 | -285.195400 | 17.55 | -285.232649 | 15.65 |
| 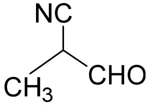 **603** | -285.200916 | 17.83 | -285.193452 | 18.77 | -285.231316 | 16.48 |
| 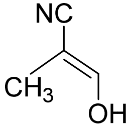 **604** | -285.196166 | 20.81 | -285.188543 | 21.85 | -285.226483 | 19.51 |
| 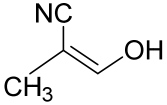 **605** | -285.193626 | 22.41 | -285.186073 | 23.40 | -285.223717 | 21.25 |
| 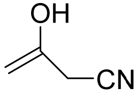 **606** | -285.190455 | 24.40 | -285.183266 | 25.16 | -285.220556 | 23.23 |

| | | | | | | |
|---|---|---|---|---|---|---|
| 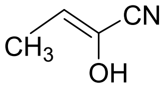 **607** | -285.189308 | 25.12 | -285.181550 | 26.24 | -285.220307 | 23.39 |
| 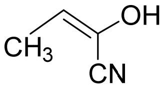 **608** | -285.189493 | 25.00 | -285.181983 | 25.97 | -285.219521 | 23.88 |
| 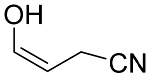 **609** | -285.186123 | 27.12 | -285.179431 | 27.57 | -285.215378 | 26.48 |
| 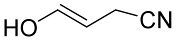 **610** | -285.184869 | 27.90 | -285.177337 | 28.88 | -285.215382 | 26.48 |
| 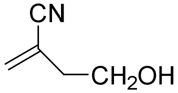 **611** | -285.179658 | 31.17 | -285.172261 | 32.07 | -285.210008 | 29.85 |
| 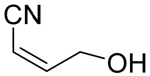 **612** | -285.179824 | 31.07 | -285.173129 | 31.52 | -285.209299 | 30.30 |
| 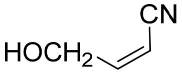 **613** | -285.179809 | 31.08 | -285.173124 | 31.53 | -285.209275 | 30.31 |
| 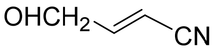 **614** | -285.179809 | 31.08 | -285.171031 | 32.84 | -285.207475 | 31.44 |
| 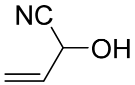 **615** | -285.172284 | 35.80 | -285.164814 | 36.74 | -285.202590 | 34.51 |
| 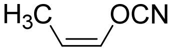 **616** | -285.161402 | 42.63 | -285.154494 | 43.22 | -285.191744 | 41.31 |

**Table S8. Conjugated trienes $C_4H_5NO$ and their thermodynamic parameters**

| Structure | E(zpve). a.u. | ΔE(zpve), kcal/mol | H, a.u. | ΔH(kcal/mol) | G(298). a.u. | ΔG(kcal/mol) |
|---|---|---|---|---|---|---|
| 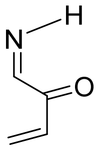 **700** [b] | -285.177734 | 32.38 | -285.171310 | 32.67 | -285.206924 | 31.79 |

| | | | | | | |
|---|---|---|---|---|---|---|
| 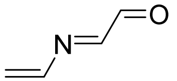 **701** [b] | -285.171676 | 36.18 | -285.164393 | 37.01 | -285.201694 | 35.07 |
| 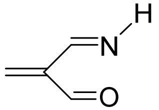 **702** [b] | -285.171283 | 36.43 | -285.164836 | 36.73 | -285.200461 | 35.84 |
| 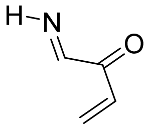 **703** [b] | -285.168799 | 37.99 | -285.162215 | 38.37 | -285.198755 | 36.91 |
| 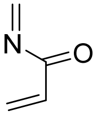 **704** [b] | -285.162554 | 41.91 | -285.155721 | 42.45 | -285.192477 | 40.85 |

[b]Rotamer neglected.

**Table S9. Acetylenes $C_4H_5NO$ and their thermodynamic parameters**

| Structure | E(zpve). a.u. | ΔE(zpve), kcal/mol | H, a.u. | ΔH(kcal/mol) | G(298). a.u. | ΔG(kcal/mol) |
|---|---|---|---|---|---|---|
| 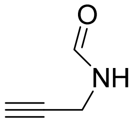 **800** | -285.158760 | 44.29 | -285.151329 | 45.20 | -285.189458 | 42.75 |
| 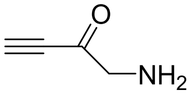 **801** | -285.144009 | 53.54 | -285.136338 | 54.61 | -285.174522 | 52.12 |
| 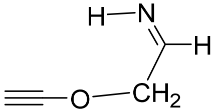 **802** [b] | -285.101478 | 80.23 | -285.093917 | 81.23 | -285.132200 | 78.68 |

[b]Rotamer neglected.

**Table S10. Allenes $C_4H_5NO$. ketenes and their thermodynamic parameters**

| Structure | E(zpve). a.u. | ΔE(zpve), kcal/mol | H, a.u. | ΔH(kcal/mol) | G(298). a.u. | ΔG(kcal/mol) |
|---|---|---|---|---|---|---|
| 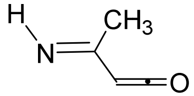 **900** [b] | -285.191324 | 23.85 | -285.183876 | 24.78 | -285.222050 | 22.30 |
| 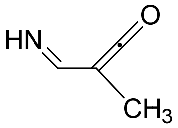 **901** [b] | -285.182745 | 29.24 | -285.175883 | 29.80 | -285.212549 | 28.26 |
| 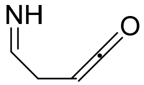 **902** [b] | -285.174717 | 34.27 | -285.167318 | 35.17 | -285.205666 | 32.58 |
| 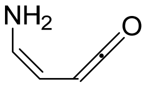 **903** [b] | -285.175228 | 33.95 | -285.168409 | 34.49 | -285.204908 | 33.05 |
| 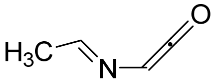 **904** [b] | -285.170278 | 37.06 | -285.162663 | 38.09 | -285.201201 | 35.38 |
| 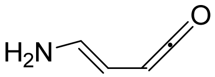 **905** [b] | -285.167770 | 38.63 | -285.160601 | 39.39 | -285.198776 | 36.90 |
| 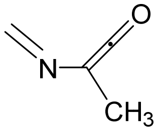 **906** [b] | -285.164811 | 40.49 | -285.157007 | 41.64 | -285.195997 | 38.64 |
| 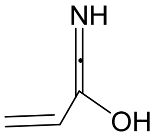 **907** [b] | -285.142065 | 54.76 | -285.134612 | 55.69 | -285.172017 | 53.69 |
| 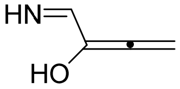 **908** [b] | -285.136723 | 58.11 | -285.129354 | 58.99 | -285.166697 | 57.03 |

| | | | | | | |
|---|---|---|---|---|---|---|
| 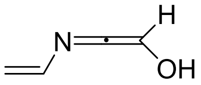 **909** [b] | -285.128969 | 62.98 | -285.121207 | 64.11 | -285.159660 | 61.45 |

[b]Rotamer neglected.

**Table S11. Acyclic isonitriles $C_4H_5NO$ and their thermodynamic parameters**

| Structure | E(zpve). a.u. | ΔE(zpve), kcal/mol | H, a.u. | ΔH(kcal/mol) | G(298). a.u. | ΔG(kcal/mol) |
|---|---|---|---|---|---|---|
| 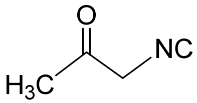 **1000** | -285.177682 | 32.41 | -285.170656 | 33.08 | -285.207548 | 31.40 |
| 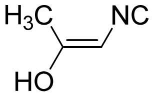 **1001** | -285.164412 | 40.74 | -285.156678 | 41.85 | -285.194667 | 39.48 |
| 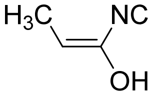 **1002** | -285.161051 | 42.85 | -285.153108 | 44.09 | -285.191707 | 41.34 |
| 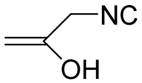 **1003** | -285.154690 | 46.84 | -285.147310 | 47.73 | -285.184717 | 45.72 |
| 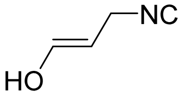 **1004** | -285.150526 | 49.45 | -285.142845 | 50.53 | -285.181105 | 47.99 |
| 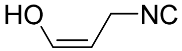 **1005** | -285.103766 | 78.80 | -285.144141 | 49.72 | -285.180427 | 48.41 |
| 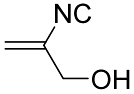 **1006** | -285.149063 | 50.37 | -285.141604 | 51.31 | -285.179255 | 49.15 |
| 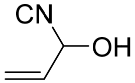 **1007** | -285.145973 | 52.31 | -285.138446 | 53.29 | -285.176215 | 51.06 |
| | | | | | | |

| | | | | | | |
|---|---|---|---|---|---|---|
| 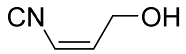 **1008** | -285.146626 | 51.90 | -285.139782 | 52.45 | -285.176204 | 51.06 |
| 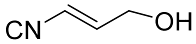 **1009** | -285.143396 | 53.93 | -285.136367 | 54.59 | -285.173026 | 53.06 |

**Table S12. Carbenes $C_4H_5NO$ and their thermodynamic parameters**

| Structure | E(zpve). a.u. | ΔE(zpve), kcal/mol | H, a.u. | ΔH(kcal/mol) | G(298). a.u. | ΔG(kcal/mol) |
|---|---|---|---|---|---|---|
| 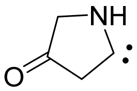 **1100** [a] | -285.153903 | 47.33 | -285.147722 | 47.47 | -285.182830 | 46.91 |
| 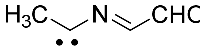 **1101** [a.b] | -285.143530 | 53.84 | -285.135770 | 54.97 | -285.174218 | 52.31 |
| 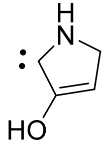 **1102** | -285.142635 | 54.41 | -285.136711 | 54.38 | -285.170686 | 54.53 |
| 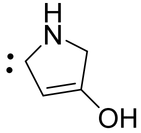 **1103** | -285.139204 | 56.56 | -285.133209 | 56.58 | -285.167312 | 56.64 |
| 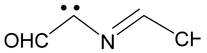 **1104** | -285.135128 | 59.12 | -285.127340 | 60.26 | -285.166156 | 57.37 |
| 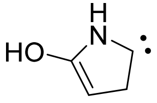 **1105** [a] | -285.137037 | 57.92 | -285.130872 | 58.04 | -285.165280 | 57.92 |
| 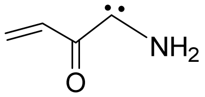 **1106** [a.b] | -285.105616 | 77.63 | -285.098696 | 78.23 | -285.135632 | 76.52 |

[a]Positional isomer neglected; [b]rotamer neglected.

## Table S13. Bipolar compounds $C_4H_5NO$ and their thermodynamic parameters

| Structure | E(zpve). a.u. | ΔE(zpve), kcal/mol | H, a.u. | ΔH(kcal/mol) | G(298). a.u. | ΔG(kcal/mol) |
|---|---|---|---|---|---|---|
| 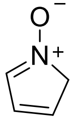 **1200** [a.b] | -285.135776 | 58.71 | -285.130117 | 58.52 | -285.163639 | 58.95 |
| 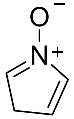 **1201** [a.b] | -285.131281 | 61.53 | -285.125617 | 61.34 | -285.159143 | 61.77 |

[a]Positional isomer neglected; [b]rotamer neglected.

## Table S14. Intermolecular associates $C_4H_5NO$ and their thermodynamic parameters

| Structure | E(zpve). a.u. | ΔE(zpve), kcal/mol | H, a.u. | ΔH(kcal/mol) | G(298). a.u. | ΔG(kcal/mol) |
|---|---|---|---|---|---|---|
| 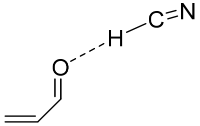 **1300** [b] | -285.167303 | 38.93 | -285.158443 | 40.74 | -285.200968 | 35.53 |
| 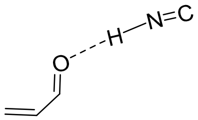 **1301** [b] | -285.164966 | 40.39 | -285.155864 | 42.36 | -285.200403 | 35.88 |

[b]Rotamer neglected.

**000**

Standard orientation:

| Center Number | Atomic Number | Atomic Type | Coordinates (Angstroms) X | Y | Z |
|---|---|---|---|---|---|
| 1 | 6 | 0 | 1.333766 | -0.745002 | -0.000015 |
| 2 | 6 | 0 | 1.482851 | 0.582328 | -0.000180 |
| 3 | 6 | 0 | 0.117755 | 1.212895 | 0.000110 |
| 4 | 1 | 0 | 2.089388 | -1.518311 | -0.000113 |
| 5 | 1 | 0 | 2.421929 | 1.114992 | 0.000177 |
| 6 | 1 | 0 | -0.082120 | 1.833387 | -0.880104 |
| 7 | 1 | 0 | -0.081698 | 1.833554 | 0.880273 |
| 8 | 7 | 0 | -0.022118 | -1.101158 | 0.000052 |
| 9 | 1 | 0 | -0.388399 | -2.038084 | 0.000325 |
| 10 | 6 | 0 | -0.837058 | 0.014504 | 0.000052 |
| 11 | 8 | 0 | -2.048520 | 0.011777 | -0.000091 |

**001**

Standard orientation:

| Center Number | Atomic Number | Atomic Type | Coordinates (Angstroms) X | Y | Z |
|---|---|---|---|---|---|
| 1 | 6 | 0 | -0.858335 | 0.022595 | -0.000039 |
| 2 | 6 | 0 | 0.048654 | 1.211645 | 0.000027 |
| 3 | 6 | 0 | 1.316366 | 0.802890 | 0.000141 |
| 4 | 6 | 0 | 1.382013 | -0.701431 | -0.000037 |
| 5 | 1 | 0 | -0.327802 | 2.224930 | -0.000616 |
| 6 | 1 | 0 | 2.205963 | 1.420024 | -0.000171 |
| 7 | 1 | 0 | 1.910128 | -1.077064 | -0.886648 |
| 8 | 1 | 0 | 1.910558 | -1.077221 | 0.886365 |
| 9 | 8 | 0 | -2.072644 | -0.007890 | 0.000053 |
| 10 | 7 | 0 | -0.012578 | -1.062396 | 0.000093 |
| 11 | 1 | 0 | -0.361838 | -2.004974 | -0.000557 |

**002**

Standard orientation:

| Center Number | Atomic Number | Atomic Type | Coordinates (Angstroms) X | Y | Z |
|---|---|---|---|---|---|
| 1 | 6 | 0 | -0.596951 | 0.095813 | -0.000008 |
| 2 | 6 | 0 | 1.443174 | 0.673303 | 0.000018 |
| 3 | 7 | 0 | 0.142707 | 1.158157 | 0.000008 |
| 4 | 1 | 0 | 2.297543 | 1.332589 | -0.000056 |
| 5 | 6 | 0 | -2.079530 | 0.001959 | 0.000044 |
| 6 | 1 | 0 | -2.493566 | 1.009674 | 0.000228 |
| 7 | 1 | 0 | -2.435908 | -0.533144 | -0.884491 |
| 8 | 1 | 0 | -2.435893 | -0.533811 | 0.884096 |
| 9 | 6 | 0 | 1.412788 | -0.676177 | -0.000015 |
| 10 | 1 | 0 | 2.152825 | -1.458451 | 0.000103 |
| 11 | 8 | 0 | 0.104895 | -1.061668 | -0.000021 |

**003**

Standard orientation:

---------------------------------------------------------------------

Center Atomic Atomic Coordinates (Angstroms)

| Number | Number | Type | X | Y | Z |
|---|---|---|---|---|---|
| 1 | 6 | 0 | -1.400254 | -0.631564 | -0.000033 |
| 2 | 6 | 0 | -0.244539 | 1.130242 | -0.000068 |
| 3 | 7 | 0 | -1.547201 | 0.649041 | -0.000106 |
| 4 | 1 | 0 | -0.027440 | 2.187739 | -0.000114 |
| 5 | 6 | 0 | 0.625736 | 0.092967 | 0.000021 |
| 6 | 8 | 0 | -0.118633 | -1.054157 | 0.000038 |
| 7 | 1 | 0 | -2.164057 | -1.394164 | -0.000034 |
| 8 | 6 | 0 | 2.103528 | -0.040332 | 0.000110 |
| 9 | 1 | 0 | 2.452005 | -0.581210 | 0.885036 |
| 10 | 1 | 0 | 2.452097 | -0.581371 | -0.884680 |
| 11 | 1 | 0 | 2.560039 | 0.951090 | 0.000045 |

**004**

Standard orientation:

| Center Number | Atomic Number | Atomic Type | Coordinates (Angstroms) X | Y | Z |
|---|---|---|---|---|---|
| 1 | 6 | 0 | -1.349363 | -0.702304 | -0.000051 |
| 2 | 6 | 0 | 0.643680 | 0.017320 | 0.000025 |
| 3 | 7 | 0 | -0.134954 | -1.137597 | 0.000083 |
| 4 | 6 | 0 | -0.183460 | 1.088648 | 0.000080 |
| 5 | 8 | 0 | -1.471688 | 0.636937 | -0.000042 |
| 6 | 1 | 0 | -2.264125 | -1.275496 | -0.000077 |
| 7 | 6 | 0 | 2.133307 | -0.040979 | -0.000042 |
| 8 | 1 | 0 | 2.496313 | -0.575547 | -0.882319 |
| 9 | 1 | 0 | 2.565174 | 0.962358 | 0.000184 |
| 10 | 1 | 0 | 2.496438 | -0.575991 | 0.881896 |
| 11 | 1 | 0 | -0.040593 | 2.156250 | -0.000001 |

**005**

Standard orientation:

| Center Number | Atomic Number | Atomic Type | Coordinates (Angstroms) X | Y | Z |
|---|---|---|---|---|---|
| 1 | 6 | 0 | -0.868865 | 0.056411 | 0.006507 |
| 2 | 6 | 0 | 0.011047 | 1.219074 | 0.014593 |
| 3 | 6 | 0 | 1.289438 | 0.769444 | 0.002358 |
| 4 | 1 | 0 | -0.312406 | 2.248257 | 0.012782 |
| 5 | 1 | 0 | 2.195272 | 1.364266 | 0.031867 |
| 6 | 8 | 0 | -2.081683 | -0.011281 | -0.008768 |
| 7 | 7 | 0 | 1.397710 | -0.591648 | -0.092282 |
| 8 | 1 | 0 | 2.202339 | -1.065111 | 0.283499 |
| 9 | 6 | 0 | 0.069765 | -1.170838 | 0.018305 |
| 10 | 1 | 0 | -0.162218 | -1.836565 | -0.817440 |
| 11 | 1 | 0 | -0.061804 | -1.723607 | 0.954831 |

**006**

Standard orientation:

| Center Number | Atomic Number | Atomic Type | Coordinates (Angstroms) X | Y | Z |
|---|---|---|---|---|---|
| 1 | 6 | 0 | -0.685832 | 0.063888 | -0.000095 |
| 2 | 6 | 0 | 0.124916 | 1.171544 | -0.000081 |

| | | | | | |
|---|---|---|---|---|---|
| 3 | 6 | 0 | 1.468297 | 0.682297 | 0.000074 |
| 4 | 6 | 0 | 1.420511 | -0.684394 | 0.000019 |
| 5 | 1 | 0 | -0.198590 | 2.202211 | -0.000017 |
| 6 | 1 | 0 | 2.366206 | 1.282548 | 0.000144 |
| 7 | 1 | 0 | 2.202670 | -1.426489 | -0.000316 |
| 8 | 8 | 0 | -2.030590 | -0.105906 | -0.000089 |
| 9 | 7 | 0 | 0.088947 | -1.054559 | 0.000015 |
| 10 | 1 | 0 | -0.275234 | -1.991391 | 0.000424 |
| 11 | 1 | 0 | -2.440314 | 0.762271 | 0.000869 |

**007**

Standard orientation:

| Center Number | Atomic Number | Atomic Type | Coordinates (Angstroms) X | Y | Z |
|---|---|---|---|---|---|
| 1 | 6 | 0 | -0.872377 | -0.016997 | -0.000025 |
| 2 | 6 | 0 | 0.087133 | -1.200856 | -0.000048 |
| 3 | 6 | 0 | 1.465562 | -0.527535 | 0.000084 |
| 4 | 1 | 0 | -0.097536 | -1.820410 | 0.881263 |
| 5 | 1 | 0 | 2.067827 | -0.780009 | -0.877463 |
| 6 | 8 | 0 | -2.078459 | -0.027922 | -0.000048 |
| 7 | 7 | 0 | 1.264371 | 0.931685 | 0.000043 |
| 8 | 6 | 0 | 0.019558 | 1.193851 | -0.000008 |
| 9 | 1 | 0 | -0.362662 | 2.211414 | -0.000032 |
| 10 | 1 | 0 | 2.067607 | -0.779959 | 0.877799 |
| 11 | 1 | 0 | -0.097423 | -1.820235 | -0.881509 |

**008**

Standard orientation:

| Center Number | Atomic Number | Atomic Type | Coordinates (Angstroms) X | Y | Z |
|---|---|---|---|---|---|
| 1 | 6 | 0 | 0.847778 | 0.019040 | -0.000112 |
| 2 | 6 | 0 | -0.080633 | 1.232501 | -0.000062 |
| 3 | 6 | 0 | -1.427700 | 0.561856 | 0.000208 |
| 4 | 1 | 0 | 0.090719 | 1.860745 | 0.880761 |
| 5 | 1 | 0 | -2.363948 | 1.118028 | 0.000443 |
| 6 | 8 | 0 | 2.052392 | 0.021500 | 0.000052 |
| 7 | 7 | 0 | -1.441654 | -0.712825 | -0.000186 |
| 8 | 6 | 0 | -0.069075 | -1.206134 | 0.000045 |
| 9 | 1 | 0 | 0.116594 | -1.832812 | -0.879171 |
| 10 | 1 | 0 | 0.090125 | 1.860321 | -0.881412 |
| 11 | 1 | 0 | 0.116722 | -1.832085 | 0.879794 |

**009**

Standard orientation:

| Center Number | Atomic Number | Atomic Type | Coordinates (Angstroms) X | Y | Z |
|---|---|---|---|---|---|
| 1 | 6 | 0 | -1.411011 | 0.726901 | -0.000174 |
| 2 | 6 | 0 | -0.153826 | 1.188056 | 0.000158 |
| 3 | 6 | 0 | 0.695446 | -0.009028 | -0.000487 |
| 4 | 7 | 0 | 0.066831 | -1.128064 | 0.000125 |
| 5 | 6 | 0 | -1.343167 | -0.775258 | 0.000031 |
| 6 | 1 | 0 | -1.841525 | -1.202420 | 0.879904 |

| | | | | | |
|---|---|---|---|---|---|
| 7 | 1 | 0 | 0.201621 | 2.209192 | 0.000232 |
| 8 | 1 | 0 | -1.841804 | -1.203708 | -0.878961 |
| 9 | 8 | 0 | 2.029623 | 0.100448 | 0.000180 |
| 10 | 1 | 0 | 2.378310 | -0.799910 | -0.000445 |
| 11 | 1 | 0 | -2.326059 | 1.305695 | -0.000208 |

**010**

Standard orientation:

| Center Number | Atomic Number | Atomic Type | Coordinates (Angstroms) X | Y | Z |
|---|---|---|---|---|---|
| 1 | 6 | 0 | 0.723696 | -0.029358 | 0.000064 |
| 2 | 6 | 0 | -0.072593 | 1.148483 | -0.000004 |
| 3 | 6 | 0 | -1.386963 | 0.738567 | -0.000051 |
| 4 | 1 | 0 | 0.277013 | 2.171533 | 0.000079 |
| 5 | 1 | 0 | -2.299617 | 1.314056 | 0.000089 |
| 6 | 8 | 0 | 2.083806 | -0.122768 | -0.000058 |
| 7 | 7 | 0 | -1.404947 | -0.623344 | -0.000097 |
| 8 | 1 | 0 | -2.233388 | -1.190605 | 0.000416 |
| 9 | 6 | 0 | -0.118050 | -1.115076 | 0.000078 |
| 10 | 1 | 0 | 0.097331 | -2.170693 | -0.000164 |
| 11 | 1 | 0 | 2.446301 | 0.765569 | 0.000202 |

**011**

Standard orientation:

| Center Number | Atomic Number | Atomic Type | Coordinates (Angstroms) X | Y | Z |
|---|---|---|---|---|---|
| 1 | 6 | 0 | 0.723400 | 0.020352 | -0.000003 |
| 2 | 6 | 0 | -0.121122 | 1.160868 | -0.000062 |
| 3 | 6 | 0 | -1.414683 | 0.694719 | 0.000059 |
| 4 | 1 | 0 | 0.197245 | 2.191955 | -0.000118 |
| 5 | 1 | 0 | -2.351360 | 1.230514 | 0.000123 |
| 6 | 8 | 0 | 2.083324 | 0.103605 | -0.000114 |
| 7 | 1 | 0 | 2.446938 | -0.784911 | 0.001165 |
| 8 | 7 | 0 | -1.379613 | -0.668367 | -0.000052 |
| 9 | 1 | 0 | -2.183416 | -1.269688 | 0.000032 |
| 10 | 6 | 0 | -0.070775 | -1.103421 | 0.000022 |
| 11 | 1 | 0 | 0.180370 | -2.153250 | -0.000022 |

**012**

Standard orientation:

| Center Number | Atomic Number | Atomic Type | Coordinates (Angstroms) X | Y | Z |
|---|---|---|---|---|---|
| 1 | 6 | 0 | 0.685738 | 0.105565 | -0.000025 |
| 2 | 6 | 0 | -0.166794 | 1.177205 | 0.000113 |
| 3 | 6 | 0 | -1.490604 | 0.642120 | -0.000094 |
| 4 | 6 | 0 | -1.399344 | -0.721757 | 0.000224 |
| 5 | 1 | 0 | 0.130085 | 2.214121 | 0.000224 |
| 6 | 1 | 0 | -2.408954 | 1.210707 | -0.000129 |
| 7 | 1 | 0 | -2.154420 | -1.491469 | 0.000439 |
| 8 | 8 | 0 | 2.041677 | 0.117982 | -0.000310 |
| 9 | 7 | 0 | -0.053175 | -1.047716 | -0.000042 |
| 10 | 1 | 0 | 0.312157 | -1.984272 | -0.000627 |

| | | | | | |
|---|---|---|---|---|---|
| 11 | 1 | 0 | 2.385962 | -0.777730 | 0.001554 |

**013**

Standard orientation:

| Center Number | Atomic Number | Atomic Type | Coordinates (Angstroms) X | Y | Z |
|---|---|---|---|---|---|
| 1 | 6 | 0 | -1.369643 | 0.790105 | -0.000096 |
| 2 | 6 | 0 | -0.086548 | 1.168253 | -0.000261 |
| 3 | 6 | 0 | 0.695331 | -0.084738 | 0.000212 |
| 4 | 7 | 0 | -0.007466 | -1.155577 | 0.000169 |
| 5 | 6 | 0 | -1.388426 | -0.712142 | -0.000098 |
| 6 | 1 | 0 | -1.912902 | -1.108960 | 0.878902 |
| 7 | 1 | 0 | 0.316423 | 2.174488 | -0.000487 |
| 8 | 1 | 0 | -1.912745 | -1.109141 | -0.879107 |
| 9 | 8 | 0 | 2.039500 | -0.128624 | 0.000124 |
| 10 | 1 | 0 | -2.245963 | 1.425935 | -0.000224 |
| 11 | 1 | 0 | 2.387168 | 0.766829 | 0.000198 |

**014**

Standard orientation:

| Center Number | Atomic Number | Atomic Type | Coordinates (Angstroms) X | Y | Z |
|---|---|---|---|---|---|
| 1 | 6 | 0 | 0.816656 | -0.012650 | -0.000005 |
| 2 | 6 | 0 | -1.314719 | 0.809443 | -0.000016 |
| 3 | 6 | 0 | -0.041987 | 1.194135 | -0.000006 |
| 4 | 1 | 0 | 0.349988 | 2.202260 | 0.000000 |
| 5 | 1 | 0 | 2.529051 | 0.738034 | 0.000049 |
| 6 | 7 | 0 | 2.072430 | -0.171319 | 0.000026 |
| 7 | 6 | 0 | -1.362518 | -0.688757 | 0.000006 |
| 8 | 1 | 0 | -1.864854 | -1.092158 | -0.887756 |
| 9 | 8 | 0 | -0.005510 | -1.100827 | -0.000017 |
| 10 | 1 | 0 | -1.864818 | -1.092118 | 0.887809 |
| 11 | 1 | 0 | -2.196886 | 1.436812 | -0.000025 |

**015**

Standard orientation:

| Center Number | Atomic Number | Atomic Type | Coordinates (Angstroms) X | Y | Z |
|---|---|---|---|---|---|
| 1 | 6 | 0 | -1.355994 | 0.751223 | -0.000030 |
| 2 | 6 | 0 | -0.106843 | 1.208502 | 0.000054 |
| 3 | 6 | 0 | 0.813973 | 0.059774 | -0.000196 |
| 4 | 6 | 0 | -1.324926 | -0.748575 | 0.000029 |
| 5 | 1 | 0 | -1.800421 | -1.181347 | 0.888406 |
| 6 | 1 | 0 | 0.241707 | 2.230857 | 0.000128 |
| 7 | 1 | 0 | -1.800456 | -1.181446 | -0.888281 |
| 8 | 1 | 0 | -2.272616 | 1.326822 | -0.000030 |
| 9 | 7 | 0 | 2.077892 | 0.100052 | 0.000050 |
| 10 | 1 | 0 | 2.460876 | -0.845790 | 0.000135 |
| 11 | 8 | 0 | 0.058551 | -1.084375 | 0.000019 |

**016**

Standard orientation:

| Center Number | Atomic Number | Atomic Type | Coordinates (Angstroms) X | Y | Z |
|---|---|---|---|---|---|
| 1 | 6 | 0 | -0.703576 | 0.108192 | -0.000109 |
| 2 | 6 | 0 | 0.134329 | 1.158971 | 0.000141 |
| 3 | 6 | 0 | 1.210718 | -0.885834 | 0.000154 |
| 4 | 6 | 0 | 1.516686 | 0.583397 | -0.000009 |
| 5 | 1 | 0 | 2.114006 | 0.854802 | -0.881050 |
| 6 | 7 | 0 | -0.046900 | -1.148206 | -0.000153 |
| 7 | 8 | 0 | -2.045340 | 0.103607 | -0.000077 |
| 8 | 1 | 0 | -2.318535 | -0.820384 | 0.001011 |
| 9 | 1 | 0 | -0.124553 | 2.206001 | 0.000400 |
| 10 | 1 | 0 | 1.955753 | -1.676090 | 0.000626 |
| 11 | 1 | 0 | 2.115409 | 0.855903 | 0.879640 |

**017**

Standard orientation:

| Center Number | Atomic Number | Atomic Type | Coordinates (Angstroms) X | Y | Z |
|---|---|---|---|---|---|
| 1 | 6 | 0 | -1.432296 | 0.660551 | -0.000012 |
| 2 | 6 | 0 | 0.710644 | 0.059451 | -0.000129 |
| 3 | 6 | 0 | -0.061608 | 1.165832 | -0.000051 |
| 4 | 1 | 0 | 0.244843 | 2.203425 | 0.000183 |
| 5 | 6 | 0 | -0.190028 | -1.140159 | 0.000105 |
| 6 | 8 | 0 | 2.042001 | -0.103798 | -0.000291 |
| 7 | 1 | 0 | 2.469757 | 0.758137 | 0.001778 |
| 8 | 7 | 0 | -1.550328 | -0.623165 | -0.000107 |
| 9 | 1 | 0 | -2.313913 | 1.298255 | 0.000014 |
| 10 | 1 | 0 | -0.022215 | -1.771558 | -0.880213 |
| 11 | 1 | 0 | -0.022463 | -1.769768 | 0.881840 |

**018**

Standard orientation:

| Center Number | Atomic Number | Atomic Type | Coordinates (Angstroms) X | Y | Z |
|---|---|---|---|---|---|
| 1 | 6 | 0 | -0.704839 | 0.028517 | 0.000007 |
| 2 | 6 | 0 | 0.062653 | 1.135862 | 0.000081 |
| 3 | 6 | 0 | 1.266184 | -0.828676 | -0.000191 |
| 4 | 6 | 0 | 1.480798 | 0.657839 | 0.000028 |
| 5 | 1 | 0 | 2.057384 | 0.969646 | -0.881096 |
| 6 | 7 | 0 | 0.031008 | -1.178764 | -0.000019 |
| 7 | 8 | 0 | -2.039619 | -0.124374 | -0.000042 |
| 8 | 1 | 0 | -0.258088 | 2.167700 | -0.000522 |
| 9 | 1 | 0 | 2.063015 | -1.567048 | 0.000604 |
| 10 | 1 | 0 | 2.057332 | 0.969160 | 0.881367 |
| 11 | 1 | 0 | -2.448522 | 0.745625 | 0.000565 |

**019**

Standard orientation:

---------------------------------------------------------------------

| Center Number | Atomic Number | Atomic Type | Coordinates (Angstroms) X | Y | Z |
|---|---|---|---|---|---|
| 1 | 6 | 0 | 1.458181 | 0.611402 | -0.000040 |
| 2 | 6 | 0 | -0.709121 | 0.111488 | -0.000086 |
| 3 | 6 | 0 | 0.114114 | 1.177704 | 0.000146 |
| 4 | 1 | 0 | -0.154571 | 2.223898 | 0.000696 |
| 5 | 6 | 0 | 0.141215 | -1.131808 | 0.000237 |
| 6 | 8 | 0 | -2.052674 | 0.123736 | -0.000194 |
| 7 | 1 | 0 | -2.385323 | -0.778006 | -0.000237 |
| 8 | 7 | 0 | 1.522797 | -0.677741 | -0.000415 |
| 9 | 1 | 0 | 2.368875 | 1.206448 | -0.000214 |
| 10 | 1 | 0 | -0.046135 | -1.754119 | 0.885106 |
| 11 | 1 | 0 | -0.047369 | -1.756641 | -0.882430 |

**020**

Standard orientation:

| Center Number | Atomic Number | Atomic Type | Coordinates (Angstroms) X | Y | Z |
|---|---|---|---|---|---|
| 1 | 6 | 0 | 0.143006 | 1.130653 | -0.000123 |
| 2 | 6 | 0 | -0.731622 | -0.049862 | 0.000015 |
| 3 | 6 | 0 | 1.470346 | -0.616187 | -0.000016 |
| 4 | 8 | 0 | -2.073028 | 0.097991 | 0.000071 |
| 5 | 6 | 0 | 0.064085 | -1.133546 | -0.000005 |
| 6 | 1 | 0 | -0.222731 | -2.176857 | 0.000025 |
| 7 | 1 | 0 | 2.043980 | -0.936190 | -0.879059 |
| 8 | 7 | 0 | 1.392844 | 0.841855 | 0.000022 |
| 9 | 1 | 0 | -0.218971 | 2.155762 | -0.000093 |
| 10 | 1 | 0 | 2.043977 | -0.936199 | 0.879025 |
| 11 | 1 | 0 | -2.486828 | -0.769776 | 0.000144 |

**021**

Standard orientation:

| Center Number | Atomic Number | Atomic Type | Coordinates (Angstroms) X | Y | Z |
|---|---|---|---|---|---|
| 1 | 6 | 0 | 1.493792 | -0.568411 | -0.000109 |
| 2 | 6 | 0 | -0.730866 | -0.098254 | 0.000090 |
| 3 | 6 | 0 | 0.112401 | -1.143793 | 0.000022 |
| 4 | 1 | 0 | -0.140173 | -2.194252 | -0.000083 |
| 5 | 6 | 0 | 0.097650 | 1.117771 | 0.000240 |
| 6 | 8 | 0 | -2.082153 | -0.128323 | 0.000040 |
| 7 | 7 | 0 | 1.359084 | 0.885570 | 0.000037 |
| 8 | 1 | 0 | 2.081575 | -0.863487 | 0.878368 |
| 9 | 1 | 0 | 2.081422 | -0.863441 | -0.878709 |
| 10 | 1 | 0 | -0.295160 | 2.134758 | -0.000852 |
| 11 | 1 | 0 | -2.421887 | 0.770136 | -0.000762 |

**022**

Standard orientation:

| Center Number | Atomic Number | Atomic Type | Coordinates (Angstroms) X | Y | Z |
|---|---|---|---|---|---|
| 1 | 6 | 0 | -1.309766 | 0.729739 | -0.000034 |
| 2 | 6 | 0 | -0.051952 | 1.198589 | -0.000024 |

| | | | | | |
|---|---|---|---|---|---|
| 3 | 6 | 0 | 0.835982 | 0.036377 | 0.000034 |
| 4 | 1 | 0 | 0.246580 | 2.236228 | -0.000046 |
| 5 | 1 | 0 | -2.248161 | 1.270091 | -0.000072 |
| 6 | 7 | 0 | 2.102224 | -0.105122 | 0.000053 |
| 7 | 6 | 0 | -0.107866 | -1.156313 | 0.000010 |
| 8 | 1 | 0 | 0.014699 | -1.776130 | 0.891435 |
| 9 | 1 | 0 | 0.014750 | -1.776146 | -0.891397 |
| 10 | 8 | 0 | -1.437275 | -0.609762 | -0.000031 |
| 11 | 1 | 0 | 2.556386 | 0.809558 | 0.000034 |

**023**

Standard orientation:

| Center Number | Atomic Number | Atomic Type | Coordinates (Angstroms) X | Y | Z |
|---|---|---|---|---|---|
| 1 | 6 | 0 | -0.708894 | 0.027457 | -0.000167 |
| 2 | 6 | 0 | 0.058847 | 1.133283 | -0.000034 |
| 3 | 6 | 0 | 1.527869 | -0.474720 | -0.000083 |
| 4 | 8 | 0 | -2.047263 | -0.136125 | -0.000079 |
| 5 | 1 | 0 | -0.249628 | 2.170770 | -0.000108 |
| 6 | 1 | 0 | 2.485895 | -0.985451 | -0.000782 |
| 7 | 1 | 0 | -2.468407 | 0.728871 | 0.000408 |
| 8 | 7 | 0 | 1.441811 | 0.807377 | 0.000119 |
| 9 | 6 | 0 | 0.192460 | -1.165914 | 0.000042 |
| 10 | 1 | 0 | 0.047738 | -1.799768 | -0.882428 |
| 11 | 1 | 0 | 0.048140 | -1.797693 | 0.884156 |

**024**

Standard orientation:

| Center Number | Atomic Number | Atomic Type | Coordinates (Angstroms) X | Y | Z |
|---|---|---|---|---|---|
| 1 | 6 | 0 | -1.341997 | 0.683044 | 0.000007 |
| 2 | 6 | 0 | -0.108089 | 1.206430 | -0.000050 |
| 3 | 6 | 0 | 0.833837 | 0.092464 | 0.000034 |
| 4 | 1 | 0 | 0.158372 | 2.251592 | -0.000090 |
| 5 | 1 | 0 | -2.305484 | 1.176011 | -0.000012 |
| 6 | 7 | 0 | 2.105902 | 0.170527 | 0.000059 |
| 7 | 1 | 0 | 2.522934 | -0.762606 | 0.000052 |
| 8 | 6 | 0 | -0.057639 | -1.147566 | 0.000013 |
| 9 | 1 | 0 | 0.092951 | -1.762588 | 0.892191 |
| 10 | 1 | 0 | 0.093042 | -1.762600 | -0.892144 |
| 11 | 8 | 0 | -1.407474 | -0.667466 | -0.000054 |

**025**

Standard orientation:

| Center Number | Atomic Number | Atomic Type | Coordinates (Angstroms) X | Y | Z |
|---|---|---|---|---|---|
| 1 | 6 | 0 | 0.123619 | 1.153893 | 0.000083 |
| 2 | 6 | 0 | 1.392781 | -0.612952 | 0.000077 |
| 3 | 1 | 0 | -0.214004 | 2.185671 | 0.000137 |
| 4 | 1 | 0 | 1.910129 | -0.980256 | -0.893239 |
| 5 | 8 | 0 | 0.046161 | -1.098565 | -0.000047 |
| 6 | 6 | 0 | -2.104604 | -0.051468 | -0.000196 |

| | | | | | |
|---|---|---|---|---|---|
| 7 | 1 | 0 | -2.665432 | 0.874237 | -0.000355 |
| 8 | 1 | 0 | -2.643688 | -0.989866 | 0.000135 |
| 9 | 6 | 0 | -0.772368 | -0.012324 | -0.000084 |
| 10 | 1 | 0 | 1.910010 | -0.980355 | 0.893420 |
| 11 | 7 | 0 | 1.356733 | 0.830884 | 0.000143 |

**026**

Standard orientation:

| Center Number | Atomic Number | Atomic Type | Coordinates (Angstroms) X | Y | Z |
|---|---|---|---|---|---|
| 1 | 6 | 0 | 0.709529 | -0.077696 | -0.000008 |
| 2 | 6 | 0 | -0.106974 | -1.145618 | -0.000180 |
| 3 | 6 | 0 | -1.509504 | 0.524460 | -0.000095 |
| 4 | 8 | 0 | 2.060764 | -0.089702 | 0.000166 |
| 5 | 1 | 0 | 0.163181 | -2.191655 | -0.000261 |
| 6 | 1 | 0 | -2.443671 | 1.077487 | -0.000110 |
| 7 | 7 | 0 | -1.473373 | -0.759138 | -0.000247 |
| 8 | 6 | 0 | -0.144890 | 1.158977 | 0.000234 |
| 9 | 1 | 0 | 0.014664 | 1.786878 | -0.885508 |
| 10 | 1 | 0 | 0.014313 | 1.785875 | 0.886780 |
| 11 | 1 | 0 | 2.390049 | 0.812257 | -0.000203 |

**027**

Standard orientation:

| Center Number | Atomic Number | Atomic Type | Coordinates (Angstroms) X | Y | Z |
|---|---|---|---|---|---|
| 1 | 6 | 0 | 0.616389 | 0.111584 | -0.000170 |
| 2 | 6 | 0 | -0.258788 | 1.152088 | 0.000103 |
| 3 | 6 | 0 | -1.526743 | 0.514358 | 0.000115 |
| 4 | 8 | 0 | -0.065291 | -1.044863 | 0.000518 |
| 5 | 1 | 0 | -0.035727 | 2.206844 | 0.000200 |
| 6 | 1 | 0 | -2.511194 | 0.961850 | 0.000140 |
| 7 | 7 | 0 | -1.423096 | -0.789207 | -0.000512 |
| 8 | 6 | 0 | 2.100155 | 0.014074 | -0.000134 |
| 9 | 1 | 0 | 2.451914 | -0.523794 | 0.884457 |
| 10 | 1 | 0 | 2.451775 | -0.525819 | -0.883539 |
| 11 | 1 | 0 | 2.541153 | 1.011649 | -0.001305 |

**028**

Standard orientation:

| Center Number | Atomic Number | Atomic Type | Coordinates (Angstroms) X | Y | Z |
|---|---|---|---|---|---|
| 1 | 6 | 0 | -0.709395 | 0.010946 | 0.388115 |
| 2 | 6 | 0 | 1.427298 | 0.678106 | -0.149145 |
| 3 | 8 | 0 | -1.937439 | 0.017339 | -0.274515 |
| 4 | 1 | 0 | 2.346392 | 1.210935 | -0.356964 |
| 5 | 6 | 0 | 0.214974 | 1.179374 | 0.105567 |
| 6 | 7 | 0 | 0.086105 | -1.197328 | 0.144305 |
| 7 | 6 | 0 | 1.276143 | -0.792733 | -0.109412 |
| 8 | 1 | 0 | 2.084579 | -1.498471 | -0.288387 |
| 9 | 1 | 0 | -0.941009 | 0.012232 | 1.463030 |

| | | | | | |
|---|---|---|---|---|---|
| 10 | 1 | 0 | -0.088546 | 2.216462 | 0.162275 |
| 11 | 1 | 0 | -1.758752 | -0.152727 | -1.204719 |

**029**

Standard orientation:

| Center Number | Atomic Number | Atomic Type | Coordinates (Angstroms) X | Y | Z |
|---|---|---|---|---|---|
| 1 | 6 | 0 | 0.631004 | 0.025081 | 0.000219 |
| 2 | 7 | 0 | -0.034425 | -1.105221 | 0.000201 |
| 3 | 6 | 0 | 2.128706 | 0.026056 | -0.000186 |
| 4 | 1 | 0 | 2.515194 | -0.489460 | 0.882153 |
| 5 | 1 | 0 | 2.514833 | -0.487120 | -0.884057 |
| 6 | 1 | 0 | 2.511350 | 1.048139 | 0.001050 |
| 7 | 6 | 0 | -1.480890 | 0.571101 | -0.000158 |
| 8 | 1 | 0 | -2.486467 | 0.963421 | -0.000506 |
| 9 | 6 | 0 | -0.253111 | 1.143004 | 0.000190 |
| 10 | 1 | 0 | -0.000621 | 2.191262 | 0.000332 |
| 11 | 8 | 0 | -1.370946 | -0.760144 | -0.000096 |

**030**

Standard orientation:

| Center Number | Atomic Number | Atomic Type | Coordinates (Angstroms) X | Y | Z |
|---|---|---|---|---|---|
| 1 | 6 | 0 | 0.235663 | 1.094195 | 0.000527 |
| 2 | 7 | 0 | 1.488293 | 0.719924 | -0.000394 |
| 3 | 6 | 0 | 0.183578 | -1.067043 | 0.000436 |
| 4 | 1 | 0 | 0.030263 | -2.135913 | 0.000519 |
| 5 | 6 | 0 | -0.666740 | -0.009308 | -0.000026 |
| 6 | 8 | 0 | 1.458839 | -0.656317 | -0.000211 |
| 7 | 6 | 0 | -2.161764 | 0.008230 | -0.000246 |
| 8 | 1 | 0 | -2.551793 | 0.523409 | 0.882554 |
| 9 | 1 | 0 | -2.551654 | 0.523061 | -0.883312 |
| 10 | 1 | 0 | -2.564310 | -1.007222 | -0.000081 |
| 11 | 1 | 0 | 0.004304 | 2.151284 | 0.000620 |

**031**

Standard orientation:

| Center Number | Atomic Number | Atomic Type | Coordinates (Angstroms) X | Y | Z |
|---|---|---|---|---|---|
| 1 | 6 | 0 | -0.703019 | -0.016181 | 0.368575 |
| 2 | 6 | 0 | 1.419888 | 0.703337 | -0.142227 |
| 3 | 8 | 0 | -1.874548 | -0.091990 | -0.395357 |
| 4 | 1 | 0 | 2.328285 | 1.257851 | -0.340252 |
| 5 | 6 | 0 | 0.194872 | 1.177369 | 0.101835 |
| 6 | 1 | 0 | -2.483011 | 0.577868 | -0.073317 |
| 7 | 7 | 0 | 0.115513 | -1.198747 | 0.130575 |
| 8 | 6 | 0 | 1.299480 | -0.769452 | -0.107170 |
| 9 | 1 | 0 | 2.123971 | -1.459459 | -0.271717 |
| 10 | 1 | 0 | -0.926532 | -0.030924 | 1.451918 |
| 11 | 1 | 0 | -0.122238 | 2.211381 | 0.156123 |

**032**

Standard orientation:

| Center Number | Atomic Number | Atomic Type | Coordinates (Angstroms) X | Y | Z |
|---|---|---|---|---|---|
| 1 | 6 | 0 | 0.792418 | 0.006612 | 0.000183 |
| 2 | 6 | 0 | -0.161256 | -1.158341 | 0.000381 |
| 3 | 7 | 0 | -1.354703 | 0.816794 | -0.000276 |
| 4 | 6 | 0 | -0.116789 | 1.149415 | 0.000477 |
| 5 | 1 | 0 | 0.157190 | 2.196325 | 0.000156 |
| 6 | 1 | 0 | -0.069464 | -1.786226 | -0.891691 |
| 7 | 8 | 0 | -1.468656 | -0.556588 | -0.000365 |
| 8 | 1 | 0 | -0.069824 | -1.785576 | 0.892995 |
| 9 | 6 | 0 | 2.124903 | 0.013644 | -0.000297 |
| 10 | 1 | 0 | 2.701867 | -0.904805 | -0.000946 |
| 11 | 1 | 0 | 2.676741 | 0.947446 | -0.000129 |

**100**

Standard orientation:

| Center Number | Atomic Number | Atomic Type | Coordinates (Angstroms) X | Y | Z |
|---|---|---|---|---|---|
| 1 | 6 | 0 | 1.418321 | -0.221029 | 0.000452 |
| 2 | 6 | 0 | 0.761579 | 1.132795 | -0.000242 |
| 3 | 6 | 0 | -0.559828 | 1.236862 | -0.000164 |
| 4 | 6 | 0 | -0.773118 | -1.070905 | -0.000199 |
| 5 | 7 | 0 | 0.460156 | -1.324106 | -0.000472 |
| 6 | 1 | 0 | -1.120804 | 2.162223 | -0.000485 |
| 7 | 1 | 0 | 2.072414 | -0.340065 | -0.873224 |
| 8 | 1 | 0 | 1.367120 | 2.031866 | -0.001007 |
| 9 | 1 | 0 | -1.513732 | -1.866210 | -0.000602 |
| 10 | 8 | 0 | -1.397312 | 0.144342 | 0.000517 |
| 11 | 1 | 0 | 2.070686 | -0.340150 | 0.875408 |

**101**

Standard orientation:

| Center Number | Atomic Number | Atomic Type | Coordinates (Angstroms) X | Y | Z |
|---|---|---|---|---|---|
| 1 | 6 | 0 | -0.991966 | -0.948549 | -0.056237 |
| 2 | 6 | 0 | -1.278497 | 0.511328 | 0.137992 |
| 3 | 6 | 0 | 1.081645 | 0.756685 | 0.004059 |
| 4 | 6 | 0 | 0.275189 | -1.360811 | -0.038837 |
| 5 | 1 | 0 | -1.821037 | -1.634271 | -0.188771 |
| 6 | 1 | 0 | -1.579394 | 0.735477 | 1.172578 |
| 7 | 1 | 0 | 1.863082 | 1.512768 | 0.005131 |
| 8 | 1 | 0 | 0.545969 | -2.403583 | -0.165193 |
| 9 | 7 | 0 | 1.365322 | -0.479642 | 0.114182 |
| 10 | 8 | 0 | -0.126285 | 1.316277 | -0.172732 |
| 11 | 1 | 0 | -2.073824 | 0.864973 | -0.523028 |

**102**

Standard orientation:

| Center | Atomic | Atomic | Coordinates (Angstroms) |
|---|---|---|---|

| Number | Number | Type | X | Y | Z |
|---|---|---|---|---|---|
| 1 | 6 | 0 | 0.223335 | 1.353025 | -0.079628 |
| 2 | 6 | 0 | 1.312802 | 0.419561 | 0.166119 |
| 3 | 6 | 0 | 1.016650 | -0.879789 | -0.018483 |
| 4 | 6 | 0 | -1.241255 | -0.372933 | 0.222823 |
| 5 | 7 | 0 | -1.007828 | 1.004023 | -0.136075 |
| 6 | 1 | 0 | 1.747472 | -1.679531 | -0.085298 |
| 7 | 1 | 0 | 2.333034 | 0.750322 | 0.302437 |
| 8 | 1 | 0 | -1.304774 | -0.455091 | 1.320685 |
| 9 | 8 | 0 | -0.235503 | -1.303517 | -0.226281 |
| 10 | 1 | 0 | -2.168097 | -0.730003 | -0.224725 |
| 11 | 1 | 0 | 0.462005 | 2.395089 | -0.295300 |

**103**

Standard orientation:

| Center Number | Atomic Number | Atomic Type | Coordinates (Angstroms) X | Y | Z |
|---|---|---|---|---|---|
| 1 | 6 | 0 | 1.208468 | 0.614617 | 0.159055 |
| 2 | 6 | 0 | 1.121121 | -0.712828 | -0.021043 |
| 3 | 6 | 0 | -1.163876 | -0.589359 | 0.265304 |
| 4 | 1 | 0 | 1.967741 | -1.387747 | -0.067816 |
| 5 | 1 | 0 | 2.155353 | 1.099499 | 0.355601 |
| 6 | 1 | 0 | -1.167514 | -0.693985 | 1.363630 |
| 7 | 8 | 0 | -0.063030 | -1.322950 | -0.265146 |
| 8 | 1 | 0 | -2.067932 | -1.046478 | -0.137956 |
| 9 | 6 | 0 | -1.033197 | 0.866336 | -0.111200 |
| 10 | 1 | 0 | -1.914944 | 1.436049 | -0.402021 |
| 11 | 7 | 0 | 0.105206 | 1.443380 | -0.106137 |

**104**

Standard orientation:

| Center Number | Atomic Number | Atomic Type | Coordinates (Angstroms) X | Y | Z |
|---|---|---|---|---|---|
| 1 | 6 | 0 | -0.009587 | 0.644889 | 1.176075 |
| 2 | 6 | 0 | -0.009587 | -0.684184 | 1.153367 |
| 3 | 6 | 0 | -0.009587 | -0.684184 | -1.153367 |
| 4 | 1 | 0 | -0.014491 | -1.296175 | 2.044155 |
| 5 | 1 | 0 | -0.025606 | 1.177217 | 2.118386 |
| 6 | 8 | 0 | 0.036103 | -1.442790 | -0.000000 |
| 7 | 1 | 0 | -0.014491 | -1.296175 | -2.044155 |
| 8 | 6 | 0 | -0.009587 | 0.644889 | -1.176075 |
| 9 | 1 | 0 | -0.025606 | 1.177217 | -2.118386 |
| 10 | 7 | 0 | 0.084341 | 1.434667 | 0.000000 |
| 11 | 1 | 0 | -0.568930 | 2.209106 | 0.000000 |

**105**

Standard orientation:

| Center Number | Atomic Number | Atomic Type | Coordinates (Angstroms) X | Y | Z |
|---|---|---|---|---|---|
| 1 | 6 | 0 | -0.881760 | -1.008925 | 0.000061 |
| 2 | 6 | 0 | 1.440490 | -0.229685 | -0.000176 |

| | | | | | |
|---|---|---|---|---|---|
| 3 | 1 | 0 | -1.687987 | -1.732574 | 0.000252 |
| 4 | 1 | 0 | 2.101901 | -0.288496 | 0.875306 |
| 5 | 6 | 0 | 0.735712 | 1.098294 | 0.000155 |
| 6 | 1 | 0 | 1.332971 | 2.007725 | 0.000392 |
| 7 | 7 | 0 | -0.516554 | 1.338090 | 0.000063 |
| 8 | 8 | 0 | -1.395331 | 0.254940 | -0.000182 |
| 9 | 6 | 0 | 0.409228 | -1.317390 | 0.000111 |
| 10 | 1 | 0 | 0.708336 | -2.358191 | 0.000308 |
| 11 | 1 | 0 | 2.101281 | -0.288377 | -0.876150 |

---------------------------------------------------------------------

**106**

Standard orientation:

| Center Number | Atomic Number | Atomic Type | Coordinates (Angstroms) X | Y | Z |
|---|---|---|---|---|---|
| 1 | 6 | 0 | -1.276900 | -0.305941 | 0.249975 |
| 2 | 6 | 0 | 0.456550 | 1.327463 | -0.078555 |
| 3 | 1 | 0 | -2.240490 | -0.569314 | -0.189396 |
| 4 | 1 | 0 | 0.892134 | 2.298509 | -0.284543 |
| 5 | 6 | 0 | 1.335514 | 0.184099 | 0.143757 |
| 6 | 1 | 0 | 2.390622 | 0.317957 | 0.361257 |
| 7 | 7 | 0 | 0.975299 | -1.042476 | 0.005198 |
| 8 | 8 | 0 | -0.360005 | -1.247969 | -0.302728 |
| 9 | 6 | 0 | -0.857312 | 1.097820 | -0.076117 |
| 10 | 1 | 0 | -1.596181 | 1.866178 | -0.277784 |
| 11 | 1 | 0 | -1.340254 | -0.452895 | 1.341539 |

---------------------------------------------------------------------

**107**

Standard orientation:

| Center Number | Atomic Number | Atomic Type | Coordinates (Angstroms) X | Y | Z |
|---|---|---|---|---|---|
| 1 | 6 | 0 | 0.562522 | 1.265783 | 0.156157 |
| 2 | 1 | 0 | 1.005968 | 2.227845 | 0.375439 |
| 3 | 6 | 0 | -0.863443 | 1.105231 | -0.090170 |
| 4 | 1 | 0 | -1.482720 | 1.959508 | -0.338434 |
| 5 | 6 | 0 | 1.329116 | 0.177993 | 0.003661 |
| 6 | 1 | 0 | 2.413532 | 0.160835 | 0.016060 |
| 7 | 6 | 0 | -1.363290 | -0.134895 | -0.074984 |
| 8 | 1 | 0 | -2.400440 | -0.385267 | -0.268408 |
| 9 | 7 | 0 | -0.511274 | -1.252222 | 0.211763 |
| 10 | 1 | 0 | -0.379249 | -1.339723 | 1.224040 |
| 11 | 8 | 0 | 0.804049 | -1.042789 | -0.307378 |

---------------------------------------------------------------------

**200**

Standard orientation:

| Center Number | Atomic Number | Atomic Type | Coordinates (Angstroms) X | Y | Z |
|---|---|---|---|---|---|
| 1 | 6 | 0 | 0.937211 | 1.159926 | 0.000441 |
| 2 | 6 | 0 | -0.605050 | 1.335846 | -0.000894 |
| 3 | 1 | 0 | 1.454625 | 1.530248 | -0.887820 |
| 4 | 1 | 0 | 1.452619 | 1.529585 | 0.890174 |
| 5 | 1 | 0 | -1.013160 | 1.821160 | -0.891021 |
| 6 | 1 | 0 | -1.014762 | 1.822603 | 0.887671 |

| | | | | | |
|---|---|---|---|---|---|
| 7 | 6 | 0 | 0.745794 | -0.370097 | 0.000089 |
| 8 | 6 | 0 | -0.776222 | -0.191109 | -0.000064 |
| 9 | 7 | 0 | -1.709593 | -1.038105 | 0.000708 |
| 10 | 1 | 0 | -2.620516 | -0.570166 | 0.000788 |
| 11 | 8 | 0 | 1.487243 | -1.309260 | -0.000273 |

---------------------------------------------------------------------

**201**

Standard orientation:

| Center Number | Atomic Number | Atomic Type | Coordinates (Angstroms) X | Y | Z |
|---|---|---|---|---|---|
| 1 | 6 | 0 | 1.030001 | 1.088275 | 0.000225 |
| 2 | 6 | 0 | -0.494353 | 1.399904 | -0.000265 |
| 3 | 1 | 0 | 1.579951 | 1.408921 | -0.888184 |
| 4 | 1 | 0 | 1.579446 | 1.408896 | 0.888960 |
| 5 | 1 | 0 | -0.859828 | 1.918906 | -0.889007 |
| 6 | 1 | 0 | -0.860295 | 1.919376 | 0.887999 |
| 7 | 6 | 0 | 0.700844 | -0.413857 | 0.000116 |
| 8 | 8 | 0 | 1.335525 | -1.431179 | -0.000191 |
| 9 | 6 | 0 | -0.799455 | -0.095850 | 0.000091 |
| 10 | 7 | 0 | -1.871389 | -0.759712 | 0.000099 |
| 11 | 1 | 0 | -1.645972 | -1.759517 | 0.000071 |

---------------------------------------------------------------------

**202**

Standard orientation:

| Center Number | Atomic Number | Atomic Type | Coordinates (Angstroms) X | Y | Z |
|---|---|---|---|---|---|
| 1 | 6 | 0 | -0.091167 | 1.349717 | 0.054707 |
| 2 | 6 | 0 | -0.693077 | 0.150622 | 0.023678 |
| 3 | 1 | 0 | -0.405286 | 2.382715 | 0.132791 |
| 4 | 7 | 0 | 1.269338 | 0.900093 | -0.160977 |
| 5 | 1 | 0 | 1.967019 | 1.138565 | 0.541343 |
| 6 | 6 | 0 | -2.066119 | -0.410356 | -0.011658 |
| 7 | 1 | 0 | -2.812555 | 0.373910 | 0.135006 |
| 8 | 1 | 0 | -2.266024 | -0.897441 | -0.971576 |
| 9 | 1 | 0 | -2.198138 | -1.164558 | 0.769850 |
| 10 | 6 | 0 | 0.692968 | -0.438689 | -0.040365 |
| 11 | 8 | 0 | 1.221749 | -1.505201 | 0.045156 |

---------------------------------------------------------------------

**204**

Standard orientation:

| Center Number | Atomic Number | Atomic Type | Coordinates (Angstroms) X | Y | Z |
|---|---|---|---|---|---|
| 1 | 6 | 0 | -1.161668 | 1.061042 | -0.000034 |
| 2 | 1 | 0 | -1.728316 | 1.343843 | -0.894125 |
| 3 | 1 | 0 | -1.728420 | 1.343850 | 0.893988 |
| 4 | 6 | 0 | 0.726094 | 0.121765 | 0.000007 |
| 5 | 6 | 0 | 0.327671 | 1.407955 | 0.000052 |
| 6 | 6 | 0 | -0.652035 | -0.396874 | 0.000000 |
| 7 | 7 | 0 | -1.093827 | -1.581644 | 0.000019 |
| 8 | 1 | 0 | -2.114947 | -1.590899 | 0.000017 |
| 9 | 8 | 0 | 1.902757 | -0.510854 | -0.000018 |
| 10 | 1 | 0 | 1.718734 | -1.457622 | -0.000044 |

| | | | | | |
|---|---|---|---|---|---|
| 11 | 1 | 0 | 0.847306 | 2.355850 | 0.000023 |

**205**

Standard orientation:

| Center Number | Atomic Number | Atomic Type | Coordinates (Angstroms) X | Y | Z |
|---|---|---|---|---|---|
| 1 | 6 | 0 | 0.783341 | -1.029991 | 0.021626 |
| 2 | 1 | 0 | 0.816650 | -1.593321 | 0.957524 |
| 3 | 1 | 0 | 0.657898 | -1.706384 | -0.826515 |
| 4 | 6 | 0 | 1.048539 | 0.965817 | 0.009497 |
| 5 | 6 | 0 | -0.081317 | 0.213630 | 0.052391 |
| 6 | 1 | 0 | 1.299207 | 2.021448 | 0.026688 |
| 7 | 7 | 0 | 1.937315 | -0.095203 | -0.141679 |
| 8 | 1 | 0 | 2.758863 | -0.175555 | 0.446226 |
| 9 | 6 | 0 | -1.498716 | 0.471607 | 0.003171 |
| 10 | 1 | 0 | -1.772097 | 1.549456 | -0.002276 |
| 11 | 8 | 0 | -2.354101 | -0.394450 | -0.016250 |

**206**

Standard orientation:

| Center Number | Atomic Number | Atomic Type | Coordinates (Angstroms) X | Y | Z |
|---|---|---|---|---|---|
| 1 | 6 | 0 | -0.728518 | -0.166517 | 0.002355 |
| 2 | 6 | 0 | -1.753900 | -1.006698 | 0.010963 |
| 3 | 1 | 0 | -2.774455 | -0.645704 | 0.019536 |
| 4 | 1 | 0 | -1.570931 | -2.073175 | 0.009809 |
| 5 | 6 | 0 | 0.738156 | -0.188149 | -0.023327 |
| 6 | 6 | 0 | 1.728623 | -1.068406 | 0.008741 |
| 7 | 1 | 0 | 1.510730 | -2.127762 | 0.061796 |
| 8 | 1 | 0 | 2.765320 | -0.754445 | -0.019646 |
| 9 | 7 | 0 | 0.720840 | 1.248265 | -0.117884 |
| 10 | 1 | 0 | 1.052409 | 1.691596 | 0.740418 |
| 11 | 8 | 0 | -0.741890 | 1.218781 | 0.002610 |

**300**

Standard orientation:

| Center Number | Atomic Number | Atomic Type | Coordinates (Angstroms) X | Y | Z |
|---|---|---|---|---|---|
| 1 | 6 | 0 | 1.893547 | -0.752746 | -0.120184 |
| 2 | 6 | 0 | 1.893447 | 0.752850 | -0.120216 |
| 3 | 6 | 0 | 0.729084 | -0.000016 | 0.460164 |
| 4 | 1 | 0 | 1.724626 | -1.252823 | -1.066941 |
| 5 | 1 | 0 | 2.547471 | -1.277213 | 0.567798 |
| 6 | 1 | 0 | 1.724456 | 1.252866 | -1.066992 |
| 7 | 1 | 0 | 2.547306 | 1.277432 | 0.567740 |
| 8 | 1 | 0 | 0.616228 | -0.000011 | 1.540121 |
| 9 | 7 | 0 | -0.491152 | -0.000151 | -0.266942 |
| 10 | 6 | 0 | -1.673804 | -0.000088 | -0.047970 |
| 11 | 8 | 0 | -2.846958 | 0.000100 | 0.037013 |

**301**

Standard orientation:

| Center Number | Atomic Number | Atomic Type | Coordinates (Angstroms) X | Y | Z |
|---|---|---|---|---|---|
| 1 | 6 | 0 | 0.864036 | -1.063583 | 0.605419 |
| 2 | 6 | 0 | 1.150384 | 0.038827 | -0.364598 |
| 3 | 6 | 0 | -0.149086 | -0.724171 | -0.479088 |
| 4 | 1 | 0 | 1.393554 | -2.004989 | 0.511377 |
| 5 | 1 | 0 | 0.596902 | -0.773071 | 1.615670 |
| 6 | 1 | 0 | 1.875489 | -0.157408 | -1.153952 |
| 7 | 1 | 0 | -0.248038 | -1.415821 | -1.309221 |
| 8 | 6 | 0 | -1.380592 | -0.078309 | -0.101919 |
| 9 | 7 | 0 | -2.384123 | 0.417033 | 0.195579 |
| 10 | 8 | 0 | 1.050278 | 1.357524 | 0.042801 |
| 11 | 1 | 0 | 1.760275 | 1.535287 | 0.665786 |

**302**

Standard orientation:

| Center Number | Atomic Number | Atomic Type | Coordinates (Angstroms) X | Y | Z |
|---|---|---|---|---|---|
| 1 | 6 | 0 | 1.135908 | -0.717515 | 0.752785 |
| 2 | 6 | 0 | 1.135854 | -0.717550 | -0.752854 |
| 3 | 6 | 0 | 0.193952 | 0.188063 | 0.000007 |
| 4 | 1 | 0 | 0.725031 | -1.573914 | 1.275420 |
| 5 | 1 | 0 | 1.945878 | -0.201588 | 1.256213 |
| 6 | 1 | 0 | 0.724955 | -1.573985 | -1.275417 |
| 7 | 1 | 0 | 1.945784 | -0.201634 | -1.256356 |
| 8 | 8 | 0 | 0.493939 | 1.552966 | 0.000156 |
| 9 | 1 | 0 | -0.322225 | 2.058004 | -0.001201 |
| 10 | 6 | 0 | -1.216769 | -0.140417 | 0.000056 |
| 11 | 7 | 0 | -2.352086 | -0.372300 | 0.000019 |

**303**

Standard orientation:

| Center Number | Atomic Number | Atomic Type | Coordinates (Angstroms) X | Y | Z |
|---|---|---|---|---|---|
| 1 | 6 | 0 | 1.098942 | -0.719589 | -0.739764 |
| 2 | 6 | 0 | 1.081966 | -0.701732 | 0.775216 |
| 3 | 6 | 0 | 0.160122 | 0.187207 | -0.015445 |
| 4 | 1 | 0 | 1.925825 | -0.229296 | -1.240314 |
| 5 | 1 | 0 | 0.676416 | -1.578396 | -1.247639 |
| 6 | 1 | 0 | 1.904107 | -0.205683 | 1.279221 |
| 7 | 1 | 0 | 0.648371 | -1.548901 | 1.294277 |
| 8 | 6 | 0 | -2.363061 | -0.382989 | 0.004036 |
| 9 | 7 | 0 | -1.211063 | -0.143324 | -0.014242 |
| 10 | 8 | 0 | 0.413392 | 1.539740 | -0.106657 |
| 11 | 1 | 0 | 0.147776 | 1.950232 | 0.723139 |

**304**

Standard orientation:

| Center | Atomic | Atomic | Coordinates (Angstroms) |
|---|---|---|---|

| Number | Number | Type | X | Y | Z |
|---|---|---|---|---|---|
| 1 | 6 | 0 | -1.797265 | -0.757440 | -0.004416 |
| 2 | 6 | 0 | -1.796776 | 0.757932 | -0.003331 |
| 3 | 6 | 0 | -0.574645 | 0.000115 | -0.388165 |
| 4 | 1 | 0 | -1.778804 | -1.248820 | 0.961996 |
| 5 | 1 | 0 | -2.331477 | -1.289233 | -0.783861 |
| 6 | 1 | 0 | -1.777982 | 1.247925 | 0.963777 |
| 7 | 1 | 0 | -2.330651 | 1.291191 | -0.782004 |
| 8 | 1 | 0 | -0.209521 | 0.000739 | -1.408758 |
| 9 | 8 | 0 | 0.485347 | -0.001013 | 0.583599 |
| 10 | 6 | 0 | 1.683858 | -0.000161 | 0.114071 |
| 11 | 7 | 0 | 2.779232 | 0.000517 | -0.275556 |

**305**

Standard orientation:

| Center Number | Atomic Number | Atomic Type | Coordinates (Angstroms) X | Y | Z |
|---|---|---|---|---|---|
| 1 | 6 | 0 | 1.929586 | -0.356449 | -0.156064 |
| 2 | 6 | 0 | 1.256670 | 0.997348 | -0.138511 |
| 3 | 6 | 0 | 0.611180 | -0.180807 | 0.510780 |
| 4 | 1 | 0 | 1.930675 | -0.895874 | -1.096535 |
| 5 | 1 | 0 | 2.793291 | -0.528952 | 0.475872 |
| 6 | 1 | 0 | 0.816509 | 1.345081 | -1.066672 |
| 7 | 1 | 0 | 1.649193 | 1.775907 | 0.505597 |
| 8 | 8 | 0 | -0.469518 | -0.803237 | -0.180204 |
| 9 | 1 | 0 | 0.509495 | -0.241040 | 1.589648 |
| 10 | 7 | 0 | -1.581378 | -0.093978 | -0.051595 |
| 11 | 6 | 0 | -2.609666 | 0.478011 | 0.016277 |

**306**

Standard orientation:

| Center Number | Atomic Number | Atomic Type | Coordinates (Angstroms) X | Y | Z |
|---|---|---|---|---|---|
| 1 | 6 | 0 | 1.891876 | -0.753921 | -0.121951 |
| 2 | 6 | 0 | 1.894466 | 0.751566 | -0.121516 |
| 3 | 6 | 0 | 0.730045 | 0.000716 | 0.461641 |
| 4 | 1 | 0 | 1.719866 | -1.254562 | -1.067824 |
| 5 | 1 | 0 | 2.546832 | -1.278729 | 0.564783 |
| 6 | 1 | 0 | 1.724175 | 1.253362 | -1.067103 |
| 7 | 1 | 0 | 2.551230 | 1.273741 | 0.565489 |
| 8 | 1 | 0 | 0.620298 | 0.000602 | 1.541885 |
| 9 | 6 | 0 | -1.674070 | 0.000239 | -0.047218 |
| 10 | 8 | 0 | -2.847384 | -0.001304 | 0.034982 |
| 11 | 7 | 0 | -0.491033 | 0.003488 | -0.263260 |

**307**

Standard orientation:

| Center Number | Atomic Number | Atomic Type | Coordinates (Angstroms) X | Y | Z |
|---|---|---|---|---|---|
| 1 | 6 | 0 | -0.704740 | -0.071723 | 0.528643 |
| 2 | 1 | 0 | -0.856555 | -0.059506 | 1.613075 |

| | | | | | |
|---|---|---|---|---|---|
| 3 | 6 | 0 | -1.960455 | -0.306031 | -0.292168 |
| 4 | 1 | 0 | -2.387624 | -1.283891 | -0.058325 |
| 5 | 1 | 0 | -2.706120 | 0.463659 | -0.080167 |
| 6 | 1 | 0 | -1.734984 | -0.280953 | -1.361507 |
| 7 | 6 | 0 | 0.426019 | 0.831950 | 0.087332 |
| 8 | 6 | 0 | 0.648699 | -0.618946 | 0.120554 |
| 9 | 8 | 0 | 0.784667 | 1.942729 | -0.180310 |
| 10 | 7 | 0 | 1.249993 | -1.699270 | -0.126710 |
| 11 | 1 | 0 | 2.200858 | -1.497747 | -0.449800 |

**308**

Standard orientation:

| Center Number | Atomic Number | Atomic Type | Coordinates (Angstroms) X | Y | Z |
|---|---|---|---|---|---|
| 1 | 6 | 0 | -0.908635 | 1.257462 | -0.158224 |
| 2 | 6 | 0 | 0.175321 | 0.291656 | 0.464978 |
| 3 | 1 | 0 | -1.357765 | 1.989125 | 0.504733 |
| 4 | 1 | 0 | -0.681216 | 1.583625 | -1.167402 |
| 5 | 1 | 0 | 0.386732 | 0.399856 | 1.529736 |
| 6 | 6 | 0 | -1.188268 | -0.171208 | 0.041790 |
| 7 | 8 | 0 | -1.950883 | -1.103730 | -0.058035 |
| 8 | 7 | 0 | 1.236374 | -0.054469 | -0.440284 |
| 9 | 6 | 0 | 2.386084 | -0.345047 | 0.026377 |
| 10 | 1 | 0 | 2.636660 | -0.344769 | 1.093062 |
| 11 | 1 | 0 | 3.181020 | -0.613881 | -0.663387 |

**309**

Standard orientation:

| Center Number | Atomic Number | Atomic Type | Coordinates (Angstroms) X | Y | Z |
|---|---|---|---|---|---|
| 1 | 6 | 0 | 0.692403 | 0.097676 | 0.534478 |
| 2 | 1 | 0 | 0.835439 | 0.103715 | 1.619846 |
| 3 | 6 | 0 | -0.409555 | -0.841897 | 0.079178 |
| 4 | 6 | 0 | -0.687202 | 0.587580 | 0.095348 |
| 5 | 6 | 0 | 1.947720 | 0.353677 | -0.281278 |
| 6 | 1 | 0 | 2.364448 | 1.338420 | -0.054261 |
| 7 | 1 | 0 | 2.704506 | -0.402026 | -0.058412 |
| 8 | 1 | 0 | 1.731129 | 0.312448 | -1.352148 |
| 9 | 8 | 0 | -0.713499 | -1.972016 | -0.173083 |
| 10 | 7 | 0 | -1.457883 | 1.543552 | -0.188365 |
| 11 | 1 | 0 | -0.982548 | 2.436494 | -0.018159 |

**310**

Standard orientation:

| Center Number | Atomic Number | Atomic Type | Coordinates (Angstroms) X | Y | Z |
|---|---|---|---|---|---|
| 1 | 6 | 0 | -0.189216 | 0.502325 | -0.001716 |
| 2 | 6 | 0 | 1.029218 | 1.353735 | 0.000396 |
| 3 | 6 | 0 | 1.065698 | -0.154728 | 0.001218 |
| 4 | 1 | 0 | 1.292763 | 1.880751 | 0.916771 |
| 5 | 1 | 0 | 1.297796 | 1.878877 | -0.915596 |
| 6 | 6 | 0 | 1.789303 | -1.261898 | -0.000231 |

| | | | | | |
|---|---|---|---|---|---|
| 7 | 1 | 0 | 1.308488 | -2.234314 | -0.002532 |
| 8 | 1 | 0 | 2.873991 | -1.226649 | 0.001297 |
| 9 | 8 | 0 | -1.978990 | -0.758375 | 0.000656 |
| 10 | 1 | 0 | -2.927388 | -0.595013 | 0.000863 |
| 11 | 7 | 0 | -1.454821 | 0.532392 | -0.000579 |

**400**

Standard orientation:

| Center Number | Atomic Number | Atomic Type | Coordinates (Angstroms) X | Y | Z |
|---|---|---|---|---|---|
| 1 | 6 | 0 | 0.697103 | -0.071068 | 0.000158 |
| 2 | 6 | 0 | -0.602129 | -0.478360 | -0.000255 |
| 3 | 6 | 0 | -0.294058 | 0.934736 | -0.000213 |
| 4 | 8 | 0 | -0.670213 | 2.087095 | 0.000162 |
| 5 | 6 | 0 | 2.125260 | -0.478324 | 0.000222 |
| 6 | 1 | 0 | 2.234585 | -1.564439 | 0.048935 |
| 7 | 1 | 0 | 2.622371 | -0.118443 | -0.905290 |
| 8 | 1 | 0 | 2.644402 | -0.036930 | 0.855604 |
| 9 | 7 | 0 | -1.448412 | -1.514382 | -0.000467 |
| 10 | 1 | 0 | -2.439874 | -1.353672 | 0.002214 |
| 11 | 1 | 0 | -1.117948 | -2.464511 | 0.001041 |

**401**

Standard orientation:

| Center Number | Atomic Number | Atomic Type | Coordinates (Angstroms) X | Y | Z |
|---|---|---|---|---|---|
| 1 | 6 | 0 | 0.854680 | -0.111110 | -0.000072 |
| 2 | 8 | 0 | 1.840150 | -0.828251 | 0.000046 |
| 3 | 6 | 0 | -0.536905 | -0.690668 | -0.000250 |
| 4 | 6 | 0 | -1.696315 | 0.030714 | 0.645478 |
| 5 | 6 | 0 | -1.696391 | 0.031160 | -0.645299 |
| 6 | 1 | 0 | -0.500956 | -1.780143 | -0.000602 |
| 7 | 7 | 0 | 0.937001 | 1.251246 | -0.000224 |
| 8 | 1 | 0 | 1.852139 | 1.668157 | 0.001085 |
| 9 | 1 | 0 | 0.122653 | 1.839514 | 0.000996 |
| 10 | 1 | 0 | -2.152007 | 0.288963 | 1.586868 |
| 11 | 1 | 0 | -2.152447 | 0.290220 | -1.586293 |

**402**

Standard orientation:

| Center Number | Atomic Number | Atomic Type | Coordinates (Angstroms) X | Y | Z |
|---|---|---|---|---|---|
| 1 | 6 | 0 | -1.598685 | 0.877387 | -0.019883 |
| 2 | 6 | 0 | -1.927019 | -0.594685 | 0.017138 |
| 3 | 6 | 0 | -0.583983 | 0.069462 | 0.001984 |
| 4 | 1 | 0 | -2.293897 | -1.084774 | -0.885916 |
| 5 | 1 | 0 | -2.295282 | -1.039521 | 0.942798 |
| 6 | 1 | 0 | -1.999203 | 1.877839 | -0.053982 |
| 7 | 6 | 0 | 0.873967 | -0.172871 | -0.000750 |
| 8 | 8 | 0 | 1.350541 | -1.292085 | -0.013242 |
| 9 | 7 | 0 | 1.623831 | 0.964953 | 0.006329 |
| 10 | 1 | 0 | 1.205495 | 1.877574 | 0.045556 |

| | | | | | |
|---|---|---|---|---|---|
| 11 | 1 | 0 | 2.626061 | 0.875135 | 0.022237 |

**403**

Standard orientation:

| Center Number | Atomic Number | Atomic Type | Coordinates (Angstroms) X | Y | Z |
|---|---|---|---|---|---|
| 1 | 6 | 0 | 1.395827 | -0.109857 | -0.361379 |
| 2 | 8 | 0 | 2.469364 | -0.466385 | 0.065569 |
| 3 | 1 | 0 | 1.178373 | -0.090432 | -1.455373 |
| 4 | 6 | 0 | 0.260159 | 0.351062 | 0.486136 |
| 5 | 6 | 0 | -0.754646 | 1.323753 | -0.112690 |
| 6 | 6 | 0 | -1.144798 | 0.097367 | 0.022578 |
| 7 | 1 | 0 | 0.489674 | 0.358243 | 1.556317 |
| 8 | 1 | 0 | -0.913198 | 2.357121 | -0.358474 |
| 9 | 7 | 0 | -2.028640 | -0.920647 | -0.102338 |
| 10 | 1 | 0 | -1.846976 | -1.745671 | 0.445449 |
| 11 | 1 | 0 | -3.001562 | -0.677599 | -0.203976 |

**404**

Standard orientation:

| Center Number | Atomic Number | Atomic Type | Coordinates (Angstroms) X | Y | Z |
|---|---|---|---|---|---|
| 1 | 6 | 0 | -1.704864 | 0.011376 | -0.643958 |
| 2 | 6 | 0 | -1.704916 | 0.011382 | 0.643912 |
| 3 | 6 | 0 | -0.532920 | -0.685477 | 0.000028 |
| 4 | 1 | 0 | -0.489146 | -1.777598 | 0.000058 |
| 5 | 1 | 0 | -2.141364 | 0.291292 | -1.587320 |
| 6 | 1 | 0 | -2.141483 | 0.291316 | 1.587238 |
| 7 | 6 | 0 | 0.826129 | -0.064375 | 0.000078 |
| 8 | 7 | 0 | 1.949640 | -0.658169 | -0.000025 |
| 9 | 1 | 0 | 1.819965 | -1.667794 | -0.000096 |
| 10 | 8 | 0 | 0.786676 | 1.283226 | -0.000015 |
| 11 | 1 | 0 | 1.710563 | 1.566727 | 0.000059 |

**405**

Standard orientation:

| Center Number | Atomic Number | Atomic Type | Coordinates (Angstroms) X | Y | Z |
|---|---|---|---|---|---|
| 1 | 6 | 0 | 1.616204 | -0.865397 | -0.000079 |
| 2 | 6 | 0 | 0.578569 | -0.092723 | 0.000145 |
| 3 | 6 | 0 | 1.904517 | 0.616186 | 0.000014 |
| 4 | 1 | 0 | 2.263459 | 1.092429 | -0.914765 |
| 5 | 1 | 0 | 2.263810 | 1.092308 | 0.914711 |
| 6 | 1 | 0 | 2.041460 | -1.854909 | -0.000257 |
| 7 | 6 | 0 | -0.864658 | 0.098495 | 0.000041 |
| 8 | 7 | 0 | -1.477931 | 1.211652 | -0.000072 |
| 9 | 1 | 0 | -0.810203 | 1.979388 | 0.000004 |
| 10 | 8 | 0 | -1.542732 | -1.063483 | 0.000016 |
| 11 | 1 | 0 | -2.478950 | -0.822281 | -0.000039 |

**406**

Standard orientation:

| Center Number | Atomic Number | Atomic Type | Coordinates (Angstroms) X | Y | Z |
|---|---|---|---|---|---|
| 1 | 6 | 0 | 1.671219 | 0.812672 | -0.000494 |
| 2 | 6 | 0 | 0.583568 | 0.107283 | 0.000178 |
| 3 | 6 | 0 | 1.860031 | -0.683642 | 0.000375 |
| 4 | 1 | 0 | 2.181296 | -1.183850 | 0.915439 |
| 5 | 1 | 0 | 2.181068 | -1.184961 | -0.914162 |
| 6 | 1 | 0 | 2.160867 | 1.772740 | -0.001448 |
| 7 | 6 | 0 | -0.875283 | 0.068177 | 0.000057 |
| 8 | 7 | 0 | -1.667211 | 1.063051 | 0.000278 |
| 9 | 1 | 0 | -1.144855 | 1.935945 | 0.000609 |
| 10 | 8 | 0 | -1.353551 | -1.188037 | -0.000340 |
| 11 | 1 | 0 | -2.316704 | -1.103875 | -0.000363 |

**407**

Standard orientation:

| Center Number | Atomic Number | Atomic Type | Coordinates (Angstroms) X | Y | Z |
|---|---|---|---|---|---|
| 1 | 6 | 0 | -1.709333 | 0.022208 | -0.644268 |
| 2 | 6 | 0 | -1.709331 | 0.022218 | 0.644265 |
| 3 | 6 | 0 | -0.545550 | -0.688931 | 0.000004 |
| 4 | 1 | 0 | -0.494251 | -1.777315 | 0.000015 |
| 5 | 1 | 0 | -2.151744 | 0.299031 | -1.586052 |
| 6 | 1 | 0 | -2.151740 | 0.299055 | 1.586046 |
| 7 | 6 | 0 | 0.820951 | -0.090160 | 0.000004 |
| 8 | 7 | 0 | 1.848543 | -0.837022 | 0.000000 |
| 9 | 1 | 0 | 2.719884 | -0.303944 | -0.000008 |
| 10 | 8 | 0 | 0.788345 | 1.268319 | -0.000003 |
| 11 | 1 | 0 | 1.690870 | 1.603767 | -0.000005 |

**408**

Standard orientation:

| Center Number | Atomic Number | Atomic Type | Coordinates (Angstroms) X | Y | Z |
|---|---|---|---|---|---|
| 1 | 6 | 0 | -1.628974 | 0.837043 | 0.000133 |
| 2 | 6 | 0 | -0.591918 | 0.061214 | -0.000088 |
| 3 | 6 | 0 | -1.912107 | -0.645364 | -0.000099 |
| 4 | 1 | 0 | -2.262875 | -1.127203 | -0.914544 |
| 5 | 1 | 0 | -2.262610 | -1.127857 | 0.914093 |
| 6 | 1 | 0 | -2.048793 | 1.829209 | 0.000725 |
| 7 | 6 | 0 | 0.851515 | -0.108279 | 0.000048 |
| 8 | 7 | 0 | 1.365628 | -1.270696 | 0.000116 |
| 9 | 1 | 0 | 2.386740 | -1.239255 | 0.000004 |
| 10 | 8 | 0 | 1.484914 | 1.092757 | -0.000166 |
| 11 | 1 | 0 | 2.437744 | 0.950234 | 0.000278 |

**409**

Standard orientation:

| Center | Atomic | Atomic | Coordinates (Angstroms) |
|---|---|---|---|

| Number | Number | Type | X | Y | Z |
|---|---|---|---|---|---|
| 1 | 6 | 0 | 1.675941 | 0.831660 | -0.000778 |
| 2 | 6 | 0 | 0.590877 | 0.126687 | 0.000288 |
| 3 | 6 | 0 | 1.867324 | -0.665036 | 0.000601 |
| 4 | 1 | 0 | 2.191452 | -1.166078 | 0.915022 |
| 5 | 1 | 0 | 2.191048 | -1.167976 | -0.912928 |
| 6 | 1 | 0 | 2.154669 | 1.796518 | -0.002429 |
| 7 | 6 | 0 | -0.865089 | 0.082488 | 0.000115 |
| 8 | 7 | 0 | -1.546163 | 1.155624 | 0.000563 |
| 9 | 1 | 0 | -2.551534 | 0.974903 | 0.000270 |
| 10 | 8 | 0 | -1.312595 | -1.197973 | -0.000542 |
| 11 | 1 | 0 | -2.276049 | -1.197743 | -0.000894 |

**410**

Standard orientation:

| Center Number | Atomic Number | Atomic Type | Coordinates (Angstroms) X | Y | Z |
|---|---|---|---|---|---|
| 1 | 6 | 0 | 0.638728 | -1.183382 | 0.642563 |
| 2 | 6 | 0 | 0.639388 | -1.183369 | -0.642399 |
| 3 | 6 | 0 | 0.365773 | 0.154430 | -0.000145 |
| 4 | 1 | 0 | 0.735217 | -1.677499 | 1.593779 |
| 5 | 1 | 0 | 0.736579 | -1.677733 | -1.593430 |
| 6 | 7 | 0 | 1.397883 | 1.162763 | 0.000304 |
| 7 | 1 | 0 | 1.334045 | 1.757509 | -0.819202 |
| 8 | 1 | 0 | 1.332358 | 1.758548 | 0.818919 |
| 9 | 6 | 0 | -1.059986 | 0.639494 | -0.000277 |
| 10 | 1 | 0 | -1.165444 | 1.749891 | -0.000140 |
| 11 | 8 | 0 | -2.032670 | -0.076637 | -0.000062 |

**411**

Standard orientation:

| Center Number | Atomic Number | Atomic Type | Coordinates (Angstroms) X | Y | Z |
|---|---|---|---|---|---|
| 1 | 6 | 0 | -2.132155 | -0.589141 | 0.000307 |
| 2 | 6 | 0 | -0.873956 | -0.293090 | 0.000197 |
| 3 | 6 | 0 | -1.805507 | 0.894656 | -0.000245 |
| 4 | 1 | 0 | -1.950296 | 1.478340 | -0.914600 |
| 5 | 1 | 0 | -2.932329 | -1.309363 | -0.000376 |
| 6 | 1 | 0 | -1.950028 | 1.478870 | 0.913805 |
| 7 | 7 | 0 | 0.438764 | -0.700691 | -0.000212 |
| 8 | 6 | 0 | 1.289925 | 0.242409 | 0.000192 |
| 9 | 1 | 0 | 1.021765 | 1.306620 | 0.000960 |
| 10 | 8 | 0 | 2.595585 | -0.043714 | -0.000176 |
| 11 | 1 | 0 | 3.105024 | 0.771088 | 0.000402 |

**412**

Standard orientation:

| Center Number | Atomic Number | Atomic Type | Coordinates (Angstroms) X | Y | Z |
|---|---|---|---|---|---|
| 1 | 6 | 0 | -1.932075 | -0.348251 | -0.436516 |
| 2 | 6 | 0 | -0.728497 | -0.368437 | 0.040781 |

| | | | | | |
|---|---|---|---|---|---|
| 3 | 6 | 0 | -1.519957 | 0.873044 | 0.358862 |
| 4 | 1 | 0 | -1.293629 | 1.813742 | -0.151319 |
| 5 | 1 | 0 | -2.704376 | -0.780560 | -1.049859 |
| 6 | 1 | 0 | -1.904679 | 1.010591 | 1.373246 |
| 7 | 7 | 0 | 0.453300 | -1.042534 | 0.236588 |
| 8 | 6 | 0 | 1.554894 | -0.432647 | 0.114426 |
| 9 | 1 | 0 | 2.485208 | -0.959306 | 0.329522 |
| 10 | 8 | 0 | 1.674636 | 0.845986 | -0.291011 |
| 11 | 1 | 0 | 2.601100 | 1.103131 | -0.294934 |

**413**

Standard orientation:

| Center Number | Atomic Number | Atomic Type | Coordinates (Angstroms) X | Y | Z |
|---|---|---|---|---|---|
| 1 | 6 | 0 | 1.810944 | 0.851238 | -0.126670 |
| 2 | 6 | 0 | 0.861217 | -0.033592 | -0.117592 |
| 3 | 6 | 0 | 2.215437 | -0.566041 | 0.235391 |
| 4 | 1 | 0 | 2.768280 | -1.174709 | -0.485683 |
| 5 | 1 | 0 | 2.173504 | 1.838983 | -0.355942 |
| 6 | 1 | 0 | 2.434744 | -0.820079 | 1.276344 |
| 7 | 7 | 0 | -0.427585 | -0.423045 | -0.375726 |
| 8 | 6 | 0 | -1.362779 | 0.195131 | 0.209306 |
| 9 | 1 | 0 | -1.204544 | 0.991201 | 0.947753 |
| 10 | 8 | 0 | -2.638288 | -0.120673 | -0.055930 |
| 11 | 1 | 0 | -3.221497 | 0.410880 | 0.492448 |

**414**

Standard orientation:

| Center Number | Atomic Number | Atomic Type | Coordinates (Angstroms) X | Y | Z |
|---|---|---|---|---|---|
| 1 | 6 | 0 | -0.233153 | -0.748741 | 0.008034 |
| 2 | 6 | 0 | 0.748175 | 0.139254 | -0.000638 |
| 3 | 7 | 0 | 2.064824 | 0.479103 | -0.076897 |
| 4 | 1 | 0 | 2.290759 | 1.399558 | 0.266324 |
| 5 | 1 | 0 | 2.721081 | -0.233480 | 0.203148 |
| 6 | 8 | 0 | -0.572061 | -2.043843 | 0.001936 |
| 7 | 1 | 0 | -1.532363 | -2.099322 | -0.003371 |
| 8 | 6 | 0 | -0.616407 | 0.624268 | 0.000930 |
| 9 | 6 | 0 | -1.501616 | 1.626114 | 0.001786 |
| 10 | 1 | 0 | -1.172606 | 2.657516 | -0.017429 |
| 11 | 1 | 0 | -2.566143 | 1.427374 | 0.013454 |

**415**

Standard orientation:

| Center Number | Atomic Number | Atomic Type | Coordinates (Angstroms) X | Y | Z |
|---|---|---|---|---|---|
| 1 | 6 | 0 | 1.056952 | 1.305516 | -0.000226 |
| 2 | 6 | 0 | -0.035398 | 0.541778 | 0.000031 |
| 3 | 1 | 0 | 1.449040 | 2.312737 | -0.000411 |
| 4 | 6 | 0 | -1.514197 | 0.471326 | 0.000316 |
| 5 | 1 | 0 | -1.884051 | 1.011141 | 0.886215 |
| 6 | 1 | 0 | -1.884517 | 1.012138 | -0.884776 |

| | | | | | |
|---|---|---|---|---|---|
| 7 | 8 | 0 | -1.900951 | -0.881631 | -0.000327 |
| 8 | 1 | 0 | -2.860560 | -0.919015 | -0.000145 |
| 9 | 6 | 0 | 1.251343 | -0.108551 | 0.000014 |
| 10 | 7 | 0 | 2.046792 | -1.094732 | 0.000087 |
| 11 | 1 | 0 | 1.507945 | -1.961240 | 0.000310 |

**416**

Standard orientation:

| Center | Atomic | Atomic | Coordinates (Angstroms) | | |
|---|---|---|---|---|---|
| Number | Number | Type | X | Y | Z |
| 1 | 6 | 0 | 0.485094 | 1.186485 | -0.000013 |
| 2 | 6 | 0 | -0.065320 | -0.030260 | 0.000023 |
| 3 | 1 | 0 | 0.357423 | 2.258256 | 0.000064 |
| 4 | 8 | 0 | -1.163380 | -0.754302 | 0.000004 |
| 5 | 6 | 0 | -2.369145 | 0.016710 | -0.000009 |
| 6 | 1 | 0 | -2.418934 | 0.644274 | -0.894842 |
| 7 | 1 | 0 | -3.188144 | -0.700314 | -0.000323 |
| 8 | 1 | 0 | -2.419264 | 0.643801 | 0.895142 |
| 9 | 6 | 0 | 1.368388 | 0.046530 | -0.000019 |
| 10 | 7 | 0 | 2.558495 | -0.388610 | 0.000005 |
| 11 | 1 | 0 | 2.552391 | -1.408129 | -0.000004 |

**417**

Standard orientation:

| Center | Atomic | Atomic | Coordinates (Angstroms) | | |
|---|---|---|---|---|---|
| Number | Number | Type | X | Y | Z |
| 1 | 6 | 0 | 1.035136 | 1.305043 | -0.000889 |
| 2 | 6 | 0 | -0.039412 | 0.515455 | 0.000144 |
| 3 | 1 | 0 | 1.399285 | 2.323073 | -0.001674 |
| 4 | 6 | 0 | -1.520156 | 0.453910 | 0.001216 |
| 5 | 1 | 0 | -1.877718 | 1.002189 | 0.887623 |
| 6 | 1 | 0 | -1.879347 | 1.006025 | -0.882133 |
| 7 | 8 | 0 | -1.932565 | -0.887388 | -0.001278 |
| 8 | 1 | 0 | -2.892653 | -0.905206 | -0.000312 |
| 9 | 6 | 0 | 1.248232 | -0.115221 | 0.000044 |
| 10 | 7 | 0 | 1.921542 | -1.188207 | 0.000537 |
| 11 | 1 | 0 | 2.917361 | -0.964646 | -0.000122 |

**418**

Standard orientation:

| Center | Atomic | Atomic | Coordinates (Angstroms) | | |
|---|---|---|---|---|---|
| Number | Number | Type | X | Y | Z |
| 1 | 6 | 0 | 1.995401 | -0.100655 | 0.644214 |
| 2 | 6 | 0 | 1.995347 | -0.100783 | -0.644256 |
| 3 | 6 | 0 | 0.644745 | -0.344907 | 0.000061 |
| 4 | 1 | 0 | 0.226153 | -1.348783 | 0.000175 |
| 5 | 1 | 0 | 2.510201 | -0.011734 | 1.586200 |
| 6 | 1 | 0 | 2.510070 | -0.012054 | -1.586302 |
| 7 | 6 | 0 | -0.361658 | 0.743289 | 0.000019 |
| 8 | 1 | 0 | 0.002835 | 1.770176 | -0.000222 |
| 9 | 7 | 0 | -1.633910 | 0.644494 | -0.000044 |
| 10 | 8 | 0 | -2.055348 | -0.686698 | 0.000036 |

| | | | | | |
|---|---|---|---|---|---|
| 11 | 1 | 0 | -3.012118 | -0.597136 | -0.000060 |

**419**

Standard orientation:

| Center Number | Atomic Number | Atomic Type | Coordinates (Angstroms) X | Y | Z |
|---|---|---|---|---|---|
| 1 | 6 | 0 | -1.906664 | 0.657980 | 0.071442 |
| 2 | 6 | 0 | -1.899060 | -0.633748 | 0.087401 |
| 3 | 6 | 0 | -0.646737 | 0.012339 | -0.446793 |
| 4 | 1 | 0 | -2.415460 | 1.592062 | 0.242367 |
| 5 | 1 | 0 | -2.395269 | -1.570059 | 0.280999 |
| 6 | 1 | 0 | -0.436095 | -0.000771 | -1.518365 |
| 7 | 6 | 0 | 0.601778 | 0.027487 | 0.374267 |
| 8 | 1 | 0 | 0.444236 | 0.069889 | 1.456991 |
| 9 | 8 | 0 | 1.661613 | -0.780155 | -0.040967 |
| 10 | 7 | 0 | 1.751028 | 0.686211 | -0.178959 |
| 11 | 1 | 0 | 2.356579 | 0.962296 | 0.600558 |

**420**

Standard orientation:

| Center Number | Atomic Number | Atomic Type | Coordinates (Angstroms) X | Y | Z |
|---|---|---|---|---|---|
| 1 | 6 | 0 | 1.871493 | 0.653020 | -0.076377 |
| 2 | 6 | 0 | 1.848901 | -0.641050 | -0.047826 |
| 3 | 6 | 0 | 0.599751 | 0.034937 | 0.398394 |
| 4 | 1 | 0 | 2.384151 | 1.576417 | -0.288935 |
| 5 | 1 | 0 | 2.328676 | -1.590877 | -0.213017 |
| 6 | 1 | 0 | 0.308469 | 0.046254 | 1.454956 |
| 7 | 8 | 0 | -1.605890 | -0.794323 | 0.052364 |
| 8 | 7 | 0 | -0.529914 | 0.050686 | -0.522625 |
| 9 | 6 | 0 | -1.715854 | 0.595404 | 0.045507 |
| 10 | 1 | 0 | -2.431389 | 1.028739 | -0.652979 |
| 11 | 1 | 0 | -1.659129 | 1.085377 | 1.021250 |

**500**

Standard orientation:

| Center Number | Atomic Number | Atomic Type | Coordinates (Angstroms) X | Y | Z |
|---|---|---|---|---|---|
| 1 | 6 | 0 | -0.200756 | 1.224894 | 0.437886 |
| 2 | 6 | 0 | 1.118156 | 1.070074 | -0.288862 |
| 3 | 6 | 0 | 0.502221 | -0.283213 | -0.239742 |
| 4 | 1 | 0 | 2.024665 | 1.193012 | 0.314806 |
| 5 | 1 | 0 | 1.228996 | 1.551963 | -1.262769 |
| 6 | 1 | 0 | -0.367637 | 1.742344 | 1.376308 |
| 7 | 6 | 0 | -0.905399 | 0.117560 | -0.115801 |
| 8 | 8 | 0 | -1.990323 | -0.433992 | -0.122081 |
| 9 | 7 | 0 | 1.065829 | -1.445296 | 0.133718 |
| 10 | 1 | 0 | 0.458189 | -2.189127 | 0.439901 |
| 11 | 1 | 0 | 2.032244 | -1.485067 | 0.411490 |

**501**

Standard orientation:

| Center Number | Atomic Number | Atomic Type | Coordinates (Angstroms) X | Y | Z |
|---|---|---|---|---|---|
| 1 | 6 | 0 | 1.092480 | -0.081940 | -0.068394 |
| 2 | 6 | 0 | -0.345580 | 0.059555 | -0.041972 |
| 3 | 6 | 0 | -1.626639 | -0.174267 | -0.739542 |
| 4 | 6 | 0 | -1.515481 | -0.341912 | 0.774463 |
| 5 | 1 | 0 | -1.718046 | -1.063448 | -1.354377 |
| 6 | 1 | 0 | -2.158414 | 0.699186 | -1.103501 |
| 7 | 7 | 0 | 0.601040 | 1.211848 | -0.043698 |
| 8 | 1 | 0 | 0.728119 | 1.782444 | 0.788571 |
| 9 | 8 | 0 | 2.103516 | -0.718291 | -0.019449 |
| 10 | 1 | 0 | -1.531417 | -1.343246 | 1.192889 |
| 11 | 1 | 0 | -1.984330 | 0.419844 | 1.390562 |

**502**

Standard orientation:

| Center Number | Atomic Number | Atomic Type | Coordinates (Angstroms) X | Y | Z |
|---|---|---|---|---|---|
| 1 | 6 | 0 | -1.578770 | -0.386175 | 0.657041 |
| 2 | 1 | 0 | -1.619175 | -1.394538 | 1.057006 |
| 3 | 1 | 0 | -2.172716 | 0.355616 | 1.186200 |
| 4 | 6 | 0 | -0.346608 | 0.082256 | 0.002617 |
| 5 | 6 | 0 | 0.569804 | 1.306405 | 0.102830 |
| 6 | 6 | 0 | 1.087247 | -0.104095 | 0.001082 |
| 7 | 1 | 0 | 0.632285 | 1.847925 | 1.045486 |
| 8 | 1 | 0 | 0.681034 | 1.927273 | -0.785735 |
| 9 | 7 | 0 | -1.479322 | -0.305748 | -0.809344 |
| 10 | 1 | 0 | -1.951270 | 0.534497 | -1.139965 |
| 11 | 8 | 0 | 2.049382 | -0.815110 | -0.034876 |

**503**

Standard orientation:

| Center Number | Atomic Number | Atomic Type | Coordinates (Angstroms) X | Y | Z |
|---|---|---|---|---|---|
| 1 | 6 | 0 | -0.914005 | 0.073069 | -0.476763 |
| 2 | 6 | 0 | 0.457056 | 0.147383 | 0.025952 |
| 3 | 6 | 0 | 1.563817 | -0.639418 | 0.461084 |
| 4 | 1 | 0 | 2.043504 | -1.335404 | 1.137314 |
| 5 | 8 | 0 | -1.749121 | -0.893120 | 0.079742 |
| 6 | 1 | 0 | -2.640539 | -0.533284 | 0.098040 |
| 7 | 6 | 0 | -0.438883 | 1.264176 | 0.393634 |
| 8 | 1 | 0 | -0.317936 | 2.227617 | -0.094819 |
| 9 | 7 | 0 | 1.827667 | 0.017841 | -0.583030 |
| 10 | 1 | 0 | -0.814555 | 1.300603 | 1.413067 |
| 11 | 1 | 0 | -1.079084 | 0.289288 | -1.533775 |

**504**

Standard orientation:

| Center | Atomic | Atomic | Coordinates (Angstroms) |
|---|---|---|---|

| Number | Number | Type | X | Y | Z |
|---|---|---|---|---|---|
| 1 | 6 | 0 | -1.000541 | 0.075141 | 0.033483 |
| 2 | 6 | 0 | 0.445121 | 0.192313 | 0.024262 |
| 3 | 6 | 0 | 1.664639 | -0.285558 | 0.677884 |
| 4 | 1 | 0 | 2.377851 | 0.412659 | 1.108769 |
| 5 | 8 | 0 | -2.005224 | -0.798794 | 0.042021 |
| 6 | 1 | 0 | -2.836626 | -0.312803 | -0.002888 |
| 7 | 6 | 0 | -0.553076 | 1.305566 | -0.002925 |
| 8 | 7 | 0 | 1.546386 | -0.200290 | -0.817792 |
| 9 | 1 | 0 | -0.719098 | 2.368446 | -0.046322 |
| 10 | 1 | 0 | 1.398010 | -1.129412 | -1.206807 |
| 11 | 1 | 0 | 1.660100 | -1.271278 | 1.139392 |

**505**

Standard orientation:

| Center | Atomic | Atomic | Coordinates (Angstroms) | | |
|---|---|---|---|---|---|
| Number | Number | Type | X | Y | Z |
| 1 | 8 | 0 | -1.498501 | -0.408027 | 0.788281 |
| 2 | 7 | 0 | -1.587533 | -0.463392 | -0.721393 |
| 3 | 1 | 0 | -2.275055 | 0.267806 | -0.931635 |
| 4 | 6 | 0 | -0.520509 | 0.147303 | -0.012955 |
| 5 | 6 | 0 | 0.309068 | 1.334294 | 0.018696 |
| 6 | 6 | 0 | 0.929912 | 0.167394 | -0.024078 |
| 7 | 6 | 0 | 2.190998 | -0.602834 | -0.031632 |
| 8 | 1 | 0 | 2.236732 | -1.234992 | -0.922971 |
| 9 | 1 | 0 | 3.062586 | 0.054586 | -0.012231 |
| 10 | 1 | 0 | 0.396339 | 2.409754 | 0.072726 |
| 11 | 1 | 0 | 2.223319 | -1.266142 | 0.837426 |

**506**

Standard orientation:

| Center | Atomic | Atomic | Coordinates (Angstroms) | | |
|---|---|---|---|---|---|
| Number | Number | Type | X | Y | Z |
| 1 | 8 | 0 | 1.436925 | -0.427434 | -0.775896 |
| 2 | 7 | 0 | 1.527411 | -0.459226 | 0.717868 |
| 3 | 1 | 0 | 2.161494 | 0.321660 | 0.920203 |
| 4 | 6 | 0 | 0.428529 | 0.084507 | 0.006591 |
| 5 | 6 | 0 | -0.362267 | 1.359284 | -0.028102 |
| 6 | 6 | 0 | -1.006569 | -0.000349 | 0.028073 |
| 7 | 1 | 0 | -0.408082 | 1.907031 | -0.967783 |
| 8 | 1 | 0 | -0.382884 | 1.984076 | 0.863668 |
| 9 | 6 | 0 | -2.121593 | -0.704545 | 0.039611 |
| 10 | 1 | 0 | -2.091989 | -1.789166 | 0.061490 |
| 11 | 1 | 0 | -3.094423 | -0.222930 | 0.027472 |

**600**

Standard orientation:

| Center | Atomic | Atomic | Coordinates (Angstroms) | | |
|---|---|---|---|---|---|
| Number | Number | Type | X | Y | Z |
| 1 | 6 | 0 | -0.981091 | -0.068194 | 0.000150 |
| 2 | 8 | 0 | -2.061076 | -0.606977 | -0.001575 |

| | | | | | |
|---|---|---|---|---|---|
| 3 | 6 | 0 | 0.272365 | -0.971695 | 0.001500 |
| 4 | 1 | 0 | 0.209040 | -1.620348 | 0.880734 |
| 5 | 1 | 0 | 0.208598 | -1.623790 | -0.875118 |
| 6 | 6 | 0 | -0.791364 | 1.424303 | 0.000706 |
| 7 | 1 | 0 | -0.213512 | 1.731787 | 0.878433 |
| 8 | 1 | 0 | -0.212261 | 1.732109 | -0.876084 |
| 9 | 1 | 0 | -1.764687 | 1.913256 | 0.000033 |
| 10 | 6 | 0 | 1.555368 | -0.275992 | -0.000053 |
| 11 | 7 | 0 | 2.561394 | 0.296039 | -0.001316 |

**601**

Standard orientation:

| Center Number | Atomic Number | Atomic Type | Coordinates (Angstroms) X | Y | Z |
|---|---|---|---|---|---|
| 1 | 6 | 0 | -1.888254 | 0.434126 | -0.000218 |
| 2 | 8 | 0 | -2.325547 | -0.690768 | 0.000067 |
| 3 | 1 | 0 | -2.566608 | 1.312194 | -0.000192 |
| 4 | 6 | 0 | -0.415788 | 0.761883 | 0.000078 |
| 5 | 1 | 0 | -0.202876 | 1.389068 | 0.874665 |
| 6 | 1 | 0 | -0.202092 | 1.389932 | -0.873565 |
| 7 | 6 | 0 | 0.448697 | -0.501683 | -0.000041 |
| 8 | 1 | 0 | 0.219380 | -1.114883 | -0.876046 |
| 9 | 1 | 0 | 0.219251 | -1.115390 | 0.875593 |
| 10 | 6 | 0 | 1.876283 | -0.183818 | 0.000084 |
| 11 | 7 | 0 | 3.001671 | 0.086025 | -0.000059 |

**602**

Standard orientation:

| Center Number | Atomic Number | Atomic Type | Coordinates (Angstroms) X | Y | Z |
|---|---|---|---|---|---|
| 1 | 6 | 0 | -0.788691 | -0.096356 | 0.000072 |
| 2 | 6 | 0 | 0.325247 | -0.852339 | 0.000204 |
| 3 | 1 | 0 | 0.246555 | -1.932932 | 0.000317 |
| 4 | 6 | 0 | 1.622593 | -0.271871 | 0.000117 |
| 5 | 7 | 0 | 2.681657 | 0.205236 | -0.000245 |
| 6 | 8 | 0 | -1.979821 | -0.730381 | -0.000161 |
| 7 | 1 | 0 | -2.694082 | -0.087323 | -0.000552 |
| 8 | 6 | 0 | -0.820828 | 1.400022 | 0.000075 |
| 9 | 1 | 0 | -1.350264 | 1.765138 | 0.887174 |
| 10 | 1 | 0 | 0.184958 | 1.819475 | 0.000235 |
| 11 | 1 | 0 | -1.350120 | 1.765304 | -0.886981 |

**603**

Standard orientation:

| Center Number | Atomic Number | Atomic Type | Coordinates (Angstroms) X | Y | Z |
|---|---|---|---|---|---|
| 1 | 6 | 0 | 1.335110 | -0.214883 | 0.158067 |
| 2 | 8 | 0 | 1.456952 | -1.327981 | -0.274535 |
| 3 | 1 | 0 | 2.213438 | 0.425896 | 0.386213 |
| 4 | 6 | 0 | 0.004961 | 0.479385 | 0.445314 |
| 5 | 1 | 0 | -0.011247 | 0.679449 | 1.525799 |
| 6 | 6 | 0 | -0.066493 | 1.824895 | -0.302262 |

| | | | | | |
|---|---|---|---|---|---|
| 7 | 1 | 0 | -0.973955 | 2.364673 | -0.027818 |
| 8 | 1 | 0 | 0.795957 | 2.443500 | -0.039570 |
| 9 | 1 | 0 | -0.068942 | 1.665916 | -1.382707 |
| 10 | 6 | 0 | -1.137197 | -0.381320 | 0.130228 |
| 11 | 7 | 0 | -2.061307 | -1.029148 | -0.121959 |

**604**

Standard orientation:

| Center | Atomic | Atomic | Coordinates (Angstroms) | | |
|---|---|---|---|---|---|
| Number | Number | Type | X | Y | Z |
| 1 | 6 | 0 | 0.199698 | 0.439986 | -0.000001 |
| 2 | 6 | 0 | -1.140566 | 0.496048 | -0.000005 |
| 3 | 1 | 0 | -1.645615 | 1.460089 | -0.000029 |
| 4 | 6 | 0 | 0.856569 | -0.832580 | -0.000001 |
| 5 | 7 | 0 | 1.430388 | -1.841064 | -0.000005 |
| 6 | 6 | 0 | 1.065877 | 1.673743 | 0.000003 |
| 7 | 1 | 0 | 1.711218 | 1.700211 | 0.882985 |
| 8 | 1 | 0 | 1.711217 | 1.700225 | -0.882979 |
| 9 | 1 | 0 | 0.450341 | 2.577019 | 0.000010 |
| 10 | 8 | 0 | -1.911299 | -0.606431 | 0.000018 |
| 11 | 1 | 0 | -2.838945 | -0.361835 | -0.000078 |

**605**

Standard orientation:

| Center | Atomic | Atomic | Coordinates (Angstroms) | | |
|---|---|---|---|---|---|
| Number | Number | Type | X | Y | Z |
| 1 | 6 | 0 | -0.102448 | 0.177506 | -0.000006 |
| 2 | 6 | 0 | 0.852445 | -0.765785 | -0.000012 |
| 3 | 1 | 0 | 0.597807 | -1.822722 | -0.000005 |
| 4 | 6 | 0 | -1.461210 | -0.272068 | -0.000005 |
| 5 | 7 | 0 | -2.571347 | -0.611589 | 0.000008 |
| 6 | 8 | 0 | 2.161508 | -0.445073 | -0.000004 |
| 7 | 1 | 0 | 2.693952 | -1.243317 | 0.000086 |
| 8 | 6 | 0 | 0.165193 | 1.659486 | 0.000009 |
| 9 | 1 | 0 | -0.273986 | 2.132431 | 0.883087 |
| 10 | 1 | 0 | 1.239154 | 1.848138 | 0.000299 |
| 11 | 1 | 0 | -0.273450 | 2.132340 | -0.883400 |

**606**

Standard orientation:

| Center | Atomic | Atomic | Coordinates (Angstroms) | | |
|---|---|---|---|---|---|
| Number | Number | Type | X | Y | Z |
| 1 | 6 | 0 | 1.847958 | -0.788030 | 0.468029 |
| 2 | 6 | 0 | 0.899867 | -0.025765 | -0.074333 |
| 3 | 1 | 0 | 2.748747 | -0.360598 | 0.896792 |
| 4 | 1 | 0 | 1.739279 | -1.863809 | 0.500517 |
| 5 | 6 | 0 | -0.355094 | -0.574689 | -0.707315 |
| 6 | 1 | 0 | -0.425090 | -0.216098 | -1.740192 |
| 7 | 1 | 0 | -0.318863 | -1.665902 | -0.731212 |
| 8 | 8 | 0 | 0.918807 | 1.326603 | -0.159062 |
| 9 | 1 | 0 | 1.694135 | 1.665873 | 0.298679 |
| 10 | 6 | 0 | -1.569364 | -0.157376 | 0.002113 |

| | | | | | |
|---|---|---|---|---|---|
| 11 | 7 | 0 | -2.532696 | 0.157553 | 0.559564 |

**607**

Standard orientation:

| Center Number | Atomic Number | Atomic Type | Coordinates (Angstroms) X | Y | Z |
|---|---|---|---|---|---|
| 1 | 6 | 0 | -0.839573 | -0.720606 | -0.000032 |
| 2 | 6 | 0 | 0.139405 | 0.193517 | 0.000008 |
| 3 | 1 | 0 | -0.531901 | -1.760454 | -0.000042 |
| 4 | 6 | 0 | 1.518722 | -0.225450 | -0.000008 |
| 5 | 7 | 0 | 2.619484 | -0.585847 | 0.000015 |
| 6 | 8 | 0 | 0.022954 | 1.545983 | -0.000004 |
| 7 | 1 | 0 | -0.909131 | 1.783626 | -0.000022 |
| 8 | 6 | 0 | -2.310275 | -0.439995 | 0.000011 |
| 9 | 1 | 0 | -2.786592 | -0.881348 | -0.881255 |
| 10 | 1 | 0 | -2.786363 | -0.880355 | 0.881922 |
| 11 | 1 | 0 | -2.555715 | 0.626796 | -0.000550 |

**608**

Standard orientation:

| Center Number | Atomic Number | Atomic Type | Coordinates (Angstroms) X | Y | Z |
|---|---|---|---|---|---|
| 1 | 6 | 0 | 1.091761 | 0.577244 | 0.000025 |
| 2 | 6 | 0 | -0.245725 | 0.520098 | -0.000169 |
| 3 | 1 | 0 | 1.550353 | 1.565329 | 0.000222 |
| 4 | 6 | 0 | -0.946521 | -0.739981 | -0.000086 |
| 5 | 7 | 0 | -1.482945 | -1.766681 | 0.000074 |
| 6 | 8 | 0 | -1.105508 | 1.573551 | 0.000092 |
| 7 | 1 | 0 | -0.596860 | 2.390418 | -0.000187 |
| 8 | 6 | 0 | 2.013649 | -0.601549 | 0.000024 |
| 9 | 1 | 0 | 1.466061 | -1.546480 | 0.000719 |
| 10 | 1 | 0 | 2.662537 | -0.583271 | -0.881774 |
| 11 | 1 | 0 | 2.663606 | -0.582507 | 0.881002 |

**609**

Standard orientation:

| Center Number | Atomic Number | Atomic Type | Coordinates (Angstroms) X | Y | Z |
|---|---|---|---|---|---|
| 1 | 6 | 0 | 2.202842 | -0.672790 | 0.157535 |
| 2 | 6 | 0 | 1.137758 | -0.150930 | -0.436514 |
| 3 | 1 | 0 | 2.275865 | -0.749899 | 1.239063 |
| 4 | 1 | 0 | 3.042595 | -1.045436 | -0.418661 |
| 5 | 1 | 0 | 1.066073 | -0.072379 | -1.517887 |
| 6 | 6 | 0 | -0.057998 | 0.369894 | 0.317286 |
| 7 | 1 | 0 | 0.149336 | 0.336423 | 1.397094 |
| 8 | 8 | 0 | -0.317212 | 1.680470 | -0.129332 |
| 9 | 1 | 0 | -1.103899 | 2.010248 | 0.316235 |
| 10 | 6 | 0 | -1.233323 | -0.507411 | 0.077351 |
| 11 | 7 | 0 | -2.169707 | -1.165043 | -0.096448 |

**610**

Standard orientation:

| Center Number | Atomic Number | Atomic Type | Coordinates (Angstroms) X | Y | Z |
|---|---|---|---|---|---|
| 1 | 6 | 0 | 1.709756 | 0.601834 | -0.000113 |
| 2 | 6 | 0 | 0.384876 | 0.712569 | 0.000442 |
| 3 | 1 | 0 | 2.358877 | 1.473701 | -0.000320 |
| 4 | 1 | 0 | -0.063678 | 1.697562 | 0.000721 |
| 5 | 8 | 0 | 2.310926 | -0.618742 | -0.000362 |
| 6 | 1 | 0 | 3.264149 | -0.514557 | -0.001289 |
| 7 | 6 | 0 | -0.506816 | -0.508209 | 0.000766 |
| 8 | 1 | 0 | -0.304943 | -1.135769 | 0.876858 |
| 9 | 1 | 0 | -0.304143 | -1.136781 | -0.874413 |
| 10 | 6 | 0 | -1.930324 | -0.168329 | -0.000056 |
| 11 | 7 | 0 | -3.054661 | 0.105513 | -0.000700 |

**611**

Standard orientation:

| Center Number | Atomic Number | Atomic Type | Coordinates (Angstroms) X | Y | Z |
|---|---|---|---|---|---|
| 1 | 6 | 0 | 1.522936 | 0.124913 | -0.306177 |
| 2 | 6 | 0 | 0.444556 | 0.164115 | 0.471597 |
| 3 | 1 | 0 | 1.530069 | 0.589497 | -1.292107 |
| 4 | 1 | 0 | 0.463647 | -0.316869 | 1.444046 |
| 5 | 6 | 0 | -0.820434 | 0.862453 | 0.047983 |
| 6 | 1 | 0 | -1.098038 | 1.645189 | 0.763682 |
| 7 | 1 | 0 | -0.689340 | 1.353847 | -0.922135 |
| 8 | 6 | 0 | -1.961420 | -0.057853 | -0.061521 |
| 9 | 7 | 0 | -2.855416 | -0.788697 | -0.139089 |
| 10 | 8 | 0 | 2.668796 | -0.484168 | 0.085971 |
| 11 | 1 | 0 | 3.317374 | -0.439211 | -0.618929 |

**612**

Standard orientation:

| Center Number | Atomic Number | Atomic Type | Coordinates (Angstroms) X | Y | Z |
|---|---|---|---|---|---|
| 1 | 6 | 0 | -0.444687 | 1.873169 | -0.192025 |
| 2 | 6 | 0 | -0.145325 | 0.600881 | 0.073865 |
| 3 | 1 | 0 | -1.456932 | 2.187251 | -0.419926 |
| 4 | 1 | 0 | 0.329658 | 2.633245 | -0.182230 |
| 5 | 6 | 0 | -1.163877 | -0.416829 | 0.045240 |
| 6 | 7 | 0 | -1.924287 | -1.292306 | 0.037706 |
| 7 | 6 | 0 | 1.242836 | 0.092949 | 0.421687 |
| 8 | 1 | 0 | 1.258223 | -0.201108 | 1.481876 |
| 9 | 1 | 0 | 1.958305 | 0.908583 | 0.293546 |
| 10 | 8 | 0 | 1.663533 | -0.955715 | -0.414499 |
| 11 | 1 | 0 | 1.138812 | -1.737135 | -0.213818 |

**613**

Standard orientation:

| Center | Atomic | Atomic | Coordinates (Angstroms) |
|---|---|---|---|

| Number | Number | Type | X | Y | Z |
|---|---|---|---|---|---|
| 1 | 6 | 0 | -0.869793 | 1.039594 | -0.046035 |
| 2 | 6 | 0 | 0.449146 | 0.879515 | 0.097399 |
| 3 | 1 | 0 | -1.309533 | 2.024736 | -0.155605 |
| 4 | 1 | 0 | 1.090473 | 1.756704 | 0.096097 |
| 5 | 6 | 0 | -1.779379 | -0.067171 | -0.056314 |
| 6 | 7 | 0 | -2.511482 | -0.966911 | -0.062233 |
| 7 | 6 | 0 | 1.151850 | -0.426791 | 0.299602 |
| 8 | 1 | 0 | 0.556392 | -1.247666 | -0.125282 |
| 9 | 1 | 0 | 1.229273 | -0.609112 | 1.384704 |
| 10 | 8 | 0 | 2.425457 | -0.320736 | -0.298598 |
| 11 | 1 | 0 | 2.899167 | -1.141278 | -0.143406 |

**614**

Standard orientation:

| Center Number | Atomic Number | Atomic Type | Coordinates (Angstroms) X | Y | Z |
|---|---|---|---|---|---|
| 1 | 6 | 0 | -0.440594 | 0.857627 | 0.000033 |
| 2 | 6 | 0 | 0.882088 | 1.050689 | 0.000108 |
| 3 | 1 | 0 | -1.105517 | 1.716755 | 0.000044 |
| 4 | 1 | 0 | 1.311527 | 2.045846 | 0.000205 |
| 5 | 6 | 0 | -1.114517 | -0.482319 | -0.000117 |
| 6 | 1 | 0 | -0.787127 | -1.052848 | 0.883527 |
| 7 | 1 | 0 | -0.787087 | -1.052653 | -0.883866 |
| 8 | 8 | 0 | -2.506696 | -0.260108 | -0.000107 |
| 9 | 1 | 0 | -2.948602 | -1.112191 | -0.001105 |
| 10 | 6 | 0 | 1.797584 | -0.050107 | -0.000047 |
| 11 | 7 | 0 | 2.517573 | -0.959911 | 0.000312 |

**615**

Standard orientation:

| Center Number | Atomic Number | Atomic Type | Coordinates (Angstroms) X | Y | Z |
|---|---|---|---|---|---|
| 1 | 6 | 0 | -0.314080 | -0.125161 | 0.004575 |
| 2 | 6 | 0 | 0.803021 | 0.606511 | -0.003142 |
| 3 | 1 | 0 | -0.262350 | -1.210789 | 0.012236 |
| 4 | 1 | 0 | 0.775453 | 1.693164 | -0.011187 |
| 5 | 6 | 0 | 2.103806 | 0.011513 | -0.001225 |
| 6 | 7 | 0 | 3.165718 | -0.454485 | 0.000145 |
| 7 | 6 | 0 | -1.698551 | 0.456372 | 0.004592 |
| 8 | 1 | 0 | -1.820598 | 1.105199 | -0.877149 |
| 9 | 1 | 0 | -1.825414 | 1.092331 | 0.895038 |
| 10 | 8 | 0 | -2.611232 | -0.616716 | -0.005490 |
| 11 | 1 | 0 | -3.502444 | -0.260196 | -0.004824 |

**616**

Standard orientation:

| Center Number | Atomic Number | Atomic Type | Coordinates (Angstroms) X | Y | Z |
|---|---|---|---|---|---|
| 1 | 6 | 0 | -0.057996 | 0.369876 | 0.317302 |
| 2 | 1 | 0 | 0.149524 | 0.336542 | 1.397092 |

| | | | | | |
|---|---|---|---|---|---|
| 3 | 6 | 0 | 1.137661 | -0.151158 | -0.436543 |
| 4 | 6 | 0 | 2.202910 | -0.672651 | 0.157515 |
| 5 | 1 | 0 | 1.065660 | -0.073124 | -1.517923 |
| 6 | 1 | 0 | 3.042585 | -1.045466 | -0.418689 |
| 7 | 1 | 0 | 2.276246 | -0.749241 | 1.239055 |
| 8 | 8 | 0 | -0.317142 | 1.680416 | -0.129475 |
| 9 | 1 | 0 | -1.103801 | 2.010320 | 0.316048 |
| 10 | 6 | 0 | -1.233301 | -0.507487 | 0.077668 |
| 11 | 7 | 0 | -2.169818 | -1.164835 | -0.096491 |

**617**

Standard orientation:

| Center Number | Atomic Number | Atomic Type | Coordinates (Angstroms) X | Y | Z |
|---|---|---|---|---|---|
| 1 | 6 | 0 | -1.619135 | 0.649691 | 0.000026 |
| 2 | 6 | 0 | -0.303205 | 0.784817 | 0.000016 |
| 3 | 1 | 0 | -2.187875 | 1.573749 | 0.000045 |
| 4 | 1 | 0 | 0.250979 | 1.715175 | 0.000025 |
| 5 | 6 | 0 | -2.345967 | -0.664912 | 0.000014 |
| 6 | 1 | 0 | -2.083979 | -1.259206 | 0.880700 |
| 7 | 1 | 0 | -3.426165 | -0.513973 | 0.000004 |
| 8 | 1 | 0 | -2.083961 | -1.259200 | -0.880669 |
| 9 | 8 | 0 | 0.495687 | -0.372551 | -0.000008 |
| 10 | 6 | 0 | 1.775335 | -0.203650 | -0.000022 |
| 11 | 7 | 0 | 2.931905 | -0.095974 | -0.000035 |

**700**

Standard orientation:

| Center Number | Atomic Number | Atomic Type | Coordinates (Angstroms) X | Y | Z |
|---|---|---|---|---|---|
| 1 | 6 | 0 | -0.048779 | 0.453136 | 0.000000 |
| 2 | 8 | 0 | -0.625448 | 1.525335 | -0.000006 |
| 3 | 6 | 0 | 1.430259 | 0.393909 | 0.000009 |
| 4 | 6 | 0 | 2.164171 | -0.719851 | -0.000004 |
| 5 | 1 | 0 | 1.905201 | 1.371116 | 0.000023 |
| 6 | 1 | 0 | 3.247927 | -0.667186 | -0.000001 |
| 7 | 1 | 0 | 1.732886 | -1.715064 | -0.000023 |
| 8 | 6 | 0 | -0.882748 | -0.818477 | -0.000006 |
| 9 | 1 | 0 | -0.387033 | -1.789414 | 0.000020 |
| 10 | 7 | 0 | -2.148677 | -0.780509 | 0.000005 |
| 11 | 1 | 0 | -2.432068 | 0.209132 | 0.000000 |

**701**

Standard orientation:

| Center Number | Atomic Number | Atomic Type | Coordinates (Angstroms) X | Y | Z |
|---|---|---|---|---|---|
| 1 | 6 | 0 | 1.837926 | -0.401130 | -0.000077 |
| 2 | 8 | 0 | 2.916756 | 0.144896 | 0.000074 |
| 3 | 1 | 0 | 1.717884 | -1.502092 | -0.000324 |
| 4 | 6 | 0 | 0.556393 | 0.351519 | -0.000003 |
| 5 | 1 | 0 | 0.622007 | 1.447587 | -0.000192 |
| 6 | 7 | 0 | -0.533947 | -0.311066 | 0.000070 |

| | | | | | |
|---|---|---|---|---|---|
| 7 | 6 | 0 | -1.738602 | 0.402063 | -0.000019 |
| 8 | 6 | 0 | -2.901612 | -0.248337 | 0.000010 |
| 9 | 1 | 0 | -1.696022 | 1.494552 | -0.000092 |
| 10 | 1 | 0 | -3.844257 | 0.286393 | -0.000033 |
| 11 | 1 | 0 | -2.920663 | -1.332843 | 0.000085 |

**702**

Standard orientation:

| Center Number | Atomic Number | Atomic Type | Coordinates (Angstroms) X | Y | Z |
|---|---|---|---|---|---|
| 1 | 6 | 0 | -1.090944 | 0.744074 | -0.000184 |
| 2 | 8 | 0 | -0.633394 | 1.857244 | 0.000412 |
| 3 | 1 | 0 | -2.192809 | 0.578451 | -0.000846 |
| 4 | 6 | 0 | -0.323964 | -0.539925 | -0.000089 |
| 5 | 6 | 0 | -1.040041 | -1.671523 | -0.000300 |
| 6 | 1 | 0 | -0.568167 | -2.649728 | -0.000209 |
| 7 | 1 | 0 | -2.126506 | -1.653004 | -0.000577 |
| 8 | 6 | 0 | 1.153873 | -0.612544 | 0.000213 |
| 9 | 1 | 0 | 1.535395 | -1.644977 | 0.000756 |
| 10 | 7 | 0 | 1.905884 | 0.410426 | -0.000076 |
| 11 | 1 | 0 | 2.884513 | 0.117838 | 0.000272 |

**703**

Standard orientation:

| Center Number | Atomic Number | Atomic Type | Coordinates (Angstroms) X | Y | Z |
|---|---|---|---|---|---|
| 1 | 6 | 0 | -0.032255 | 0.498248 | 0.000003 |
| 2 | 8 | 0 | -0.511649 | 1.611849 | -0.000007 |
| 3 | 6 | 0 | 1.445616 | 0.326736 | 0.000015 |
| 4 | 6 | 0 | 2.119547 | -0.824010 | -0.000016 |
| 5 | 1 | 0 | 1.978307 | 1.274070 | 0.000041 |
| 6 | 1 | 0 | 3.204795 | -0.828107 | -0.000013 |
| 7 | 1 | 0 | 1.641088 | -1.797667 | -0.000051 |
| 8 | 6 | 0 | -0.925520 | -0.729051 | 0.000012 |
| 9 | 1 | 0 | -0.437923 | -1.709796 | 0.000098 |
| 10 | 7 | 0 | -2.183643 | -0.569505 | -0.000018 |
| 11 | 1 | 0 | -2.651905 | -1.478292 | 0.000026 |

**704**

Standard orientation:

| Center Number | Atomic Number | Atomic Type | Coordinates (Angstroms) X | Y | Z |
|---|---|---|---|---|---|
| 1 | 6 | 0 | 0.064886 | 0.681127 | 0.000068 |
| 2 | 8 | 0 | -0.056804 | 1.896227 | 0.000379 |
| 3 | 6 | 0 | 1.406834 | 0.022228 | -0.000909 |
| 4 | 6 | 0 | 1.631714 | -1.287684 | 0.000429 |
| 5 | 1 | 0 | 2.217473 | 0.744210 | -0.002622 |
| 6 | 1 | 0 | 2.641052 | -1.684683 | -0.000322 |
| 7 | 1 | 0 | 0.819910 | -2.009563 | 0.002318 |
| 8 | 7 | 0 | -0.986044 | -0.154885 | 0.001099 |
| 9 | 6 | 0 | -2.000172 | -0.870288 | -0.000757 |
| 10 | 1 | 0 | -2.470251 | -1.202344 | -0.935585 |

| | | | | | |
|---|---|---|---|---|---|
| 11 | 1 | 0 | -2.471022 | -1.205543 | 0.932507 |

**800**

Standard orientation:

| Center Number | Atomic Number | Atomic Type | Coordinates (Angstroms) X | Y | Z |
|---|---|---|---|---|---|
| 1 | 6 | 0 | -1.575497 | -0.074670 | -0.345080 |
| 2 | 8 | 0 | -2.556465 | -0.647574 | 0.074411 |
| 3 | 1 | 0 | -1.408696 | 0.134911 | -1.420331 |
| 4 | 7 | 0 | -0.568642 | 0.409869 | 0.435859 |
| 5 | 1 | 0 | -0.613087 | 0.165842 | 1.415251 |
| 6 | 6 | 0 | 0.670833 | 0.943524 | -0.090812 |
| 7 | 1 | 0 | 0.945103 | 1.849126 | 0.460598 |
| 8 | 1 | 0 | 0.490275 | 1.255106 | -1.125436 |
| 9 | 6 | 0 | 1.788535 | -0.009317 | -0.054660 |
| 10 | 6 | 0 | 2.701906 | -0.792732 | -0.009561 |
| 11 | 1 | 0 | 3.503957 | -1.494306 | 0.024300 |

**801**

Standard orientation:

| Center Number | Atomic Number | Atomic Type | Coordinates (Angstroms) X | Y | Z |
|---|---|---|---|---|---|
| 1 | 6 | 0 | -2.667090 | -0.414271 | 0.000063 |
| 2 | 6 | 0 | -1.516664 | -0.051696 | 0.000007 |
| 3 | 1 | 0 | -3.690330 | -0.716258 | 0.000103 |
| 4 | 6 | 0 | -0.109446 | 0.335181 | -0.000027 |
| 5 | 8 | 0 | 0.235845 | 1.497305 | -0.000020 |
| 6 | 6 | 0 | 0.890100 | -0.818630 | -0.000128 |
| 7 | 1 | 0 | 0.672762 | -1.447863 | 0.872485 |
| 8 | 1 | 0 | 0.672879 | -1.447504 | -0.873040 |
| 9 | 7 | 0 | 2.279362 | -0.427728 | 0.000078 |
| 10 | 1 | 0 | 2.460511 | 0.162166 | -0.806279 |
| 11 | 1 | 0 | 2.460485 | 0.161613 | 0.806848 |

**802**

Standard orientation:

| Center Number | Atomic Number | Atomic Type | Coordinates (Angstroms) X | Y | Z |
|---|---|---|---|---|---|
| 1 | 6 | 0 | 0.502462 | 0.764995 | 0.373558 |
| 2 | 1 | 0 | 1.080042 | 1.670279 | 0.190425 |
| 3 | 1 | 0 | 0.238532 | 0.706394 | 1.436247 |
| 4 | 8 | 0 | -0.702332 | 0.893576 | -0.404242 |
| 5 | 6 | 0 | -1.630511 | 0.023518 | -0.120013 |
| 6 | 6 | 0 | -2.495124 | -0.775932 | 0.133251 |
| 7 | 1 | 0 | -3.276341 | -1.465328 | 0.347408 |
| 8 | 6 | 0 | 1.292866 | -0.448047 | -0.037617 |
| 9 | 1 | 0 | 0.694257 | -1.330668 | -0.300717 |
| 10 | 7 | 0 | 2.559012 | -0.401573 | -0.033217 |
| 11 | 1 | 0 | 2.950923 | -1.305477 | -0.301988 |

**900**

Standard orientation:

| Center Number | Atomic Number | Atomic Type | Coordinates (Angstroms) X | Y | Z |
|---|---|---|---|---|---|
| 1 | 6 | 0 | -0.980720 | -0.113307 | 0.000101 |
| 2 | 6 | 0 | 0.281380 | -0.869088 | -0.000079 |
| 3 | 1 | 0 | 0.245271 | -1.952104 | -0.000341 |
| 4 | 7 | 0 | -2.064366 | -0.787719 | 0.000001 |
| 5 | 1 | 0 | -2.867591 | -0.157463 | -0.000050 |
| 6 | 6 | 0 | 1.473124 | -0.299954 | 0.000046 |
| 7 | 8 | 0 | 2.524667 | 0.199413 | 0.000132 |
| 8 | 6 | 0 | -0.873898 | 1.395764 | -0.000103 |
| 9 | 1 | 0 | -0.330120 | 1.745478 | 0.883799 |
| 10 | 1 | 0 | -0.330212 | 1.745252 | -0.884153 |
| 11 | 1 | 0 | -1.863437 | 1.857077 | -0.000110 |

**901**

Standard orientation:

| Center Number | Atomic Number | Atomic Type | Coordinates (Angstroms) X | Y | Z |
|---|---|---|---|---|---|
| 1 | 6 | 0 | 0.819488 | -0.700680 | 0.000667 |
| 2 | 8 | 0 | 1.409216 | -1.700179 | -0.000409 |
| 3 | 6 | 0 | 0.166548 | 0.457297 | 0.000716 |
| 4 | 6 | 0 | -1.294811 | 0.382746 | 0.000136 |
| 5 | 1 | 0 | -1.777748 | 1.370338 | -0.000196 |
| 6 | 7 | 0 | -1.931503 | -0.723004 | -0.000175 |
| 7 | 1 | 0 | -2.936072 | -0.555785 | -0.000739 |
| 8 | 6 | 0 | 0.920837 | 1.772227 | -0.000303 |
| 9 | 1 | 0 | 1.546510 | 1.897458 | 0.887706 |
| 10 | 1 | 0 | 1.549945 | 1.894161 | -0.886334 |
| 11 | 1 | 0 | 0.191787 | 2.586755 | -0.003232 |

**902**

Standard orientation:

| Center Number | Atomic Number | Atomic Type | Coordinates (Angstroms) X | Y | Z |
|---|---|---|---|---|---|
| 1 | 6 | 0 | -1.823409 | 0.014029 | -0.150415 |
| 2 | 6 | 0 | -0.634206 | 0.483835 | -0.454741 |
| 3 | 1 | 0 | -0.505960 | 0.861621 | -1.462795 |
| 4 | 6 | 0 | 0.529367 | 0.490439 | 0.513840 |
| 5 | 1 | 0 | 0.959599 | 1.492392 | 0.597275 |
| 6 | 1 | 0 | 0.186519 | 0.191294 | 1.510969 |
| 7 | 6 | 0 | 1.617293 | -0.453540 | 0.069125 |
| 8 | 1 | 0 | 1.298931 | -1.497580 | -0.064550 |
| 9 | 7 | 0 | 2.796268 | -0.036234 | -0.143970 |
| 10 | 1 | 0 | 3.397882 | -0.807466 | -0.439396 |
| 11 | 8 | 0 | -2.880639 | -0.399401 | 0.124929 |

**903**

Standard orientation:

| Center | Atomic | Atomic | Coordinates (Angstroms) |
|---|---|---|---|

| Number | Number | Type | X | Y | Z |
|---|---|---|---|---|---|
| 1 | 6 | 0 | 1.282785 | 0.162759 | 0.000076 |
| 2 | 8 | 0 | 2.177635 | -0.584221 | 0.000165 |
| 3 | 6 | 0 | 0.426260 | 1.181137 | -0.000270 |
| 4 | 1 | 0 | 0.898673 | 2.156708 | -0.000727 |
| 5 | 6 | 0 | -1.021909 | 1.053173 | 0.000123 |
| 6 | 6 | 0 | -1.659344 | -0.121398 | 0.000180 |
| 7 | 1 | 0 | -1.590274 | 1.977893 | 0.000373 |
| 8 | 1 | 0 | -2.746710 | -0.177981 | 0.000485 |
| 9 | 7 | 0 | -0.866435 | -1.309831 | -0.000232 |
| 10 | 1 | 0 | -1.041964 | -1.884133 | 0.817945 |
| 11 | 1 | 0 | -1.042515 | -1.883936 | -0.818429 |

**904**

Standard orientation:

| Center Number | Atomic Number | Atomic Type | Coordinates (Angstroms) X | Y | Z |
|---|---|---|---|---|---|
| 1 | 6 | 0 | 1.701629 | 0.003973 | 0.005271 |
| 2 | 6 | 0 | 0.827885 | 1.002366 | 0.022293 |
| 3 | 1 | 0 | 1.231367 | 2.006661 | 0.056566 |
| 4 | 7 | 0 | -0.569439 | 0.853348 | -0.036719 |
| 5 | 6 | 0 | -1.129690 | -0.286732 | 0.061206 |
| 6 | 1 | 0 | -0.563000 | -1.218638 | 0.213444 |
| 7 | 6 | 0 | -2.615407 | -0.424567 | -0.026194 |
| 8 | 1 | 0 | -2.893832 | -1.075769 | -0.862459 |
| 9 | 1 | 0 | -3.015219 | -0.884884 | 0.884218 |
| 10 | 1 | 0 | -3.074273 | 0.555398 | -0.166268 |
| 11 | 8 | 0 | 2.449316 | -0.890806 | -0.030490 |

**905**

Standard orientation:

| Center Number | Atomic Number | Atomic Type | Coordinates (Angstroms) X | Y | Z |
|---|---|---|---|---|---|
| 1 | 6 | 0 | -1.759530 | -0.008851 | -0.000008 |
| 2 | 6 | 0 | -0.887862 | 0.983282 | -0.000004 |
| 3 | 1 | 0 | -1.322648 | 1.976049 | 0.000071 |
| 4 | 8 | 0 | -2.533370 | -0.881271 | 0.000000 |
| 5 | 6 | 0 | 0.567592 | 0.812235 | -0.000006 |
| 6 | 6 | 0 | 1.243163 | -0.339634 | 0.000009 |
| 7 | 1 | 0 | 1.144273 | 1.732331 | -0.000021 |
| 8 | 1 | 0 | 0.707529 | -1.294946 | 0.000031 |
| 9 | 7 | 0 | 2.672559 | -0.338880 | -0.000001 |
| 10 | 1 | 0 | 3.024863 | -0.836657 | 0.812382 |
| 11 | 1 | 0 | 3.024851 | -0.836643 | -0.812399 |

**906**

Standard orientation:

| Center Number | Atomic Number | Atomic Type | Coordinates (Angstroms) X | Y | Z |
|---|---|---|---|---|---|
| 1 | 6 | 0 | 0.826106 | -0.683325 | -0.001919 |
| 2 | 6 | 0 | 0.155719 | 0.462847 | 0.003733 |

| | | | | | |
|---|---|---|---|---|---|
| 3 | 7 | 0 | -1.250990 | 0.482627 | 0.051375 |
| 4 | 6 | 0 | -1.969610 | -0.564348 | -0.028206 |
| 5 | 1 | 0 | -1.583434 | -1.584695 | -0.151632 |
| 6 | 1 | 0 | -3.049544 | -0.447923 | 0.030446 |
| 7 | 8 | 0 | 1.366395 | -1.718670 | 0.012895 |
| 8 | 6 | 0 | 0.872870 | 1.787161 | -0.018896 |
| 9 | 1 | 0 | 0.589792 | 2.378083 | 0.857084 |
| 10 | 1 | 0 | 1.958315 | 1.659323 | -0.011676 |
| 11 | 1 | 0 | 0.600137 | 2.352170 | -0.915277 |

**907**

Standard orientation:

| Center Number | Atomic Number | Atomic Type | Coordinates (Angstroms) X | Y | Z |
|---|---|---|---|---|---|
| 1 | 7 | 0 | 2.519731 | -0.472251 | -0.091951 |
| 2 | 1 | 0 | 2.986247 | -0.601342 | 0.808668 |
| 3 | 6 | 0 | 1.341269 | -0.140516 | -0.010537 |
| 4 | 6 | 0 | 0.059433 | 0.197180 | -0.017112 |
| 5 | 8 | 0 | -0.264308 | 1.537743 | 0.077637 |
| 6 | 1 | 0 | -0.734021 | 1.777144 | -0.729327 |
| 7 | 6 | 0 | -1.002039 | -0.804952 | -0.052689 |
| 8 | 6 | 0 | -2.299298 | -0.511566 | 0.063752 |
| 9 | 1 | 0 | -0.681893 | -1.833259 | -0.201669 |
| 10 | 1 | 0 | -3.055275 | -1.285847 | 0.009779 |
| 11 | 1 | 0 | -2.634898 | 0.506238 | 0.234630 |

**908**

Standard orientation:

| Center Number | Atomic Number | Atomic Type | Coordinates (Angstroms) X | Y | Z |
|---|---|---|---|---|---|
| 1 | 6 | 0 | -1.273394 | -0.115014 | 0.000007 |
| 2 | 6 | 0 | -2.520495 | -0.497820 | 0.000023 |
| 3 | 1 | 0 | -3.063699 | -0.666768 | 0.927924 |
| 4 | 1 | 0 | -3.063687 | -0.666651 | -0.927917 |
| 5 | 6 | 0 | -0.006952 | 0.253903 | -0.000037 |
| 6 | 6 | 0 | 1.051054 | -0.774117 | -0.000029 |
| 7 | 1 | 0 | 0.663840 | -1.800235 | -0.000114 |
| 8 | 7 | 0 | 2.281500 | -0.458516 | 0.000032 |
| 9 | 1 | 0 | 2.854837 | -1.303241 | 0.000066 |
| 10 | 8 | 0 | 0.433536 | 1.540197 | -0.000001 |
| 11 | 1 | 0 | -0.331354 | 2.123212 | 0.000050 |

**909**

Standard orientation:

| Center Number | Atomic Number | Atomic Type | Coordinates (Angstroms) X | Y | Z |
|---|---|---|---|---|---|
| 1 | 6 | 0 | 0.519764 | -0.386565 | 0.051576 |
| 2 | 6 | 0 | 1.790835 | -0.302105 | 0.377208 |
| 3 | 1 | 0 | 2.169188 | -0.860398 | 1.229789 |
| 4 | 7 | 0 | -0.657746 | -0.527909 | -0.279555 |
| 5 | 8 | 0 | 2.643710 | 0.569721 | -0.273162 |
| 6 | 1 | 0 | 3.283076 | 0.046320 | -0.766080 |

| | | | | | |
|---|---|---|---|---|---|
| 7 | 6 | 0 | -1.665355 | 0.315405 | 0.243777 |
| 8 | 6 | 0 | -2.932193 | 0.154565 | -0.124392 |
| 9 | 1 | 0 | -1.358010 | 1.087209 | 0.948975 |
| 10 | 1 | 0 | -3.709574 | 0.798089 | 0.270168 |
| 11 | 1 | 0 | -3.208442 | -0.621423 | -0.829680 |

**1000**

Standard orientation:

| Center Number | Atomic Number | Atomic Type | Coordinates (Angstroms) X | Y | Z |
|---|---|---|---|---|---|
| 1 | 6 | 0 | -0.788207 | 0.193181 | 0.000119 |
| 2 | 8 | 0 | -0.577583 | 1.378647 | -0.000036 |
| 3 | 6 | 0 | -2.184000 | -0.394806 | -0.000006 |
| 4 | 1 | 0 | -2.719703 | -0.032709 | -0.881139 |
| 5 | 1 | 0 | -2.188670 | -1.487423 | 0.000008 |
| 6 | 1 | 0 | -2.719865 | -0.032685 | 0.881011 |
| 7 | 6 | 0 | 0.346735 | -0.837708 | -0.000022 |
| 8 | 1 | 0 | 0.232929 | -1.481949 | 0.880407 |
| 9 | 1 | 0 | 0.232860 | -1.481695 | -0.880649 |
| 10 | 7 | 0 | 1.625558 | -0.248313 | 0.000013 |
| 11 | 6 | 0 | 2.692839 | 0.243579 | 0.000002 |

**1001**

Standard orientation:

| Center Number | Atomic Number | Atomic Type | Coordinates (Angstroms) X | Y | Z |
|---|---|---|---|---|---|
| 1 | 6 | 0 | 0.754971 | -0.090057 | -0.000007 |
| 2 | 6 | 0 | -0.372679 | -0.816601 | -0.000003 |
| 3 | 1 | 0 | -0.342427 | -1.898927 | 0.000000 |
| 4 | 8 | 0 | 1.931247 | -0.765278 | 0.000006 |
| 5 | 1 | 0 | 2.660621 | -0.140472 | -0.000005 |
| 6 | 6 | 0 | 0.822187 | 1.403508 | 0.000000 |
| 7 | 1 | 0 | 1.358061 | 1.759284 | 0.887176 |
| 8 | 1 | 0 | 1.358181 | 1.759283 | -0.887104 |
| 9 | 1 | 0 | -0.174132 | 1.844200 | -0.000068 |
| 10 | 7 | 0 | -1.619465 | -0.230578 | -0.000002 |
| 11 | 6 | 0 | -2.700149 | 0.238634 | 0.000005 |

**1002**

Standard orientation:

| Center Number | Atomic Number | Atomic Type | Coordinates (Angstroms) X | Y | Z |
|---|---|---|---|---|---|
| 1 | 6 | 0 | 0.990941 | -0.735017 | 0.000965 |
| 2 | 6 | 0 | -0.313978 | -0.462355 | -0.000032 |
| 3 | 1 | 0 | 1.260333 | -1.785300 | 0.002252 |
| 4 | 7 | 0 | -0.782481 | 0.842183 | -0.000813 |
| 5 | 6 | 0 | -1.154408 | 1.962029 | 0.000559 |
| 6 | 8 | 0 | -1.274835 | -1.420850 | -0.001329 |
| 7 | 1 | 0 | -2.141537 | -1.005958 | 0.007993 |
| 8 | 6 | 0 | 2.063264 | 0.315778 | -0.000207 |
| 9 | 1 | 0 | 3.051739 | -0.145943 | 0.007161 |
| 10 | 1 | 0 | 1.999498 | 0.957821 | -0.885372 |

| | | | | | |
|---|---|---|---|---|---|
| 11 | 1 | 0 | 1.991107 | 0.968298 | 0.876579 |

**1003**

Standard orientation:

| Center Number | Atomic Number | Atomic Type | Coordinates (Angstroms) X | Y | Z |
|---|---|---|---|---|---|
| 1 | 6 | 0 | -2.019454 | -0.539323 | -0.372232 |
| 2 | 6 | 0 | -0.883354 | 0.018850 | 0.034892 |
| 3 | 1 | 0 | -2.109850 | -1.615222 | -0.449186 |
| 4 | 1 | 0 | -2.878470 | 0.074042 | -0.613756 |
| 5 | 6 | 0 | 0.316662 | -0.798836 | 0.463632 |
| 6 | 1 | 0 | 0.388996 | -0.809517 | 1.557161 |
| 7 | 1 | 0 | 0.221306 | -1.828438 | 0.114324 |
| 8 | 7 | 0 | 1.526950 | -0.243195 | -0.041664 |
| 9 | 6 | 0 | 2.527338 | 0.216081 | -0.459326 |
| 10 | 8 | 0 | -0.765384 | 1.361360 | 0.190582 |
| 11 | 1 | 0 | 0.165286 | 1.609983 | 0.156649 |

**1004**

Standard orientation:

| Center Number | Atomic Number | Atomic Type | Coordinates (Angstroms) X | Y | Z |
|---|---|---|---|---|---|
| 1 | 6 | 0 | 1.481192 | 0.116715 | 0.308952 |
| 2 | 6 | 0 | 0.409939 | 0.168871 | -0.478282 |
| 3 | 1 | 0 | 1.468202 | 0.541483 | 1.312818 |
| 4 | 1 | 0 | 0.440301 | -0.273417 | -1.468741 |
| 5 | 6 | 0 | -0.860912 | 0.831070 | -0.036145 |
| 6 | 1 | 0 | -0.730458 | 1.310863 | 0.939536 |
| 7 | 1 | 0 | -1.164404 | 1.611325 | -0.742628 |
| 8 | 7 | 0 | -1.948581 | -0.092399 | 0.068008 |
| 9 | 8 | 0 | 2.642957 | -0.452260 | -0.092928 |
| 10 | 1 | 0 | 3.280648 | -0.428341 | 0.622975 |
| 11 | 6 | 0 | -2.829866 | -0.866161 | 0.139378 |

**1005**

Standard orientation:

| Center Number | Atomic Number | Atomic Type | Coordinates (Angstroms) X | Y | Z |
|---|---|---|---|---|---|
| 1 | 6 | 0 | -1.670307 | 0.605345 | 0.000188 |
| 2 | 6 | 0 | -0.345155 | 0.705959 | 0.000484 |
| 3 | 1 | 0 | -2.313917 | 1.481062 | 0.000223 |
| 4 | 1 | 0 | 0.117673 | 1.684420 | 0.000768 |
| 5 | 8 | 0 | -2.278998 | -0.612629 | -0.000166 |
| 6 | 1 | 0 | -3.231503 | -0.502362 | -0.000429 |
| 7 | 6 | 0 | 0.543339 | -0.512622 | 0.000428 |
| 8 | 1 | 0 | 0.347079 | -1.137889 | -0.877825 |
| 9 | 1 | 0 | 0.347812 | -1.137304 | 0.879264 |
| 10 | 7 | 0 | 1.922198 | -0.161038 | -0.000236 |
| 11 | 6 | 0 | 3.057032 | 0.141378 | -0.000936 |

**1006**

Standard orientation:

| Center Number | Atomic Number | Atomic Type | Coordinates (Angstroms) X | Y | Z |
|---|---|---|---|---|---|
| 1 | 6 | 0 | 0.798536 | 1.730156 | 0.199108 |
| 2 | 6 | 0 | 0.272029 | 0.538925 | -0.072397 |
| 3 | 1 | 0 | 1.850578 | 1.846628 | 0.429744 |
| 4 | 1 | 0 | 0.170560 | 2.613004 | 0.191538 |
| 5 | 7 | 0 | 1.054912 | -0.609489 | -0.051171 |
| 6 | 6 | 0 | 1.633022 | -1.636634 | -0.038025 |
| 7 | 6 | 0 | -1.173007 | 0.273395 | -0.434964 |
| 8 | 1 | 0 | -1.733425 | 1.206200 | -0.346027 |
| 9 | 1 | 0 | -1.216993 | -0.048877 | -1.486499 |
| 10 | 8 | 0 | -1.786344 | -0.659674 | 0.418399 |
| 11 | 1 | 0 | -1.347826 | -1.508194 | 0.299919 |

**1007**

Standard orientation:

| Center Number | Atomic Number | Atomic Type | Coordinates (Angstroms) X | Y | Z |
|---|---|---|---|---|---|
| 1 | 6 | 0 | -2.114292 | -0.455110 | -0.311603 |
| 2 | 6 | 0 | -0.959711 | -0.742926 | 0.272217 |
| 3 | 1 | 0 | -2.319259 | 0.539645 | -0.690656 |
| 4 | 1 | 0 | -2.881432 | -1.212781 | -0.428075 |
| 5 | 1 | 0 | -0.740549 | -1.736682 | 0.652440 |
| 6 | 6 | 0 | 0.141663 | 0.265824 | 0.478247 |
| 7 | 1 | 0 | 0.292438 | 0.420416 | 1.557527 |
| 8 | 8 | 0 | -0.174069 | 1.457269 | -0.164564 |
| 9 | 1 | 0 | 0.491807 | 2.113051 | 0.060786 |
| 10 | 7 | 0 | 1.385802 | -0.273805 | -0.011743 |
| 11 | 6 | 0 | 2.407163 | -0.711983 | -0.397747 |

**1008**

Standard orientation:

| Center Number | Atomic Number | Atomic Type | Coordinates (Angstroms) X | Y | Z |
|---|---|---|---|---|---|
| 1 | 6 | 0 | 0.932647 | 0.992608 | 0.000032 |
| 2 | 6 | 0 | -0.391383 | 0.847774 | -0.000276 |
| 3 | 1 | 0 | 1.424491 | 1.957798 | -0.000306 |
| 4 | 1 | 0 | -1.016672 | 1.734557 | -0.001080 |
| 5 | 6 | 0 | -1.099536 | -0.473302 | 0.000224 |
| 6 | 1 | 0 | -0.786696 | -1.053489 | -0.882787 |
| 7 | 1 | 0 | -0.786936 | -1.052741 | 0.883814 |
| 8 | 8 | 0 | -2.486603 | -0.218449 | -0.000071 |
| 9 | 1 | 0 | -2.947322 | -1.060439 | -0.000229 |
| 10 | 7 | 0 | 1.779111 | -0.100861 | 0.000864 |
| 11 | 6 | 0 | 2.483636 | -1.045758 | -0.000795 |

**1009**

Standard orientation:

| Center | Atomic | Atomic | Coordinates (Angstroms) |
|---|---|---|---|

| Number | Number | Type | X | Y | Z |
|---|---|---|---|---|---|
| 1 | 6 | 0 | -0.841284 | 0.567864 | 0.000002 |
| 2 | 6 | 0 | 0.283004 | -0.144638 | -0.000007 |
| 3 | 1 | 0 | -0.852925 | 1.654110 | 0.000032 |
| 4 | 1 | 0 | 0.241834 | -1.229929 | -0.000037 |
| 5 | 7 | 0 | -2.092260 | -0.016756 | -0.000027 |
| 6 | 6 | 0 | -3.176586 | -0.477813 | -0.000051 |
| 7 | 6 | 0 | 1.657106 | 0.464852 | 0.000022 |
| 8 | 1 | 0 | 1.777376 | 1.107947 | -0.886144 |
| 9 | 1 | 0 | 1.777342 | 1.107942 | 0.886195 |
| 10 | 8 | 0 | 2.587116 | -0.595192 | 0.000037 |
| 11 | 1 | 0 | 3.471823 | -0.222835 | 0.000053 |

**1100**

Standard orientation:

| Center Number | Atomic Number | Atomic Type | Coordinates (Angstroms) X | Y | Z |
|---|---|---|---|---|---|
| 1 | 6 | 0 | 0.064713 | 1.272442 | 0.015189 |
| 2 | 6 | 0 | -1.419830 | 0.948918 | -0.071505 |
| 3 | 6 | 0 | -0.179041 | -1.176581 | -0.063588 |
| 4 | 1 | 0 | 0.375617 | 1.919562 | -0.842989 |
| 5 | 1 | 0 | 0.285801 | 1.841757 | 0.952947 |
| 6 | 1 | 0 | -0.040988 | -1.873777 | 0.800480 |
| 7 | 1 | 0 | -0.033885 | -1.776540 | -0.997061 |
| 8 | 7 | 0 | -1.572451 | -0.564896 | -0.037793 |
| 9 | 1 | 0 | -2.049227 | -0.838923 | 0.797427 |
| 10 | 6 | 0 | 0.833009 | -0.041484 | -0.001146 |
| 11 | 8 | 0 | 2.084592 | -0.167197 | 0.035007 |

**1101**

Standard orientation:

| Center Number | Atomic Number | Atomic Type | Coordinates (Angstroms) X | Y | Z |
|---|---|---|---|---|---|
| 1 | 6 | 0 | 1.719845 | -0.392737 | 0.078555 |
| 2 | 8 | 0 | 2.911213 | -0.249616 | -0.116572 |
| 3 | 1 | 0 | 1.294012 | -1.364592 | 0.414770 |
| 4 | 6 | 0 | 0.732499 | 0.654130 | -0.095276 |
| 5 | 1 | 0 | 1.009675 | 1.658832 | -0.389608 |
| 6 | 7 | 0 | -0.523184 | 0.387641 | 0.121248 |
| 7 | 6 | 0 | -1.646401 | 0.147856 | 0.449311 |
| 8 | 6 | 0 | -2.865029 | -0.344849 | -0.232179 |
| 9 | 1 | 0 | -3.229646 | -1.233614 | 0.287911 |
| 10 | 1 | 0 | -3.646461 | 0.413874 | -0.142277 |
| 11 | 1 | 0 | -2.700487 | -0.577464 | -1.289426 |

**1102**

Standard orientation:

| Center Number | Atomic Number | Atomic Type | Coordinates (Angstroms) X | Y | Z |
|---|---|---|---|---|---|
| 1 | 6 | 0 | 1.465917 | 0.628438 | 0.000032 |
| 2 | 6 | 0 | 0.061049 | 1.131428 | 0.000090 |

| | | | | | |
|---|---|---|---|---|---|
| 3 | 6 | 0 | -0.752455 | 0.059017 | -0.000241 |
| 4 | 6 | 0 | -0.013166 | -1.230155 | 0.000145 |
| 5 | 1 | 0 | 2.037868 | 0.927011 | -0.887516 |
| 6 | 1 | 0 | 2.038476 | 0.927121 | 0.887162 |
| 7 | 1 | 0 | -0.208392 | 2.178127 | 0.000271 |
| 8 | 7 | 0 | 1.241836 | -0.819811 | -0.000085 |
| 9 | 1 | 0 | 2.017720 | -1.465109 | 0.000178 |
| 10 | 8 | 0 | -2.098137 | 0.063037 | 0.000018 |
| 11 | 1 | 0 | -2.361502 | -0.865132 | 0.000198 |

**1103**

Standard orientation:

| Center Number | Atomic Number | Atomic Type | Coordinates (Angstroms) X | Y | Z |
|---|---|---|---|---|---|
| 1 | 6 | 0 | 1.460920 | 0.912153 | -0.000071 |
| 2 | 6 | 0 | 0.011275 | 1.190797 | 0.000095 |
| 3 | 6 | 0 | -0.702288 | 0.045792 | -0.000021 |
| 4 | 6 | 0 | 0.228229 | -1.125862 | 0.000099 |
| 5 | 7 | 0 | 1.503020 | -0.424444 | -0.000031 |
| 6 | 1 | 0 | 2.373352 | -0.932414 | -0.000497 |
| 7 | 1 | 0 | -0.404074 | 2.192215 | 0.000106 |
| 8 | 1 | 0 | 0.095990 | -1.753146 | -0.888658 |
| 9 | 8 | 0 | -2.022484 | -0.195715 | -0.000147 |
| 10 | 1 | 0 | -2.491385 | 0.645049 | 0.000226 |
| 11 | 1 | 0 | 0.096032 | -1.752161 | 0.889602 |

**1104**

Standard orientation:

| Center Number | Atomic Number | Atomic Type | Coordinates (Angstroms) X | Y | Z |
|---|---|---|---|---|---|
| 1 | 6 | 0 | 1.953653 | -0.233209 | 0.144131 |
| 2 | 8 | 0 | 2.463576 | 0.865448 | 0.075619 |
| 3 | 1 | 0 | 2.452693 | -1.094164 | 0.636988 |
| 4 | 6 | 0 | 0.713638 | -0.607580 | -0.520679 |
| 5 | 7 | 0 | -0.429198 | -0.549648 | -0.094531 |
| 6 | 6 | 0 | -1.654393 | -0.492226 | 0.235803 |
| 7 | 1 | 0 | -2.077618 | -1.364043 | 0.727058 |
| 8 | 6 | 0 | -2.498710 | 0.717149 | -0.029338 |
| 9 | 1 | 0 | -3.366620 | 0.452359 | -0.642420 |
| 10 | 1 | 0 | -2.875534 | 1.142068 | 0.906909 |
| 11 | 1 | 0 | -1.922264 | 1.482930 | -0.551280 |

**1105**

Standard orientation:

| Center Number | Atomic Number | Atomic Type | Coordinates (Angstroms) X | Y | Z |
|---|---|---|---|---|---|
| 1 | 6 | 0 | -1.387537 | 0.959964 | -0.000049 |
| 2 | 6 | 0 | -1.537532 | -0.556947 | 0.000079 |
| 3 | 6 | 0 | -0.154944 | -1.154653 | -0.000015 |
| 4 | 6 | 0 | 0.683433 | -0.115068 | 0.000042 |
| 5 | 1 | 0 | -2.139138 | -0.841717 | -0.873552 |
| 6 | 1 | 0 | -2.139207 | -0.841555 | 0.873683 |

| | | | | | |
|---|---|---|---|---|---|
| 7 | 1 | 0 | 0.104847 | -2.203190 | -0.000360 |
| 8 | 8 | 0 | 2.020445 | 0.036385 | 0.000087 |
| 9 | 1 | 0 | 2.432571 | -0.831938 | -0.000348 |
| 10 | 7 | 0 | -0.064517 | 1.091645 | -0.000038 |
| 11 | 1 | 0 | 0.408457 | 1.986028 | -0.000189 |

**1106**

Standard orientation:

| Center Number | Atomic Number | Atomic Type | Coordinates (Angstroms) X | Y | Z |
|---|---|---|---|---|---|
| 1 | 6 | 0 | 0.070297 | 0.334630 | -0.000209 |
| 2 | 8 | 0 | 0.366639 | 1.541488 | -0.000023 |
| 3 | 6 | 0 | 1.085944 | -0.766814 | -0.000308 |
| 4 | 6 | 0 | 2.382043 | -0.473477 | 0.000383 |
| 5 | 1 | 0 | 0.728977 | -1.795836 | -0.000921 |
| 6 | 1 | 0 | 3.145548 | -1.243547 | 0.000394 |
| 7 | 1 | 0 | 2.689804 | 0.567750 | 0.001001 |
| 8 | 6 | 0 | -1.237513 | -0.094701 | -0.000739 |
| 9 | 7 | 0 | -2.469083 | -0.401634 | 0.000445 |
| 10 | 1 | 0 | -3.009915 | -0.523581 | -0.856827 |
| 11 | 1 | 0 | -3.008571 | -0.523080 | 0.858662 |

**1200**

Standard orientation:

| Center Number | Atomic Number | Atomic Type | Coordinates (Angstroms) X | Y | Z |
|---|---|---|---|---|---|
| 1 | 6 | 0 | 1.472866 | -0.609498 | 0.000145 |
| 2 | 6 | 0 | 1.396886 | 0.732879 | 0.000030 |
| 3 | 6 | 0 | 0.006480 | 1.139405 | -0.000028 |
| 4 | 1 | 0 | 2.362798 | -1.222194 | -0.000409 |
| 5 | 1 | 0 | 2.224262 | 1.429924 | -0.000375 |
| 6 | 1 | 0 | -0.430554 | 2.125929 | 0.000465 |
| 7 | 6 | 0 | 0.080133 | -1.148635 | 0.000049 |
| 8 | 1 | 0 | -0.182848 | -1.730533 | -0.887289 |
| 9 | 1 | 0 | -0.183385 | -1.730141 | 0.887466 |
| 10 | 7 | 0 | -0.767135 | 0.072256 | -0.000129 |
| 11 | 8 | 0 | -2.019816 | -0.007960 | -0.000016 |

**1201**

Standard orientation:

| Center Number | Atomic Number | Atomic Type | Coordinates (Angstroms) X | Y | Z |
|---|---|---|---|---|---|
| 1 | 6 | 0 | 1.312000 | 0.819740 | -0.000316 |
| 2 | 6 | 0 | 0.022377 | 1.141230 | 0.000002 |
| 3 | 6 | 0 | 0.005656 | -1.111665 | -0.000295 |
| 4 | 1 | 0 | 2.147547 | 1.505240 | -0.000636 |
| 5 | 1 | 0 | -0.505429 | 2.082653 | 0.000480 |

| | | | | | |
|---|---|---|---|---|---|
| 6 | 1 | 0 | -0.410427 | -2.106316 | -0.000432 |
| 7 | 6 | 0 | 1.431297 | -0.679420 | 0.000210 |
| 8 | 1 | 0 | 1.980409 | -1.040176 | -0.879282 |
| 9 | 1 | 0 | 1.978704 | -1.037634 | 0.881993 |
| 10 | 7 | 0 | -0.784661 | -0.060104 | -0.000136 |
| 11 | 8 | 0 | -2.040770 | -0.000294 | 0.000153 |

**1300**

Standard orientation:

| Center Number | Atomic Number | Atomic Type | Coordinates (Angstroms) X | Y | Z |
|---|---|---|---|---|---|
| 1 | 6 | 0 | 2.482800 | -1.155266 | 0.000001 |
| 2 | 6 | 0 | 1.292037 | -0.554672 | 0.000017 |
| 3 | 1 | 0 | 2.581827 | -2.235149 | 0.000022 |
| 4 | 1 | 0 | 3.404069 | -0.576976 | -0.000035 |
| 5 | 1 | 0 | 0.361591 | -1.115525 | 0.000053 |
| 6 | 6 | 0 | 1.204143 | 0.913601 | -0.000014 |
| 7 | 1 | 0 | 2.177044 | 1.445591 | 0.000007 |
| 8 | 8 | 0 | 0.164355 | 1.542999 | -0.000004 |
| 9 | 1 | 0 | -1.611433 | 0.638443 | 0.000014 |
| 10 | 6 | 0 | -2.457517 | -0.036679 | 0.000002 |
| 11 | 7 | 0 | -3.336674 | -0.786040 | -0.000010 |

**1301**

Standard orientation:

| Center Number | Atomic Number | Atomic Type | Coordinates (Angstroms) X | Y | Z |
|---|---|---|---|---|---|
| 1 | 6 | 0 | 2.409629 | -1.089566 | -0.000009 |
| 2 | 6 | 0 | 1.196060 | -0.535300 | -0.000027 |
| 3 | 1 | 0 | 2.548641 | -2.165015 | -0.000023 |
| 4 | 1 | 0 | 3.308771 | -0.477470 | 0.000031 |
| 5 | 1 | 0 | 0.285859 | -1.128309 | -0.000064 |
| 6 | 6 | 0 | 1.059168 | 0.926661 | 0.000022 |
| 7 | 1 | 0 | 2.008813 | 1.496098 | 0.000013 |
| 8 | 8 | 0 | -0.002304 | 1.524585 | -0.000038 |
| 9 | 1 | 0 | -1.525319 | 0.586146 | -0.000107 |
| 10 | 6 | 0 | -3.107127 | -0.934096 | 0.000103 |
| 11 | 7 | 0 | -2.279245 | -0.102047 | -0.000010 |
